# Design of Promising Thiazoloindazole-Based Acetylcholinesterase Inhibitors Guided by Molecular Docking and Experimental Insights

**DOI:** 10.1021/acschemneuro.4c00241

**Published:** 2024-07-22

**Authors:** Fatima Ezzahra Laghchioua, Carlos F. M. da Silva, Diana C. G. A. Pinto, José A. S. Cavaleiro, Ricardo F. Mendes, Filipe A. Almeida Paz, Maria A. F. Faustino, El Mostapha Rakib, M. Graça P. M. S. Neves, Florbela Pereira, Nuno M. M. Moura

**Affiliations:** † Laboratory of Molecular Chemistry, Materials and Catalysis, Faculty of Sciences and Technics, Sultan Moulay Slimane University, BP 523, Beni-Mellal 23000, Morocco; ‡ LAQV-REQUIMTE, Department of Chemistry, University of Aveiro, 3810-193 Aveiro, Portugal; § CICECO − Aveiro Institute of Materials, Department of Chemistry, University of Aveiro, 3810-193 Aveiro, Portugal; ∥ Higher School of Technology, Sultan Moulay Slimane University, BP 336, Fkih Ben Salah, Morocco; ⊥ LAQV-REQUIMTE, Department of Chemistry, NOVA School of Science and Technology, Universidade Nova de Lisboa, 2829-516 Caparica, Portugal

**Keywords:** *N*-heterocycles, thiazolo-indazoles, target prediction, single-crystal X-ray diffraction, molecular docking, acetylcholinesterase, Alzheimer’s disease

## Abstract

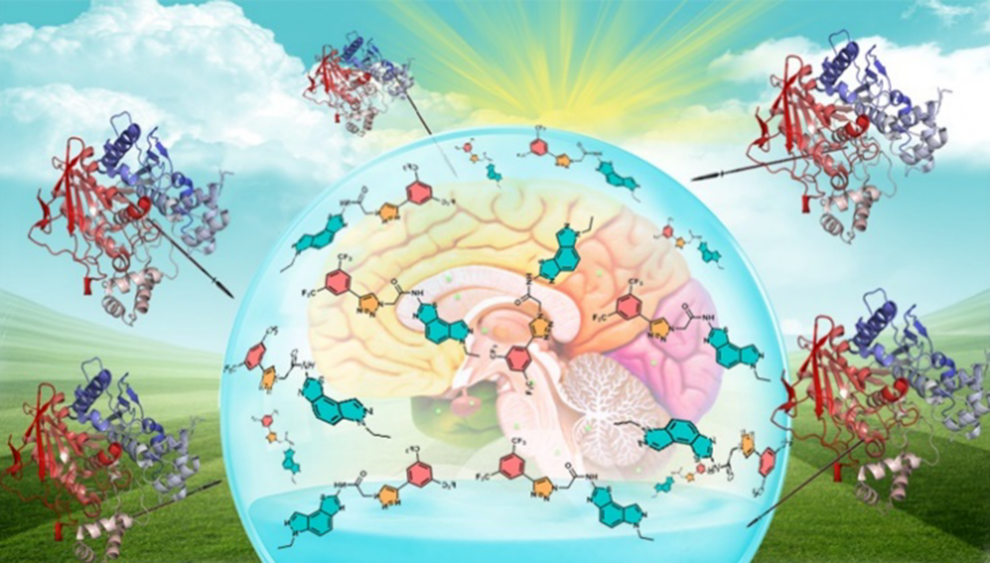

Alzheimer’s disease is characterized by a progressive deterioration of cognitive function and memory loss, and it is closely associated with the dysregulation of cholinergic neurotransmission. Since acetylcholinesterase (AChE) is a critical enzyme in the nervous system, responsible for breaking down the neurotransmitter acetylcholine, its inhibition holds a significant interest in the treatment of various neurological disorders. Therefore, it is crucial to develop efficient AChE inhibitors capable of increasing acetylcholine levels, ultimately leading to improved cholinergic neurotransmission. The results reported here represent a step forward in the development of novel thiazoloindazole-based compounds that have the potential to serve as effective AChE inhibitors. Molecular docking studies revealed that certain of the evaluated nitroindazole-based compounds outperformed donepezil, a well-known AChE inhibitor used in Alzheimer’s disease treatment. Sustained by these findings, two series of compounds were synthesized. One series included a triazole moiety (**Tl45a–c**), while the other incorporated a carbazole moiety (**Tl58a–c**). These compounds were isolated in yields ranging from 66 to 87% through nucleophilic substitution and Cu(I)-catalyzed azide–alkyne 1,3-dipolar cycloaddition (CuAAC) reactions. Among the synthesized compounds, the thiazoloindazole-based **6b** core derivatives emerged as selective AChE inhibitors, exhibiting remarkable IC_50_ values of less than 1.0 μM. Notably, derivative **Tl45b** displays superior performance as an AChE inhibitor, boasting the lowest IC_50_ (0.071 ± 0.014 μM). Structure–activity relationship (SAR) analysis indicated that derivatives containing the bis(trifluoromethyl)phenyl-triazolyl group demonstrated the most promising activity against AChE, when compared to more rigid substituents such as carbazolyl moiety. The combination of molecular docking and experimental synthesis provides a suitable and promising strategy for the development of new efficient thiazoloindazole-based AChE inhibitors.

## 1 Introduction

Acetylcholinesterase (AChE) plays a crucial role in the nervous system by breaking down the neurotransmitter acetylcholine (ACh) at cholinergic synapses.^1^ ACh is a vital neurotransmitter responsible for transmitting nerve impulses across synapses, thereby enabling communication between nerve cells and facilitating muscle contractions. These cholinergic synapses are widely distributed throughout both the central and peripheral nervous systems, which is essential for the proper functioning of various physiological processes in the human body.^1,2^ Once ACh has fulfilled its signaling function, AChE rapidly degrades it into inactive components, effectively terminating the nerve signal and allowing the system to reset for the next transmission.^3,4^

Alzheimer's disease is a chronic and progressive neurodegenerative condition often associated with damage to the cholinergic system. Patients with Alzheimer’s typically experience worsening memory loss, behavioral abnormalities, cognitive impairment, and a decline in judgment and abstract thinking.^5−8^ When it comes to managing degenerative diseases, the primary goals are to alleviate symptoms, slow down the progression of the condition, and enhance the patient’s overall quality of life. Despite notable advancements in research and treatment approaches, many degenerative diseases still lack curative therapies.^9−11^ Timely identification and early intervention, coupled with continuous medical investigation, continue to be critical in the quest for efficient therapies and potential remedies for these incapacitating disorders.^9,12,13^ Molecules capable of inhibiting AChE hold promise as potential medications for Alzheimer’s disease, as well as other neurodegenerative conditions like amyotrophic lateral sclerosis or Parkinson's disease.^14^

Molecules involved in the action of ChEs, also known anticholinesterases or indirect-acting cholinergic drugs, are recognized as pivotal candidates for the development of new medications aimed at addressing neurodegenerative diseases. When the AChE is inhibited, it loses its ability to hydrolyze the neurotransmitter ACh. Consequently, this neurotransmitter remains active in the synaptic cleft for an extended duration, resulting in elevated levels of ACh at the synapses.^15^ This enhanced cholinergic neurotransmission holds significant therapeutic potential, in the treatment of neurodegenerative conditions and neuromuscular disorders.

Tacrine was the first medication authorized by the FDA for Alzheimer’s disease treatment; however, it comes with several limitations. These include a short half-life, necessitating frequent dosing, a relatively high risk of liver toxicity, and peripheral cholinergic system stimulation, resulting in gastrointestinal discomfort like nausea, vomiting, abdominal pain, and diarrhea.^16^ Due to these drawbacks, safer and more effective AChE inhibitors (AChEIs), like donepezil, rivastigmine, and galantamine, have largely replaced tacrine in Alzheimer’s therapy. Furthermore, several other AChEIs are presently undergoing clinical trials for potential inclusion in future advancements in the field of Alzheimer’s disease treatment.^17^ In addition to the potency against AChE, the development of selective AChE inhibitors has always been one of the focuses for the development of novel treatments against Alzheimer’s disease, since the inhibition of butyrylcholinesterase (BuChE) can result in several adverse effects. These effects are related to the fact that BuChE, an enzyme also responsible for the hydrolysis of ACh, is more widely distributed through various tissues and its inhibition can lead to systemic adverse effects.^18,19^ Given the relatively modest effectiveness and noteworthy drawbacks presented by the existing AChE inhibitor drugs together with their reduced availability, the scientific community has been developing significant efforts to synthesize new molecules with enhanced AChE inhibitory activity.^20−22^ A commonly used approach involves the preparation of new molecules through the conjugation of two or more heterocyclic moieties, a method that has been gaining popularity among the scientific community.^23−25^

Within the array of available heterocycles, indazoles and thiazoles stand out due to their diverse and significant pharmacological properties, rendering them a great interest in the realms of medicinal chemistry and drug discovery. Compounds incorporating indazole units are being investigated as potential candidates for use in anti-inflammatory, antifungal, antimicrobial, kinase, and nitric oxide synthase (NOS) inhibitors, as well as anticancer agents, among other applications.^26−30^ Furthermore, the presence of thiazole moiety is considered relevant in the development of new drugs with potential anticancer, antibacterial, anti-inflammatory, analgesic, antitubercular, anti-Alzheimer, and antidiabetic properties.^31−36^ Synthetic methodologies leading to the fusion of thiazoles with other heterocycles like carbazoles, indoles, and indazoles have also been reported to yield novel thiazoloheteroarenes with bioactive potential across various fields.^37,38^

In our pursuit to develop new bioactive molecules based on indazole units,^39−41^ we report here an efficient one-pot synthesis of amino-thiazoloindazole with excellent yields. By performing Mondrian conformal target prediction with the ChEMBL database, we identified AChE as a particularly promising target for the activity of amino-thiazoloindazole derivatives. Based on the data obtained, these derivatives were employed as scaffolds to obtain novel compounds with enhanced activity as AChEIs through modifications to the thiazolo[5,4-*e*]indazole core. We conducted molecular docking studies to assess the potential of both starting scaffolds and a virtual compound library that underwent modifications at the C-2 position of the thiazoloindazole moiety. The most promising virtual derivatives were then synthesized, and their AChE inhibitory activities were assessed. Through this comprehensive approach, we aim to push the boundaries of bioactive molecules research and provide valuable contributions toward the development of novel and promising AChE inhibitors.

## 2 Results and Discussion

### 2.1 Synthesis

**Scheme 1:**
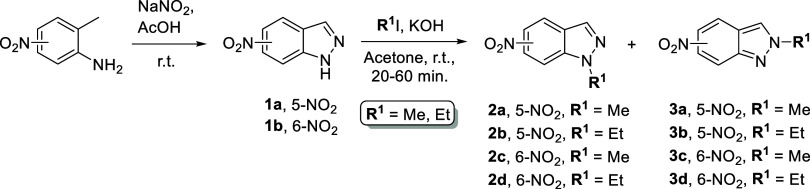
Synthesis and *N*-Alkylation of Nitroindazole **1a,b**(41)

**Scheme 2:**
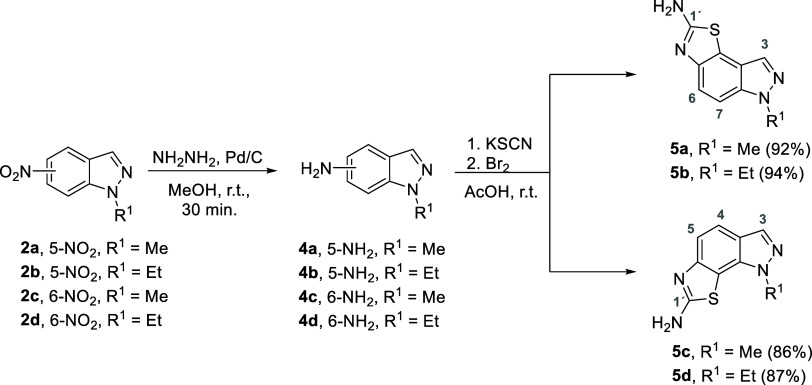
Synthetic Approach to Prepare Fused-amino-thiazoloindazole Scaffolds **5a**–**d**

**Scheme 3:**
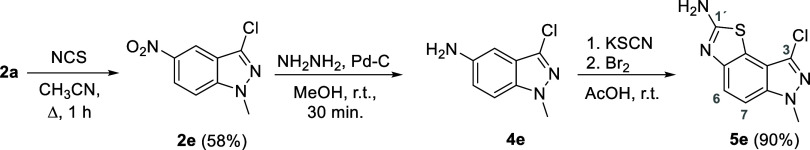
Synthesis of Thiazolo[5,4-*e*]indazol-2-amine **5e**

The fused thiazoloindazol-2-amine scaffolds **5a–e** were synthesized from the adequate 5-nitro- or 6-nitro-1*H*-indazole (**2a**-**2d**) according to the synthetic approaches outlined in Schemes 2 and 3. First, the starting nitroindazoles **1a**,**b** were obtained through diazotization of the appropriate nitro-aminotoluene in an acidic medium, according to the literature.^40,41^*N*-alkylation with methyl iodide or ethyl iodide in acetone at room temperature and in the presence of KOH yielded the expected *N*-1 and *N*-2 regioisomers, which were easily separated by column chromatography (Scheme 1).^41^

Then, each nitro indazole **2a**–**d** was reduced with hydrazine to form the corresponding amino-indazole intermediates **4a**–**d** in nearly quantitative yields. However, it must be noted that due to their unstable behavior, the amino-indazole intermediates were directly used in the subsequent step. The desired fused-amino thiazoloindazole scaffolds **5a**–**d** were then obtained by reacting the amino-indazole derivatives **4a**–**d** with potassium thiocyanate in glacial acetic acid under oxidative conditions in the presence of bromine, affording the *N*-alkylated thiazolo[5,4-*e*]indazol-2-amine compounds **5a**–**d** in excellent yields (86–94%) (Scheme 2).

It is worth referring that the preparation of 2-aminothiothiazolo[5,4-*e*]- and 2-aminothio[4,5-*g*]indazoles were already reported in the literature.^37^ However, the synthetic route here described allows efficient access to 2-aminothiazoloindazoles **5a**–**d** via a convenient one-pot approach with high yields (up to 94%). This method offers advantages over the previously reported multistep approaches, making it more efficient and practical. Another advantage of the methodology here discussed, when compared with those reported in the literature, is the synthesis of thiazoloindazoles modified with a primary amino functionality at C-2, whereas other methodologies only enable access to derivatives with secondary amino functionalities at the same position.

Once the indazole core is susceptible to halogenation at the C-3 position,^42,43^ we decided to evaluate the possibility of obtaining fused-amino-thiazoloindazoles from indazole derivatives substituted at C-3 with a halogen atom (Scheme 3). For this, the chlorination of **2a** with *N*-chlorosuccinimide (NCS) was performed affording the chlorinated indazole **2e** at C-3, in 58% yield. Then, the reaction of **2e** with potassium thiocyanate, under the conditions previously described afforded the corresponding thiazolo[5,4-*e*]indazol-2-amine **5e** in an excellent 90% yield.

The same reaction was performed with the brominated analogue, prepared from the reaction of **2a** with *N*-bromosuccinimide (NBS) but, regrettably, failed to afford the corresponding brominated thiazolo[5,4-*e*]indazol-2-amine. During this synthesis, we observed that, under all the studied conditions, the bromo substituent acted as a leaving group, being replaced by a hydrogen atom and, consequently, yielding the nonsubstituted derivative **5a**, with yields analogous to the previous one reported.

**Scheme 4:**
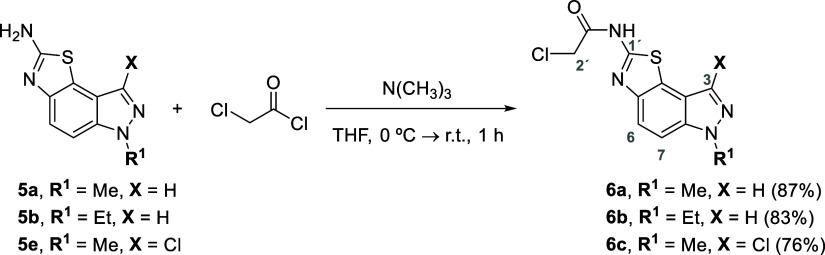
Preparation of the Thiazolo[5,4-*e*]indazol-2-acetamides **6a**–**c** from the Corresponding Amines **5**

Further functionalization of compounds **5a,b** and **5e** with chloroacetyl chloride afforded the desired functional acetamide derivatives **6** (Scheme 4), which are able to be used as building blocks for further reactions to modify the fused-thiazolo[5,4-*e*]indazole moiety and fine-tune the biological properties of these compounds. These nucleophilic acyl substitution reactions involving the thiazolo[5,4-*e*]indazol-2-amines **5a,b** and **5e** and chloroacetyl chloride were performed in THF at room temperature for 1 h and allowed to isolate the acetamide derivatives **6a**–**c** in very good yields (76–87%) after a simple precipitation in water (Scheme 4).

**Scheme 5:**
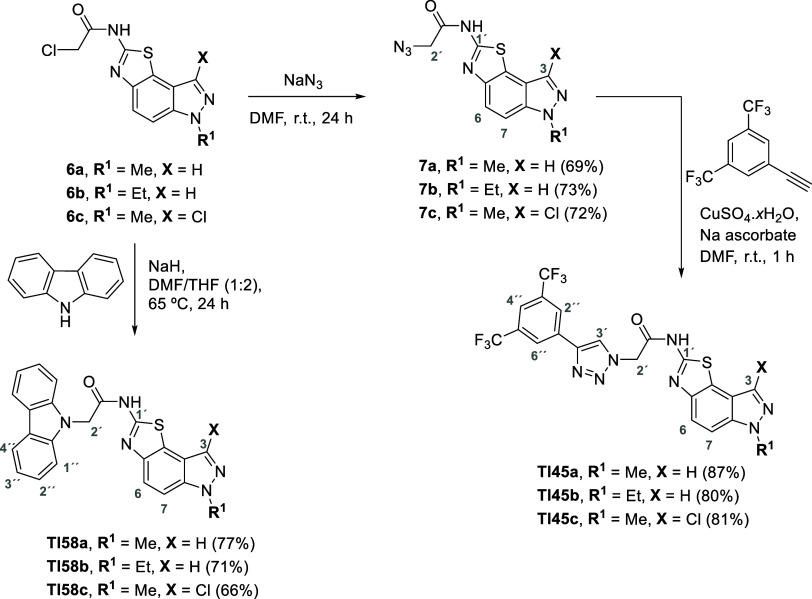
Synthesis of Triazolo-thiazolo[5,4-*e*]indazole Derivatives **Tl45a–c** and Carbazolo-thiazolo[5,4-*e*]indazole Derivatives **Tl58a–c**

Keeping in mind the main goal of this work, which is to develop new thiazolo[5,4-*e*]indazol-based derivatives able to act as efficient AChE inhibitors and after evaluating a virtual library based on those derivatives through computational studies (vide infra section 2.4), we decided to synthesize compounds **Tl45a–c** and **Tl58a–c** bearing triazole and carbazole moieties, respectively (Scheme 5). The rationale behind these choices involved three key aspects: high activity as AChE inhibitors predicted by molecular docking studies, easily accessible and low-cost reagents, and simple synthetic approaches with the potential for high yields, broad applicability, and a reduced probability of producing byproducts.

The first synthetic approach selected was the Cu(I)-catalyzed azide–alkyne cycloaddition (CuAAC) reaction to afford derivatives **Tl45a–c** (Scheme 5). This methodology allowed the attachment of an additional biologically active motif, the 1,2,3-triazole ring, to the thiazolo[5,4-*e*]indazol core. The CuAAC is the most successful approach to prepare 1,2,3-triazoles displaying great advantages when compared with other synthetic approaches, like wide scope, high pH tolerance, in situ generation of Cu(I) ions, water compatibility (as solvent), lower reaction time, temperature and reagent amounts, improved reaction kinetics and efficiency, and high regioselectivity.^44−46^ Since the synthetic developments introduced by Sharpless and Meldal, 1,2,3-trizole chemistry has been successfully explored to afford compounds that have been widely used as surrogates for a broad range of applications, namely for medicinal purposes due to their antitumoral, antibacterial, antifungal, antiparasitic, antiviral, anti-inflammatory, and Alzheimer’s disease inhibitors features.^46^

The synthesis of triazole-thiazolo[5,4-*e*]indazol derivatives **Tl45a–c** involved the previous preparation of the azido-thiazolo[5,4-*e*]indazol derivatives **7a**–**c** by reaction of the appropriate derivative **6a**–**c** with sodium azide in DMF. This approach afforded the expected azido derivatives in yields of 69–73%. Afterward, azido-thiazolo[5,4-*e*]indazol derivatives **7a**–**c** were used in the Cu(I)-promoted AAC reaction with 1-ethynyl-3,5-bis(trifluoromethyl)benzene as the diaporophile. These reactions involved the addition of the terminal alkyne to a solution of the adequate azido derivative **7a**–**c** in DMF in the presence of sodium ascorbate and 5 mol % CuSO_4_. The reaction was carried out at room temperature for 1 h, until full consumption of the starting material, and afforded the triazolo-thiazolo[5,4-*e*]indazole derivatives **Tl45a–c** with yields ranging from 80 to 87%.

Motivated by predictions attained from molecular docking studies, a carbazole synthon was also bound to the thiazolo[5,4-*e*]indazole core. The nucleophilic substitution of the chloro substituent at the acetamide moiety of the appropriate thiazolo[5,4-*e*]indazole derivatives **6a**–**c** with carbazole was performed in the presence of NaH in a DMF/THF mixture at 65 °C. After 24 h of reaction, all the starting material was consumed with the formation of a main compound. After workup and purification by column chromatography carbazolo-thiazolo[5,4-*e*]indazole derivatives **Tl58a–c** were readily achieved in very good yields (66–77%).

The yield of nucleophilic substitution and CuAAC reactions are slightly affected when both reactions were performed with the adequate *N*-ethyl alkylated derivative **6b** or **7b**, as well as when using the chloro-substituted *N*-methyl derivatives **6c** or **7c**. The reactions performed with the *N*-methyl derivatives **6a** or **7a**, allowed a yield enhancement of up to 6% and 11% for the products from the CuAAC and nucleophilic substitution, respectively.

### 2.2 Structural Characterization

The structures of all the synthesized compounds were unequivocally confirmed using NMR spectroscopy (Figures S1–S37) and mass spectrometry (Figures S38–S71). Furthermore, the structures of compounds **5d**, **6b**, **Tl45c**, **TI58a**, and **TI58c** were elucidated and confirmed from single-crystal X-ray diffraction studies (see Section 2.3).

The ^1^H NMR spectra of compounds **5a**–**d** exhibited a characteristic pattern in the aromatic region. This included a singlet around δ 8.2 ppm, arising from the resonance of the H-3 proton in the indazole moiety, and two doublets ranging from δ 7.77 ppm to δ 7.29 ppm, ascribed to the resonances of the six-membered ring protons in the indazole unit. It is worth highlighting that compound **5b** showed signals generated by the resonances of H-3 and H-7 as a doublet and doublet of doublets with a small coupling constant (*J* = 0.9 Hz) due to long-range inter-ring “zig-zag” coupling. The ^1^H NMR spectrum of compound **5e**, on the other hand, did not exhibit the signal from the resonance of the H-3 proton, endorsing the substitution of the hydrogen by a chloro atom. In the ^13^C NMR spectra of this series of thiazolo[5,4-*e*]indazol-amine derivatives, the most deshielded signal was attributed to the resonance of the C-2 carbon at the thiazolo-fused ring.

Concerning the ^1^H NMR of the thiazolo[5,4-*e*]indazol-2-yl)acetamide derivatives **6a**–**c**, new singlets appeared in the range δ 4.5–4.1 ppm, arising from the resonance of the CH_2_ protons of the 2-chloroacetamide unit. Additionally, a singlet at around δ 12.8 ppm was observed in the ^1^H NMR spectra of this series of compounds, corresponding to the resonance of the *N*-H proton from the same unit. These two signals supported the success of the reaction between the amino-thiazolo[5,4-*e*]indazole derivatives **5** and 2-chloroacetyl chloride. The formation of the thiazolo[5,4-*e*]indazol-2-yl)acetamide derivatives **6a**–**c** induced the appearance of two new signals at around δ 155.0 ppm and δ 42.8 ppm in the ^13^C NMR spectra, corresponding to the resonance of the carbonyl and CH_2_ carbons, respectively, from the acetamide moiety.

The formation of azide derivatives **7a**–**c** did not induce noticeable changes in the chemical shifts of the signals generated by the resonances of the aromatic protons when compared with the ones of the corresponding precursors **6a**–**c**. However, in the aliphatic region, a shield effect of ca. 0.7 ppm was observed for the singlet generated by the resonance of the CH_2_ protons when compared with the analogue protons in **6a**–**c** (ca. δ 3.8 ppm versus δ 4.5 ppm). The formation of the triazole derivatives **Tl45a–c** was confirmed by ^1^H NMR due to the presence of characteristic singlets in the aromatic region, ascribed to the resonance of the CH proton from the triazole ring at around δ 8.1 ppm and from the 3,5-bis(trifluoromethyl)phenyl at ca. δ 9.0 ppm and δ 8.6 ppm.

The linkage of a carbazole unit to the thiazolo[5,4-*e*]indazole induced changes in the ^1^H NMR signals of the **Tl58a-c** derivatives, including a significant shielding effect (≈ 0.2 ppm) in the signals generated by the resonances of H-3, H-6, and H-7 protons, as well as higher molecular asymmetry. The most deshielded signal in the ^1^H NMR was a duplet (δ ≈ 8.18 ppm), assigned to the resonance of the protons at the 4,5-positions of the carbazole unit, while the 1,8-positions protons appear as a multiplet ranging from δ 7.56 ppm to δ 7.48 ppm.

The structure and molecular formula of all the synthesized compounds were confirmed by their (HR)MS-ESI(+) spectra, which displayed the presence of the peaks corresponding to the [M + H]^+^ molecular ion.

### 2.3 Single-Crystal X-Ray Diffraction

**Figure 1:**
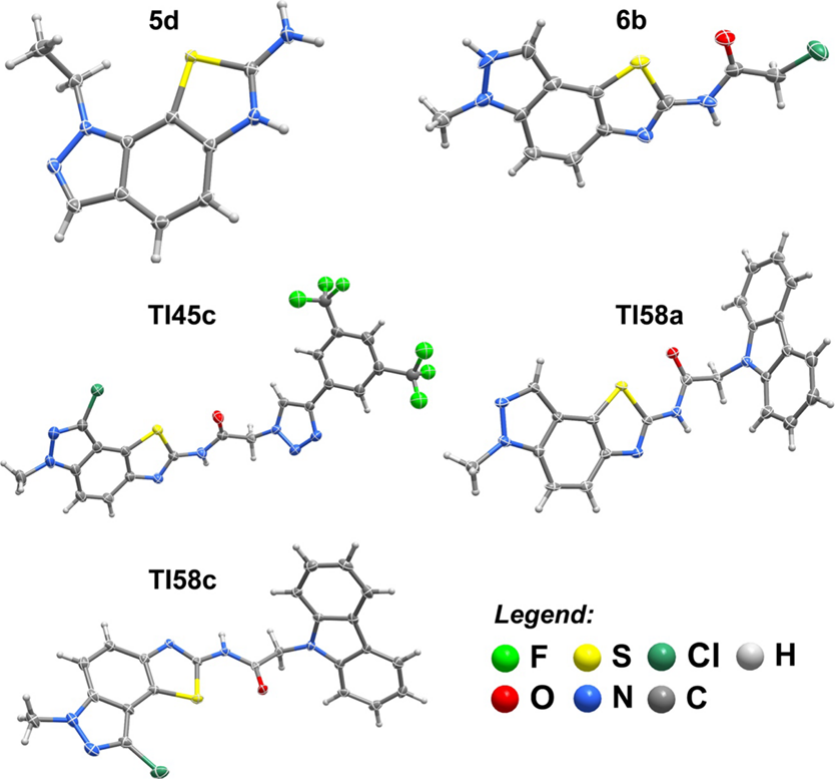
Schematic representation of the most representative molecular units present in the crystal structures of compounds **5d**, **6b**, **TI45c**, **TI58a**, and **TI58c**. Non-hydrogen atoms are represented as thermal ellipsoids drawn at the 30% probability level and hydrogen atoms are depicted as small spheres with arbitrary radii.

The structural features of five thiazoloindazole-based derivatives were unveiled from single-crystal X-ray diffraction (Figure 1). Compound **5d** crystallizes in the centrosymmetric triclinic space group *P*-1 with the asymmetric unit composed of a positively charged 1-ethyl-1*H*-thiazolo[4,5-*g*]indazol-7-amine molecule (because of the protonation of the nitrogen atom N2), whose charge is balanced by a thiocyanate anion. The crystal packing is achieved solely by hydrogen interactions between the amino group and the adjacent nitrogen from the indazole ring [*d*_N···N_ found 2.7794(17) Å with < (NHN) interaction angle of 158° – *not shown*] and several π–π stacking interactions between the stacked indazole backbones [*d*_π···π_ = 3.4541(11)–3.8021(11) Å)] (Figure S72a). Compound **6b** crystallizes in the orthorhombic *Aba*2 space group, with the asymmetric unit comprising three 2-chloro-*N*-(6-ethyl-6*H*-thiazolo[5,4-*e*]indazol-2-yl)acetamide molecules. The high number of donor–acceptor atoms leads to a close packing achieved only by strong hydrogen bonding interactions between the indazole rings and the adjacent acetamide residues [*d*_N···N_ found in the 2.896(14)–2.923(13) Å range with < (NHN) interaction angles between 157° and 163°, and *d*_N···O_ of 2.819(11) Å with a < (NHO) interaction angle of 165° (dashed orange lines in Figure S72b].

The 1,3-dippolar cycloaddition of compound **7c** with 1-ethynyl-3,5-bis(trifluoromethyl)benzene to form **TI45c** was corroborated by X-ray diffraction analysis. Compound **TI45c** crystallizes in the noncentrosymmetric *P*2_1_2_1_2_1_ orthorhombic space group, with the asymmetric unit comprising a whole molecular unit and two DMSO solvent molecules. Despite the high number of donor and acceptor atoms, the number of hydrogen bonding interactions is small and of weak nature, mainly of the C–H···O and C–H···F type, with typical geometrical parameters between molecular residues, and some strong N–H···O interactions with the DMSO solvent molecules. In fact, the weak nature of these interactions is at the genesis of the high disorder present in these DMSO solvent molecules and of the two −CF_3_ groups. The crystal packing is achieved mostly by strong π–π interactions between the stacked indazole backbones and between the aromatic ring and an adjacent triazole residue [*d*_π···π_ = 3.700(5)–3.872(4) Å)] (Figure S72c).

Finally, the carbazole-thiazolo[5,4-*e*]indazole derivatives **TI58a** and **TI58c** crystallize in the orthorhombic *P*bca and in the triclinic *P*-1 space groups, respectively. The asymmetric unit of **TI58a** is composed of two molecular units with two crystallization ethanol molecules (one ethanol molecule is disordered over two crystallographic sites, with 57 and 43% occupancy rates). The crystal packing is achieved by strong N–H···O hydrogen bonding interactions between the ethanol molecules and the nitrogen from the adjacent indazole and acetamide residues [*d*_N···O_ found in the 2.802(5)–2.88 (13) Å range with < (NHO) interaction angles between 164° and 173°]. The crystal packing of **TI58a** is quite dense with no structural evidence of π–π interactions. In a similar way, the asymmetric unit of **TI58c** is composed only of two molecular units, without any crystallization solvent present. While not as dense as for the previous compound, the crystal packing of **TI58c** is solely achieved by weak hydrogen bonding interactions (mainly of the C–H···C kind) and two N–H···O interactions between acetamide residues of adjacent molecules [*d*_N···O_ found in the 2.890(4)–2.933(4) Å range with < (NHO) interaction angles between 170° and 172°] (Figure S72d).

### 2.4 Computational Studies

**Figure 2:**
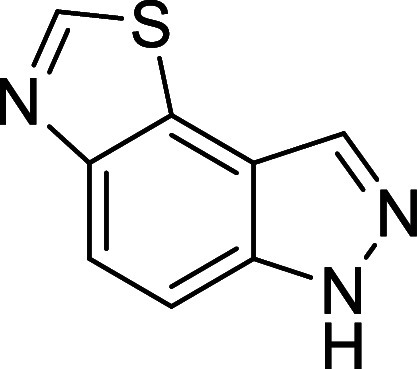
Chemical structure of the thiazolo[5,4-*e*]indazole-template.

The human targets prediction was performed considering the Mondrian conformal prediction (MCP)^47^ using the ChEMBL database,^48^ which comprises data from 550 human protein targets with different bioactivity profiles. In total, the ChEMBL database used (release 24) contains a total of >15 million bioactivity measurements for 1.8 million distinct compounds.^48^ The prediction for the template (Figure 2) was performed and it was identified as having a high probability of being active against AChE enzyme. The Tanimoto coefficient (TC) of similarity between the template and each one of the synthesized thiazoloindazole derivatives (**5a**–**e**, and **6a**–**c**) was calculated and TC values for all derivatives are in the range between 0.42 and 0.70.

**Figure 3:**
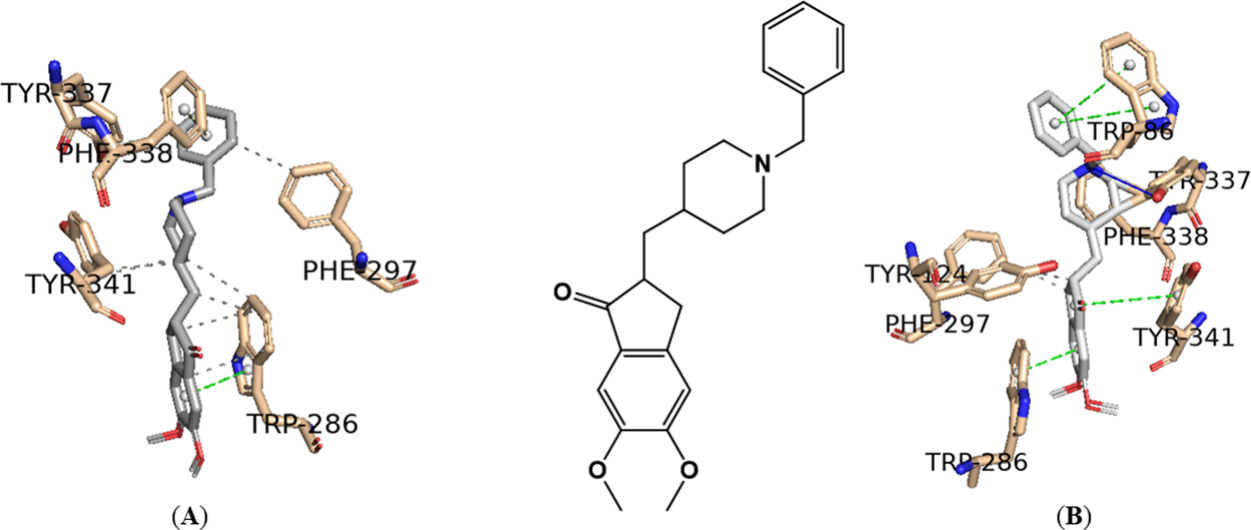
Interaction profile of the best-docked pose for the positive control, donepezil, against AChE enzyme using the (A) AutoDock Vina and (B) DockThor software. Hydrophobic interactions are shown as black dashed lines, while π-stacking interactions are depicted as green (parallel) and gray (perpendicular) dashed lines.

Molecular docking was applied to elucidate the binding action of 189 thiazoloindazole-based derivatives against AChE enzyme (PDB ID 4EY4):^49^ five thiazolo[4,5-*e*]indazol-2-amines derivatives synthesized (**5a**–**e**), six synthesized precursors (**6a**–**c** and **7a**–**c**) and 178 virtual derivatives, one thiazoloindazole template (Figure 2), and 59 derivatives for each one of the **TIXa**, **TIXb**, and **TIXc** (**X** = 1–59) cores (Figure S73). In Table S1 of the Supplementary Data, the results of molecular docking using the AutoDock Vina software against the AChE enzyme were represented and summarized. DockThor, a web service for molecular docking simulation (https://dockthor.lncc.br/v2/), was also utilized to perform molecular docking of the three most promising thiazoloindazole-based derivatives (**Tl45c**, **Tl45a**, and **Tl45b**, Table 1) and the positive control (donepezil) against the AChE enzyme (PDB ID 4EY4).^50^ In DockThor, a set of new empirical scoring functions to estimate protein–ligand binding affinity was developed by explicitly accounting for physics-based interaction terms based on the MMFF94S force field combined with machine learning.^50^ As shown in Figure 3, the best-docked pose for the positive control (donepezil) on the AChE enzyme was obtained using the AutoDock Vina and DockThor software.

**Table 1:**
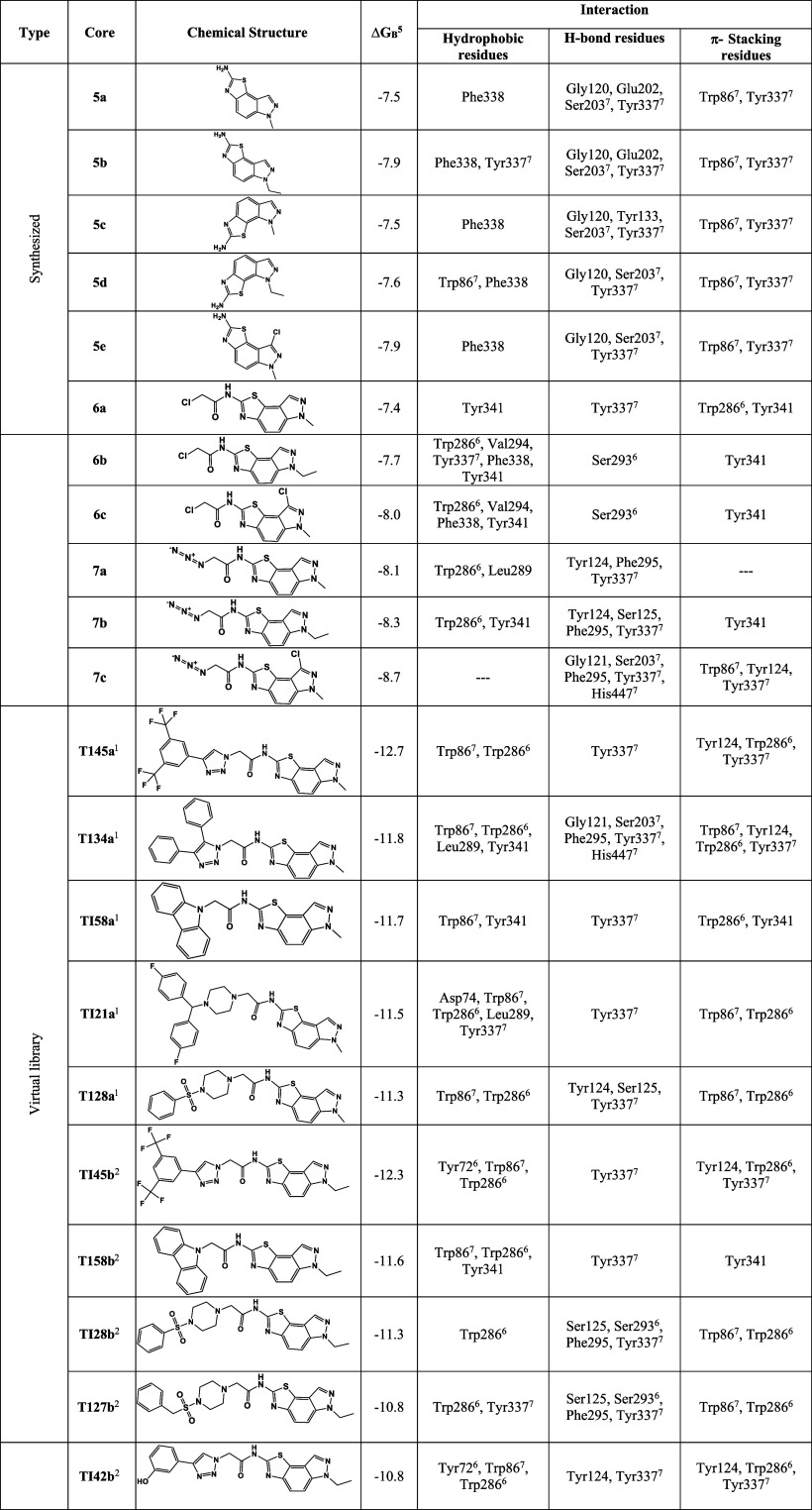

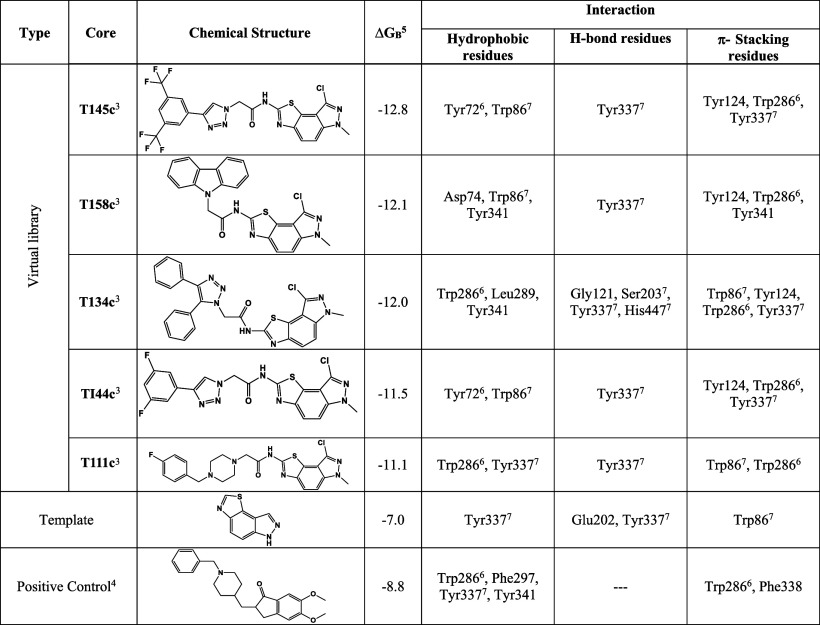
Calculated Free Binding Energies (Δ*G*_B_, in kcal/mol) and the Detailed Interactions Established upon Docking the Selected 27 Thiazoloindazole-Based Derivatives and the Positive Control, Donepezil, against AChE Enzyme

1 The top 5 virtual derivatives for the **TIXa** core.

2 The top 5 virtual derivatives for the **TIXb** core.

3 The top 5 virtual derivatives for the **TIXc** core.

4 Donepezil, an AChE inhibitor used for Alzheimer's disease therapy.

5 In kcal/mol.

6 PAS amino acid residues.

7 CAS amino acid residues.

The human AChE active site is a long gorge with a total length of approximately 20 Å^51^ (Figure 3), consisting mainly of a catalytic active (or anionic) site (CAS) at the bottom of the gorge (His447, Ser203, Trp86, Tyr337), while the peripheral anionic site (PAS) is situated near the entrance of gorge (His287, Ser293, Trp286, Tyr 72). These two are connected by a narrow groove (Tyr124, Phe295, Tyr341). Compounds that can interact with CAS and PAS are desirable as they are thought to exert multiple therapeutic effects,^51,52^ as can be seen with donepezil in Figure 3 and Table 1. Despite the best pose for the positive control against the AChE enzyme using AutoDock Vina (A) not showing the classic interaction with the CAS residue Trp86,^53^ as this residue is more than 4 Å away, specifically 5.53 Å, this interaction is present in the best pose of the positive control against the AChE enzyme using DockThor (B) (Figure 3). To validate the redocking processes, the PyMOL software was used to superimpose the docked complexes (A) and (B) of the positive control donepezil against AChE (Figure 3) onto the solved structure (PDB ID 4EY7). The root-mean-square displacement (RMSD) between complex (A) and the solved structure was 0.657 Å, and between complex (B) and the solved structure was 0.551 Å, indicating high structural similarities in both cases.

A flexible molecular docking using AutoDock Vina was conducted to perform the virtual screening of the 189 thiazoloindazole-based derivatives to find the most favorable binding interactions, and the calculated free binding energies by the set of search space coordinates, which are reported in Table 1 for the top five virtual derivatives selected for each core, five thiazolo[4,5-*e*]indazol-2-amines derivatives synthesized (**5a**–**e**), the six synthesized precursors (**6a**–**c** and **7a**–**c**), the thiazoloindazole-template, and the positive control (donepezil).

**Figure 4:**
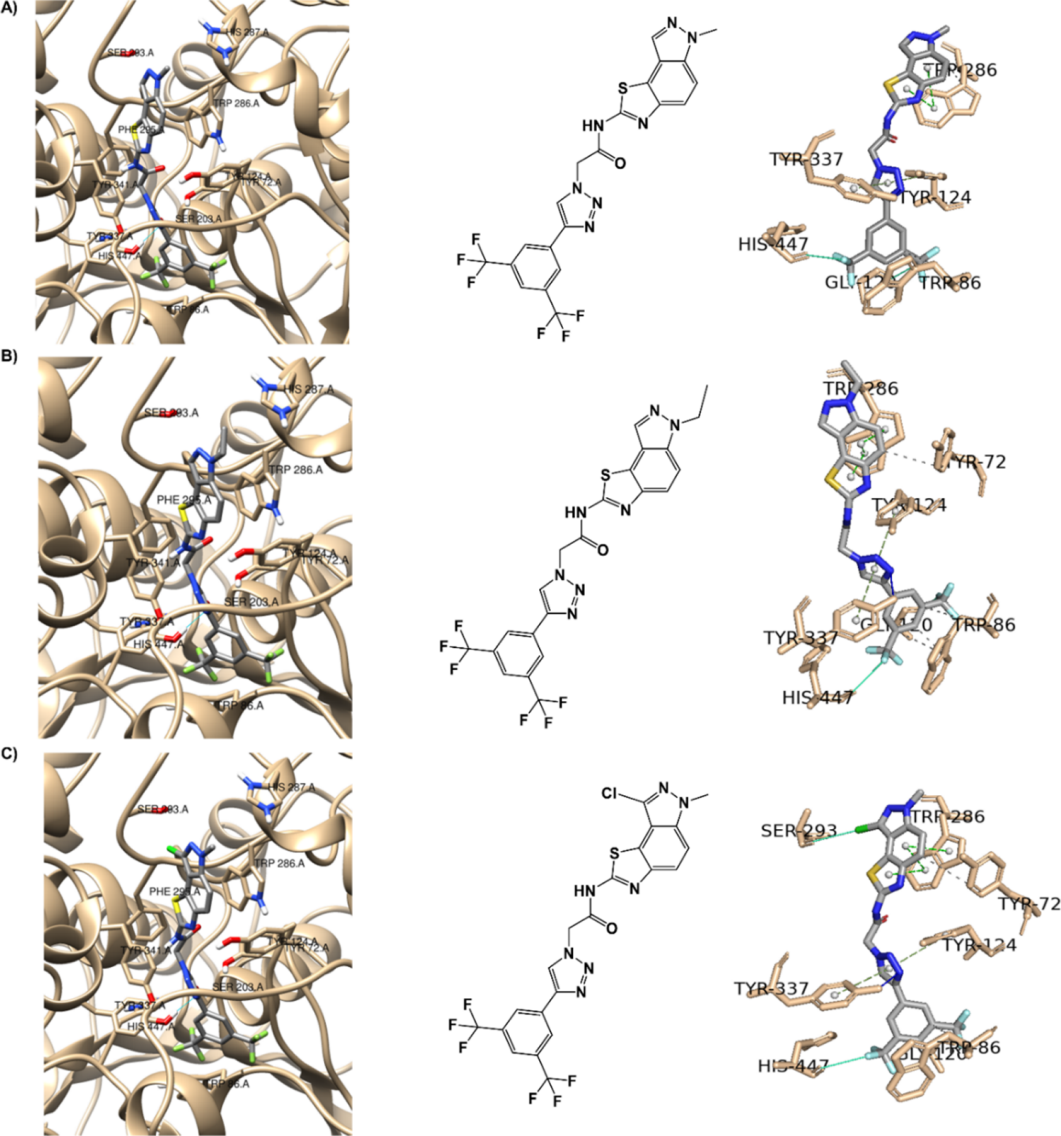
Interaction profiles of the best-docked poses for the (A) **TI45a**, (B) **TI45b** and (C) **TI45c**. The hydrophobic interactions are shown as black dash lines and the π-stacking interactions in green (parallel) and gray (perpendicular) dash lines. H-bond and halogen-bond interactions are shown as blue and green continuous lines, respectively.

As can be seen in Table 1, the derivatives with the lowest Δ*G*_B_ calculated, i.e., the most promising thiazoloindazole-based derivatives, are **TI45c** (**TIXc** core and bis(trifluoromethyl)phenyl-triazolyl-substituted), **Tl45a** (**TIXa** core and bis(trifluoromethyl)phenyl-triazolyl-substituted), **TI45b** (**TIXb** core and bis(trifluoromethyl)phenyl-triazolyl-substituted), **TI58c** (**TIXc** core and carbazolyl-substituted), and **TI34c** (**TIXc** core and diphenyl-triazolyl-substituted) with Δ*G*_B_ less than or equal to −12 kcal/mol, more precisely with values of −12.8, −12.7, −12.3, −12.1, and −12.0 kcal/mol, respectively (Table 1). Indeed, from those, three derivatives have the same **TIXc** core i.e. with chlorine functionality at position 3 of the indazole ring (**TI45c**, **TI58c**, and **TI34c**) and three derivatives have the same substituent, the bis(trifluoromethyl)phenyl-triazolyl moiety (**TI45a–c**). These excellent binding affinities could be attributed to potential hydrogen bond interactions with residue Tyr337 in the CAS of the AChE enzyme. Also, it is worth mentioning that the positive control (donepezil), a known AChE inhibitor used for Alzheimer's disease therapy, has a Δ*G*_B_ value calculated of −8.8 kcal/mol. In Figure 4, the best-docked poses for the three most probable lead-like anti-Alzheimer’s AChE inhibitors, **TI45a**, **TI45b** and **TI45c**, were shown. For example, in the three derivatives with the best substituent (bis(trifluoromethyl)phenyl-triazolyl) for each of the cores (**TI45a**, **TI45b** and **TI45c**), there appears to be a hydrogen bond interaction between the nitrogen atom at position 3 of the triazole ring and the hydroxyl group of residue Tyr337 for all derivatives, only varying the length of the hydrogen bond, namely, 2.91, 2.86, and 2.81 Å, respectively (Figure 4).

The DockThor scores for the three most promising thiazoloindazole-based derivatives (**TI45a**, **TI45b**, and **TI45c**, Table 1) and the positive control (donepezil) were −10.712 kcal/mol (−12.7 kcal/mol using Autodock Vina), −10.938 kcal/mol (−12.3 kcal/mol using Autodock Vina), −10.831 kcal/mol (−12.8 kcal/mol using Autodock Vina), and −10.983 kcal/mol (−8.8 kcal/mol using Autodock Vina), respectively.

### 2.5 AChE/BuChE Inhibitory Activity

The most promising virtual derivatives (**TI45a–c** and **TI58a–c**) that were predicted by molecular docking as the most probable lead-like anti-Alzheimer’s AChE inhibitors were synthesized and their AChE and BuChE inhibitory activities were evaluated against *Electrophorus electricus* AChE (eeAChE) and equine serum BuChE following an adaptation of the Ellman’s method,^54^Table 2. As was mentioned above, it is worth to refer that the readily available and low-cost eeAChE is an excellent model of the human enzyme hAChE since displays a considerable sequence identity (>85%) with it, and both enzymes present fully overlapping binding pockets.^55^ Although, the 11 thiazoloindazole-based precursors (**5a**–**e**, **6a**–**c**, and **7a**–**c**) that were already synthesized are less likely to be active against the AChE enzyme (Table 1) are interesting for a structure–activity relationship (SAR) analysis and thus were also evaluated. The obtained results from these in vitro assays are represented in Table 2, with donepezil as the positive control.

**Table 2:** Cholinesterase Inhibitory Activity of Selected Thiazoloindazole-Based Derivatives

compound	AChE IC_50_(μM)	BuChE IC_50_(μM)
**5a**	>50	>50
**5b**	>50	>50
**5c**	>50	>50
**5d**	>50	>50
**5e**	>50	>50
**6a**	14.31 ± 2.62	>50
**6b**	13.16 ± 2.71	>50
**6c**	>25	>50
**7a**	>50	>50
**7b**	0.54 ± 0.11	>50
**7c**	2.26 ± 0.09	>50
**TI45a**	>50	>50
**TI45b**	0.071 ± 0.014	>50
**TI45c**	>50	>50
**TI58a**	>50	>50
**TI58b**	>50	>50
**TI58c**	>50	>50
**donepezil**	0.024 ± 0.006	4.72 ± 0.95

From the results depicted in Table 2, the potential of thiazoloindazole-based derivatives with a **6b** core as selective AChE inhibitors appears to be clear (**TI45b**, **7b**, and **6b**), with two of the **6b**-based core evaluated derivatives presenting IC_50_ values below 1 μM against AChE, corroborating to some extent the theoretical studies (Table 1).

### 2.6 Structure–Activity Relationship (SAR) Studies

**Figure 5:**
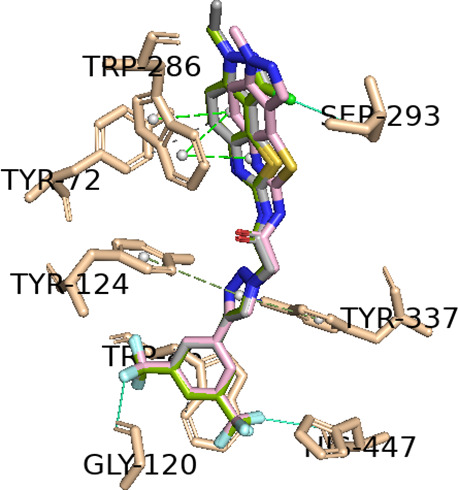
Interaction profiles of the best-docked poses for the **TI45a** (pink), **TI45b** (gray) and **TI45c** (green). The hydrophobic interactions are shown as black dash lines and the π-stacking interactions in green (parallel) and gray (perpendicular) dashed lines. H-bond and halogen-bond interactions are shown as blue and green continuous lines, respectively.

This work's computational and synthetic strategy allowed the clear identification of thiazoloindazole-based derivatives with promising activity against AChE, namely the **TI45b** derivative (Tables 1 and 2). However, considering the calculation of free binding energies by molecular docking for the three cores (**TI45a–c**) with the bis(trifluoromethyl)phenyl-triazolyl substituent of −12.7, −12.3, and −12.8 kcal/mol, respectively, there does not seem to be justification for the difference observed experimentally with the IC_50_ of >50, 0.071, and >50 μM, respectively (Tables 1 and 2). The redocking experiments of the three most promising thiazoloindazole-based derivatives (**TI45c**, **TI45a**, and **TI45b**, Table 1) and the positive control (donepezil) against the AChE enzyme using DockThor were conducted to explain the observed differences in experimental activities. However, despite the **TI45b** derivative being predicted to have the lowest binding energy (−10.938 kcal/mol) of the three derivatives, the differences in the estimated values do not appear to justify the experimentally obtained values. A possible explanation for this behavior may be related to the preferential interaction in the PAS of the AChE enzyme, namely, with the number of PAS residues. A detailed analysis of the interactions for these three derivatives is shown in Table 1. It is verified that only the **TI45b** derivative presents hydrophobic interactions with two PAS residues (Tyr72 and Trp286), while the other derivatives only have interactions with only one PAS residue, namely, Trp286 for the derived **TI45a** and Tyr72 for the derived **TI45c**. However, donepezil only interacts with only one PAS residue (Trp286) as the **TI45a** derivative, Table 1. As shown in Figure 5, the best-docked poses for the three derivatives, **TI45a–c**, were shown. The presence of the ethyl group at position 1 of the thiazoloindazole ring in the **TI45b** derivative instead of the methyl group, in the **TI45a** and **TI45c** derivatives, appears to be essential for activity against AChE, as it appears to allow hydrophobic interactions with the phenolic side chain of Tyr72 residue.

**Figure 6:**
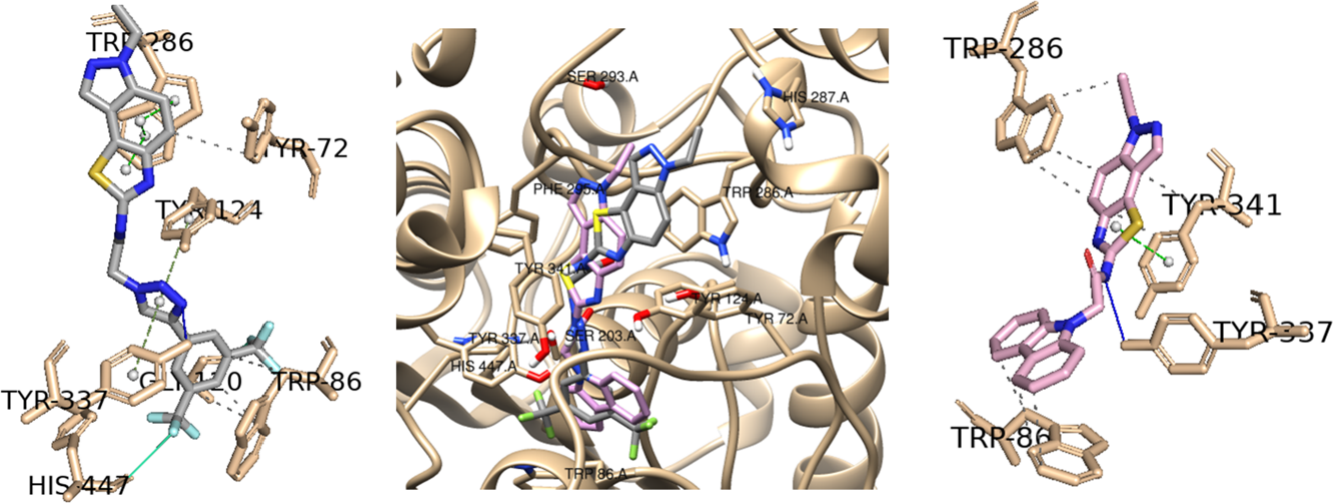
Interaction profiles of the best-docked poses for the **TI45b** (gray) and **TI58b** (pink). The hydrophobic interactions are shown as black dash lines and the π-stacking interactions in green (parallel) and gray (perpendicular) dash lines. H-bond and halogen-bond interactions are shown as blue and green continuous lines, respectively.

However, despite this preference for the **TlXb** core, the **TI58b** derivative with the carbazolyl substituent did not experimentally show any activity against AChE, Table 2. A possible justification can again be related to interactions with PAS residues. If we analyze the PAS interactions of the **TI58b** derivative in Table 1, we can see that it only presents interactions with one PAS residue, Trp286. Moreover, it has a lower number of interactions with CAS residues (two: Tyr337 and Trp86) than the **TI45b** derivative (three: Tyr337, Trp86, and His447). Figure 6 shows the best-docked poses for the two derivatives, **TI45b** and **TI58b**.

Therefore, it appears that more planar and less flexible substituents, such as the carbazoyl moiety in thiazoloindazole-based derivatives reduce activity against AChE when compared to more flexible substituents, such as bis(trifluoromethyl)phenyl-triazolyl.

In the series of synthesized precursors derivatives (**5a**–**e**, **6a**–**c**, and **7a**–**c**) the introduction in the 2-position of the thiazoloindazole core of a moderately activating electron-donating substituent such as amide group, in the derivatives **6a**–**c**, seems to favor activity against AChE when compared to strongly activating electron-donating substituent such as amine group, in the derivatives **5a**–**c**, Tables 1 and 2.

## 3 Materials and Methods

### 3.1 General

Nuclear magnetic resonance (NMR) spectra were recorded by using a Bruker AC 300 (^1^H) or 75 MHz (^13^C) instruments in deuterated chloroform (CDCl_3_) or dimethyl sulfoxide (DMSO-*d*_6_) and using tetramethylsilane (TMS) as the internal reference. Chemical shifts are given in δ parts per million (ppm) downfield from TMS; the coupling constants (*J*) in Hertz (Hz). Electrospray ionization mass spectra were acquired with a Micromass Q-Tof 2 (Micromass, Manchester, UK), operating in positive ion mode, equipped with a Z-spray source, an electrospray probe, and a syringe pump. The source and desolvation temperatures were 80 and 150 °C, respectively. The capillary voltage was 3000 V. The spectra were acquired at a nominal resolution of 9000 and at cone voltages of 30 V. Nebulisation and collision gases were N_2_ and Ar, respectively. Compound solutions in methanol were introduced at a 10 μL min^–1^ flow rate. Büchi-Tottoli apparatus was used to measure melting points.

Column chromatography was carried out on SiO_2_ (silica gel 60 Merck 0.063–0.200 mm). Thin-layer chromatography (TLC) was carried out on SiO_2_ (silica gel 60, F 254 Merck 0.063–0.200 mm), and chromatograms were visualized by UV at 254 and 365 nm. All reagents were of analytical grade and were used as received without further purification. Solvents were dried according to the literature procedures.^56^

### 3.2 Synthesis

#### 3.2.1 Preparation of *N*-Alkylated Nitroindazole Scaffolds **2a–d**

The *N*-alkylated nitro-indazoles were synthesized as described in the literature.^40,41^ Their purity was confirmed by thin layer chromatography (TLC) and by ^1^H NMR spectroscopy. Succinctly, diazotization of the adequate 5-nitro and 6-nitro-aminotoluene in acidic medium afforded the corresponding nitro-1*H*-indazoles **1a,b**. Then, to each acetone solution (5.0 mL) of **1a,b** (0.1 g, 0.61 mmol) were added KOH (0.1 g, 1.84 mmol, 3 equiv) and the appropriate methyl or ethyl alkylating agent (0.67 mmol, 1.1 equiv). Each reaction mixture was stirred in the range of 20 to 60 min at room temperature until TLC monitoring confirmed the consumption of the starting material. The corresponding *N*-methyl- and *N*-ethyl-nitroindazoles **2a**–**d** and **3a**–**d** were obtained pure after purification by column chromatography (silica gel) using hexane:AcOEt (4:1) as the eluent.

#### 3.2.2 Synthesis of 3-Chloro-5-nitroindazole, **2e**

To an acetonitrile (20 mL) solution of 5-nitroindazole **2a** (1.0 g, 6.12 mmol) was added *N*-chlorosuccinimide (0.9 g, 1.1 equiv. 6.74 mmol). The mixture was refluxed for 1 h, until the completion of the reaction, as followed by TLC monitoring. Then, the reaction was washed with water (2 × 100 mL), extracted with ethyl acetate (3 × 20 mL), the organic layer was recovered, and the solvent evaporated under reduced pressure. The crude product was purified by column chromatography using hexane/ethyl acetate (80:20) as the eluent. Compound **2e** was obtained in 58% yield as a light-yellow solid.

##### 3.2.2.1 3-Chloro-1-methyl-5-nitro-1*H*-indazole, **2e**

Yield: 58%. M.p.: 198.0–199.0 °C, ^1^H NMR (300 MHz, CDCl_3_): δ 8.65 (1H, d, *J* = 2.1 Hz, H-4), 8.31 (1H, dd, *J* = 9.2 and 2.1 Hz, H-7), 7.44 (1H, d, *J* = 9.2 Hz, H-6), 4.10 (3H, s, *N*CH_3_) ppm. ^13^C NMR (75 MHz, CDCl_3_): δ 142.8 (C-7a), 142.6 (C-5), 135.8 (C-3), 122.5 (C-3a), 120.5 (C-6), 117.8 (C-4), 109.9 (C-7), 36.4 (CH_3_) ppm.

#### 3.2.3 Synthesis of Thiazolo[4,5-*e*]indazol-2-amines, **5a–e**

Hydrazine hydrate (10 mL, 0.31 mol) and palladium/carbon (0.25 mol %, 240 mg) were added to a solution of each *N*-alkylated 5- or 6-nitro-1*H*-indazole derivatives **2a**–**e** (1 g, 6.13 mmol) in methanol (25 mL). Each mixture was stirred at reflux for 30 min (TLC monitoring), and then, palladium/carbon was filtered off. The expected 5- and 6-aminoindazole derivatives **4a**–**e** were obtained by evaporation of the solvent. Such compounds were then treated with potassium thiocyanate (6.13 mmol) in glacial acetic acid, at room temperature, followed by the dropwise addition of a glacial acetic acid solution (5 mL) of bromine (1.2 equiv). After completion of the reaction as revealed by the disappearance of the starting materials on TLC analysis, the liquid solution was neutralized with a saturated NaOH aqueous solution to pH 7 to 7.5 and then cooled overnight in the refrigerator to allow the product to precipitate. Then, each amine **5a**–**e** was filtered, washed with cold water, and dried under a vacuum.

##### 3.2.3.1 6-Methyl-6*H*-thiazolo[5,4-*e*]indazol-2-amine, **5a**

Yield: 92%. M.p.: > 300 °C. ^1^H NMR (300 MHz, DMSO-*d*_6_): δ 8.28 (1H, s, H-3), 7.74 (1H, d, *J* = 9.0 Hz, H-7), 7.57 (1H, d, *J* = 9.0 Hz, H-6), 4.08 (3H, s, *N*CH_3_) ppm. ^13^C NMR (75 MHz, DMSO-*d*_6_): δ 167.8(C-1′), 137.9, 135.7 (C-3), 130.5(C-7), 117.2 (C-7a), 115.1, 114. Eight (C-3a), 110.1 (C-6), 36.4 (CH_3_) ppm. MS-ESI(+): *m*/*z* 205.1 [M + H]^+^. HRMS-ESI(+): *m*/*z* calculated for C_9_H_9_N_4_S 205.0542 [M + H]^+^; found 205.0544.

##### 3.2.3.2 6-Ethyl-6*H*-thiazolo[5,4-*e*]indazol-2-amine, **5b**

Yield: 94%. M.p.: > 300 °C. ^1^H NMR (300 MHz, DMSO-*d*_6_): δ 8.02 (1H, d, *J* = 0.9 Hz, H-3), 7.52 (1H, dd, *J* = 8.9 and 0.9 Hz, H-7), 7.45 (1H, d, *J* = 8.9 Hz, H-6), 4.43 (2H, q, *J* = 7.3 Hz, *N*C*H*_2_CH_3_), 1.38 (2H, t, *J* = 7.3 Hz, *N*CH_2_C*H*_3_) ppm. ^13^C NMR (75 MHz, DMSO-*d*_6_): δ 165.4 (C-1′), 147.2 (C), 136.3 (C-3), 130.2 (C-7), 119.6 (C-7a), 118.6, 117.8 (C-3a), 107.8 (C-6), 43.9 (*N**C*H_2_CH_3_), 15.4 (*N*CH_2_*C*H_3_) ppm. MS-ESI(+): *m*/*z* 219.1 [M + H]^+^. HRMS-ESI(+): *m*/*z* calculated for C_10_H_11_N_4_S 219.0699 [M + H]^+^; found 219.0696.

##### 3.2.3.3 1-Methyl-1*H*-thiazolo[4,5-*g*]indazol-7-amine, **5c**

Yield: 86%. M.p.: > 300 °C. ^1^H NMR (300 MHz, DMSO-*d*_6_): δ 8.12 (1H, s, H-3), 7.76 (1H, d, *J* = 8.7 Hz, H-5), 7.31 (1H, d, *J* = 8.7 Hz, H-4), 4.15 (3H, s, *N*CH_3_) ppm. ^13^C NMR (75 MHz, DMSO-*d*_6_): δ 168.1 (C-1′), 134.8, 134.2 (C-3), 121.1, 120.5 (C-7a), 111.1 (C-5), 106.1 (C-4), 37.3 (*N*CH_3_) ppm. MS (MALDI): *m*/*z* 205.1 [M + H]^+^. HRMS-ESI(+): *m*/*z* calculated for C_9_H_9_N_4_S 205.0542 [M + H]^+^; found 205.0540.

##### 3.2.3.4 1-Ethyl-1*H*-thiazolo[4,5-*g*]indazol-7-amine, **5d**

Yield: 87%. M.p.: > 300 °C. ^1^H NMR (300 MHz, DMSO-*d*_6_): δ 8.15 (1H, s, H-3), 7.77 (1H, d, *J* = 8.7 Hz, H-5), 7.31 (1H, d, *J* = 8.7 Hz, H-4), 4.44 (2H, q, *J* = 7.2 Hz, *N*C*H*_2_CH_3_), 1.41 (3H, t, *J* = 7.2 Hz, *N*CH_2_C*H*_3_) ppm. ^13^C NMR (75 MHz, DMSO-*d*_6_): δ 168.0 (C-1′), 134.5, 133.9 (C-3), 121.2, 120.5 (C7a), 111.2 (C-5), 105.7 (C-4), 45.2 (*N**C*H_2_CH_3_), 16.3 (*N*CH_2_*C*H_3_) ppm. MS-ESI(+): *m*/*z* 219.1 [M + H]^+^. HRMS-ESI(+): *m*/*z* calculated for C_10_H_11_N_4_S 219.0699 [M + H]^+^; found 219.0702.

##### 3.2.3.5 8-Chloro-6-methyl-6*H*-thiazolo[5,4-*e*]indazol-2-amine, **5e**

Yield: 90%. M.p.: > 300 °C. ^1^H NMR (300 MHz, DMSO-*d*_6_): δ 7.69 (1H, d, *J* = 9.0 Hz, H-7), 7.59 (1H, d, *J* = 9.0 Hz, H-6), 4.04 (3H, s, *N*CH_3_). ^13^C NMR (75 MHz, DMSO-*d*_6_): δ 172.7 (C-1′), 167.5, 139.0 (C-3), 128.1 (C-7), 117.8 (C-7a), 115.0, 114.6 (C-3a), 110.2 (C-6), 21.5 (*N*CH_3_) ppm. MS-ESI(+): *m*/*z* 239.1 [M + H]^+^. HRMS-ESI(+): *m*/*z* calculated for C_9_H_8_N_4_ClS 239.0153 [M + H]^+^; found 239.0152.

#### 3.2.4 Synthesis of Thiazolo[5,4-*e*]indazol-2-yl)acetamides, **6a–c**

Each thiazolo[5,4-*e*]indazol-2-amine derivatives **5a**, **5b**, or **5e** (1 mmol) was dissolved in THF (5 mL), and the solution was cooled to 0 °C and added to trimethylamine (1 mmol). The mixture was kept at 0 °C and chloroacetyl chloride (2 mmol) was added dropwise. The ice bath was removed, and the reaction was stirred at room temperature for 1 h. Then, the mixture was poured into ice, and the solid obtained was filtered, washed with distilled water, and dried in the oven at 50 °C for 24 h affording the thiazolo[5,4-*e*]indazol-2-yl)acetamide derivatives **6a**–**c**.

##### 3.2.4.1 2-Chloro-*N*-(6-methyl-6*H*-thiazolo[5,4-*e*]indazol-2-yl)acetamide, **6a**

Yield: 87%. M.p.: > 300 °C. ^1^H NMR (300 MHz, DMSO-*d*_6_): δ 12.76 (1H, s, *N*H), 8.33 (2H, d, *J* = 0.9 Hz, H-3), 7.82 (1H, d, *J* = 9.0 Hz, H-6), 7.74 (1H, dd, *J* = 9.0 and 0.9 Hz, H-7), 4.48 (2H, s, CH_2_), 4.13 (3H, s, *N*CH_3_) ppm.^13^C NMR (75 MHz, DMSO-*d*_6_): δ 166.1 (C-1′), 155.3 (C=O), 143.7, 138.0 (C-3), 131.0 (C-7), 122.1 (C-7a), 120.5, 117.1 (C-3a), 109.6 (C-6), 42.8 (C-2′), 36.3 (*N*CH_3_) ppm. MS-ESI(+): *m*/*z* 281.0 [M + H]^+^. HRMS-ESI(+): *m*/*z* calculated for C_11_H_10_ClN_4_OS 281.0258 [M + H]^+^; found 281.0259.

##### 3.2.4.2 2-Chloro-*N*-(6-ethyl-6*H*-thiazolo[5,4-*e*]indazol-2-yl)acetamide, **6b**

Yield: 83%. M.p.: > 300 °C. ^1^H NMR (300 MHz, DMSO-*d*_6_): δ 12.75 (1H, s, *N*H), 8.34 (1H, s, H-3), 7.81 and 7.79 (2H, AB system, *J* = 9.1 Hz, H-6 and H-7), 4.56–4.48 (4H, m, CH_2_ and NC*H*_2_CH_3_), 1.43 (3H, t, *J* = 7.2 Hz, *N*CH_2_C*H*_3_) ppm. ^13^C NMR (75 MHz, DMSO-*d*_6_): δ 166.1 (C-1′), 155.3 (C=O), 143.7, 137.1 (C-3), 131.2 (C-7), 122.2 (C-7a), 120.4, 117.2 (C-3a), 109.5 (C-6), 44.0 (C-2′), 42.9 (*N*CH_2_CH_3_), 15.5 (*N*CH_2_*C*H_3_) ppm. MS-ESI(+): *m*/*z* 295.0 [M + H]^+^. HRMS-ESI(+): *m*/*z* calculated for C_12_H_12_ClN_4_OS 295.0415 [M + H]^+^; found 295.0415.

##### 3.2.4.3 2-Chloro-*N*-(8-chloro-6-methyl-6*H*-thiazolo[5,4-*e*]indazol-2-yl)acetamide, **6c**

Yield: 76%. M.p.: > 300 °C. ^1^H NMR (300 MHz, DMSO-*d*_6_): δ 12.88 (1H, s, *N*H), 7.91 (1H, d, *J* = 9.1 Hz, H-6), 7.80 (1H, d, *J* = 9.1 Hz, H-7), 4.49 (2H, s, CH_2_), 4.11 (3H, s, *N*CH_3_) ppm. ^13^C NMR (125 MHz, DMSO-*d*_6_): δ 166.4 (C-1′), 156.3 (C=O), 144.5, 139.4 (C-3), 129.0 (C-7), 122.1 (C-7a), 120.4, 114.5 (C-3a), 110.3 (C-6), 42.8 (C-2′), 36.7 (*N*CH_3_) ppm. MS-ESI(+): *m*/*z* 315.0 [M + H]^+^. HRMS-ESI(+): *m*/*z* calculated for C_11_H_9_Cl_2_N_4_OS 314.9869 [M + H]^+^; found 314.9867.

#### 3.2.5 Synthesis of Azido-thiazolo[5,4-*e*]indazole Derivatives **7a–c**

Sodium azide (10 equiv) was added to a solution of the adequate thiazolo[5,4-*e*]indazol-2-yl)acetamide derivatives **6a**–**c** (50 mg) in DMF (1 mL). Each resulting mixture was kept under stirring at room temperature until the TLC control confirmed the disappearance of the starting material and the formation of a single product (ca 24 h). After this period, the mixture was added to ice, and the precipitate obtained was filtered, washed with water, and dried in the oven at 50 °C for 24 h to afford in each case the expected azide as a light-yellow solid.

##### 3.2.5.1 2-Azido-*N*-(6-methyl-6*H*-thiazolo[5,4-*e*]indazol-2-yl)acetamide, **7a**

Yield: 69%. M.p.: 243.6–244.9 °C. ^1^H NMR (300 MHz, DMSO-*d*_6_): δ 8.04 (1H, s, H-3), 7.83 (1H, d, *J* = 8.9 Hz, H-6), 7.50 (1H, d, *J* = 8.9 Hz, H-7), 4.06 (2H, s, *N*CH_3_), 3.84 (3H, s, H-2′) ppm. MS-ESI(+): *m*/*z* 288.0 [M + H]^+^. HRMS-ESI(+): *m*/*z* calculated for C_11_H_10_N_7_OS 288.0662 [M + H]^+^; found 288.0662.

##### 3.2.5.2 2-Azido-*N*-(6-ethyl-6*H*-thiazolo[5,4-*e*]indazol-2-yl)acetamide, **7b**

Yield: 73%. M.p.: 220.8–221.5 °C. ^1^H NMR (300 MHz, DMSO-*d*_6_): 8.26 (1H, s, H-3), 7.76 and 7.70 (2H, AB system, *J* = 9.0 Hz, H-6 and H-7), 4.51 (2H, q, *J* = 7.3 Hz, *N*C*H*_2_CH_3_), 4.18 (2H, s, H-2′), 1.42 (3H, t, *J* = 7.3 Hz, *N*CH_2_C*H*_3_) ppm. ^13^C NMR (75 MHz, DMSO-*d*_6_): δ 168.7 (C-1′), 157.5 (C=O), 144.0, 136.9 (C-3), 131.0 (C-7), 122.2 (C-7a), 120.2, 117.4(C-3a), 108.8 (C-6), 51.8 (C-2′), 44.0 (*N*CH_2_CH_3_), 15.5 (*N*CH_2_*C*H_3_) ppm. MS-ESI(+): *m*/*z* 302.0 [M + H]^+^. HRMS-ESI(+): *m*/*z* calculated for C_12_H_12_N_7_OS 302.0819 [M + H]^+^; found 302.0819.

##### 3.2.5.3 2-Azido-*N*-(8-chloro-6-methyl-6*H*-thiazolo[5,4-*e*]indazol-2-yl)acetamide, **7c**

Yield: 72%. M.p.: 194.0–195.0 °C. ^1^H NMR (300 MHz, DMSO-*d*_6_): δ 7.65 (1H, d, *J* = 9.0 Hz, H-6), 7.79 (1H, d, *J* = 9.0 Hz, H-7), 4.04 (2H, s, H-2′), 3.85 (3H, s, *N*CH_3_) ppm. MS-ESI(+): *m*/*z* 322.0 [M + H]^+^. HRMS-ESI(+): *m*/*z* calculated for C_11_H_9_ClN_7_OS 322.0272 [M + H]^+^; found 322.0269.

#### 3.2.6 1,3-Dippolar Cycloaddition of Azido-thiazolo[5,4-*e*]indazole Derivatives **7a–c** with 1-ethynyl-3,5-*bis*(trifluoromethyl)-benzene

Copper sulfate hydrated (0.005 mmol) and sodium ascorbate (0.01 mmol) was added to a solution of the appropriate azido-thiazolo[5,4-*e*]indazole derivatives **7a**–**c** (0.024 mmol) and 1-ethynyl-3,5-bis(trifluoromethyl)-benzene (0.024 mmol) in DMF (1.5 mL). The reaction in each case was stirred at room temperature and the TLC control showed the total consumption of the starting material after 1 h. After this period, the product was precipitated with water, the solid was filtered, sequentially washed with water and ethyl ether, and dried in the oven for 24 h to give the triazolo-thiazolo[5,4-*e*]indazole derivatives **TI45a–c** as white solids.

##### 3.2.6.1 2-(4-(3,5-*bis*(Trifluoromethyl)phenyl)-1*H*-1,2,3-triazol-1-yl)-*N*-(6-methyl-6*H*-thiazolo[5,4-*e*]indazol-2-yl)acetamide, **TI45a**

Yield: 87%. M.p.: 270.4–271.9 °C. ^1^H NMR (300 MHz, DMSO-*d*_6_): δ 13.02 (1H, s, *N*H), 9.09 (1H, s, H-4″), 8.59 (2H, s, H-2″,6″), 8.30 (1H, s, H-3), 8.11 (1H, s, H-3′), 7.85 (1H, d, *J* = 9.0 Hz, H-6), 7.75 (1H, d, *J* = 9.0 Hz, H-7), 5.69 (2H, s, H-2′), 4.13 (3H, s, *N*CH_3_) ppm. MS-ESI(+): *m*/*z* 526.0 [M + H]^+^. HRMS-ESI(+): *m*/*z* calculated for C_21_H_14_F_6_N_7_OS 526.0879 [M + H]^+^; found 526.0882.

##### 3.2.6.2 2-(4-(3,5-*bis*(Trifluoromethyl)phenyl)-1*H*-1,2,3-triazol-1-yl)-*N*-(6-ethyl-6*H*-thiazolo[5,4-*e*]indazol-2-yl)acetamide, **TI45b**

Yield: 80%. M.p.: 275.0–276.9 °C. ^1^H NMR (300 MHz, DMSO-*d*_6_): δ 9.05 (1H, s, H-4″), 8.59 (2H, s, H-2″,6″), 8.23–8.03 (2H, m, H-3 and H-3′), 7.80–7.53 (2H, m, H-6 and H-7), 5.45 (2H, s, H-2′), 4.48 (3H, q, *J* = 7.2 Hz, *N*C*H*_2_CH_3_), 1.41 (3H, t, *J* = 7.2 Hz, *N*CH_2_C*H*_3_)H) ppm. ^13^C NMR (75 MHz, DMSO-*d*_6_): δ 165.4, 144.2, 133.6, 131.6, 131.4, 131.1, 126.0, 125.9, 125.8, 125.7, 125.5, 122.2, 122.1, 120.8, 109.7, 36.3, 29.4, 10.4 ppm. MS-ESI(+): *m*/*z* 540.0 [M + H]^+^. HRMS-ESI(+): *m*/*z* calculated for C_22_H_16_F_6_N_7_OS 540.1036 [M + H]^+^; found 540.1038.

##### 3.2.6.3 2-(4-(3,5-*bis*(Trifluoromethyl)phenyl)-1*H*-1,2,3-triazol-1-yl)-*N*-(8-chloro-6-methyl-6*H*-thiazolo[5,4-*e*]indazol-2-yl)acetamide, **TI45c**

Yield: 81%. M.p.: 257.8–259.0 °C. ^1^H NMR (300 MHz, DMSO-*d*_6_): δ 9.09 (1H, s, H-4″), 8.59 (2H, s, H-2″,6″), 8.11 (1H, s, H-3′), 7.85 (1H, d, *J* = 9.0 Hz, H-6), 7.74 (1H, d, *J* = 9.0 Hz, H-7), 5.69 (2H, s, H-2′), 4.13 (3H, s, *N*CH_3_) ppm. ^13^C NMR (75 MHz, DMSO-*d*_6_): δ 158.7, 144.2, 133.8, 131.8, 131.3, 130.9, 125.9, 125.8, 126.6, 125.5, 109.0, 121.9, 121.3, 119.0, 43.4, 15.5 ppm. MS-ESI(+): *m*/*z* 559.9 [M + H]^+^. HRMS-ESI(+): *m*/*z* calculated for C_21_H_13_ClF_6_N_7_OS 560.0490 [M + H]^+^; found 560.0489.

#### 3.2.7 Synthesis of Carbazole-Thiazolo[5,4-*e*]indazole Derivatives **TI58a–c**

Thiazolo[5,4-*e*]indazol-2-yl)acetamides derivatives **6a**–**c** (0.035 mmol) were added in each case to a stirred solution of carbazole (0.071 mmol, 2 equiv) and of sodium hydride (0.35 mmol, 10 equiv) in a DMF/THF (1:2) mixture (3 mL). Each reaction mixture was heated at 65 °C for 24 h and after this period the solvent was removed under reduced pressure. The resulting solid was dissolved in ethyl acetate, washed with water and the aqueous layer was back-extracted with ethyl acetate. The combined organic extracts were dried with anhydrous Na_2_SO_4_, filtered and after the partial removal of the solvent under reduced pressure afforded a yellow oil. The crude mixture was purified by preparative thin-layer chromatography using a hexane-ethyl/acetate (3:2) mixture as the eluent. The main fraction isolated afforded the carbazole-thiazoloindazole derivatives **TI58a–c** as white solids.

##### 3.2.7.1 2-(9*H*-carbazol-9-yl)-*N*-(6-methyl-6*H*-thiazolo[5,4-*e*]indazol-2-yl)acetamide, **TI58a**

Yield: 77%. M.p.: 257.2–258.4 °C. ^1^H NMR (300 MHz, CDCl_3_): δ 8.18 (2H, d, *J* = 9.0 Hz, H-4″), 8.10 (1H, s, H-3), 7.60 (1H, d, *J* = 8.9 Hz, H-6), 7.56–7.48 (2H, m, H-1″), 7.43–7.32 (5H, m, H-7, H-2″ and H-3″), 5.22 (2H, s, H-2′), 4.12 (s, *N*CH_3_) ppm. ^13^C NMR (125 MHz, CDCl_3_): δ 169.5 (C=O), 161.7 (C-1′), 154.7, 140.2 (C-3), 138.0, (C-1″a), 131.3 (C-4″), 130.6 (C-6), 126.8 (C-7a), 123.9, 121.0 (C-3a), 119.8 (C-7), 117.3 (C-1″), 114.9 (C-3″), 108.8 (C-4″a), 108.4 (C-2″), 47.1 (C-2′), 36.1 (*N*CH_3_) ppm. MS-ESI(+): *m*/*z* 412.1 [M + H]^+^. HRMS-ESI(+): *m*/*z* calculated for C_23_H_18_N_5_OS 412.1227 [M + H]^+^; found 412.1230.

##### 3.2.7.2 2-(9*H*-carbazol-9-yl)-*N*-(6-ethyl-6*H*-thiazolo[5,4-*e*]indazol-2-yl)acetamide, **TI58b**

Yield: 71%. M.p.: 279.8–281.0 °C. ^1^H NMR (300 MHz, CDCl_3_): δ 8.17 (2H, d, *J* = 9.0 Hz, H-4″), 8.11 (1H, s, H-3), 7.61 (1H, bs, *N*H), 7.59 (1H, d, *J* = 9.0 Hz, H-6), 7.55–7.49 (2H, m, H-1″), 7.43–7.32 (5H, m, H-7, H-2″ and H-3″), 5.22 (2H, s, H-2′), 4.48 (2H, q, *J* = 7.3 Hz, *N*CH_2_CH_3_), 1.54 (2H, t, *J* = 7.3 Hz, *N*CH_2_CH_3_) ppm. ^13^C NMR (125 MHz, CDCl_3_): δ 166.8 (C=O), 157.06 (C-1′), 140.2, 136.8, 130.7 (C-3), 126.8 (C-1″), 123.9, 121.0 (C-4″), 119.9 (H-6), 117.94 (C-3″), 108.5 (C-4″a), 108.4 (C-2″), 47.1 (C-2′), 44.3 (*N*CH_2_CH_3_), 15.04 (*N*CH_2_CH_3_) ppm. MS-ESI(+): *m*/*z* 426.2 [M + H]^+^. HRMS-ESI(+): *m*/*z* calculated for C_24_H_20_N_5_OS 426.1383 [M + H]^+^; found 426.1385.

##### 3.2.7.3 2-(9*H*-carbazol-9-yl)-*N*-(8-chloro-6-methyl-6*H*-thiazolo[5,4-*e*]indazol-2-yl)acetamide, **TI58c**

Yield: 66%. M.p.: 274.3–276.0 °C. ^1^H NMR (500 MHz, CDCl_3_): δ 8.18 (2H, d, *J* = 5.8 Hz, H-4″), 7.64 (1H, d, *J* = 9.1 Hz, H-6), 7.55–7.50 (2H, m, H-1″), 7.43–7.32 (5H, m, H-7, H-2″ and H-3″), 5.24 (2H, s, H-2′), 4.07 (3H, s, *N*CH_3_) ppm.^13^C NMR (125 MHz, CDCl_3_): δ 166.9 (C=O), 160.2 (C-1′), 155.6, 142.8 (C-3), 140.2 (C-1″a), 139.3 (C-4″), 130.8 (C-6), 126.9 (C-7a), 123.9 (C-3a), 121.3 (C-7), 121.0 (C-1″), 115.2 (C-3″), 108.8 (C-4″a), 108.4 (C-2″), 47.1 (C-2′), 36.4 (*N*CH_3_) ppm. MS-ESI(+): *m*/*z* 446.1 [M + H]^+^. HRMS-ESI(+): *m*/*z* calculated for C_23_H_17_ClN_5_OS 446.0837 [M + H]^+^; found 446.0836.

### 3.3 Single-Crystal X-Ray Diffraction Studies

Single crystals of compounds **5d**, **6b**, **TI45c**, **TI58a,** and **TI58c** were manually harvested from the crystallization vial and immersed in highly viscous FOMBLIN Y perfluoropolyether vacuum oil (LVAC 140/13, Sigma-Aldrich) to avoid degradation caused by the evaporation of the solvents.^57^ Crystals were mounted on either Hampton Research CryoLoops or MiTeGen MicroLoops, typically with the help of a Stemi 2000 stereomicroscope equipped with Carl Zeiss lenses.

Crystal data for compounds **5d** and **TI45c** were collected at 150(2) K on a Bruker Bruker D8 QUEST equipped with a Mo Kα sealed tube (λ = 0.71073 Å), a multilayer TRIUMPH X-ray mirror, and a PHOTON III detector, controlled by the APEX4 software package,^58^ and equipped with an Oxford Cryosystems Series 700 cryostream monitored remotely using the software interface Cryopad.^59^ Diffraction images were processed using the software package SAINT+,^60^ and data were corrected for absorption by the multiscan semiempirical method implemented in SADABS 2016/2.^61^

Crystal data for compounds **6b**, **TI58a**, and **TI58c**, on the other hand, were collected at 150(2) K on a RIGAKU XtaLAB Synergy-*i* instrument with a Mo Kα (λ = 0.71073 Å) PhotonJet-i microsource, a HyPix3000 detector controlled by the CrysAlisPro^62^ software and equipped with an Oxford Cryosystems Series 800 cryostream. Diffraction images were processed using the CrysAlisPro software and the data were corrected for absorption by the multiscan absorption correction using spherical harmonics implemented in SCALE3 ABSPACK scaling algorithm.

The structures were solved using the algorithm implemented in SHELXT-2014/5,^63^ which allowed the immediate location of almost all of the heaviest atoms composing the molecular units. The remaining missing and misplaced non-hydrogen atoms were located from difference Fourier maps calculated from successive full-matrix least-squares refinement cycles on *F*^2^ using the latest SHELXL from the 2019/2 release.^64^ All structural refinements were performed using the graphical interface ShelXle^65^ and Olex2.^66^

Hydrogen atoms bound to carbon were placed at their idealized positions using *HFIX* instructions in SHELXL: 43 (for aromatic carbon atoms), 23 (for the – CH_2_– groups), and 127 (for the disordered methyl groups). These hydrogen atoms were included in subsequent refinement cycles with isotropic thermal displacement parameters (*U*_iso_) fixed at 1.2 (for the aromatic and – CH_2_– hydrogen atoms) or 1.5 × *U*_eq_ (solely for those associated with the methyl group) of the parent carbon atoms.

The last difference Fourier map synthesis showed: for **5d**, the highest peak (0.332 eÅ^–3^) and the deepest hole (−0.358 eÅ^–3^) located at 0.59 and 0.36 Å from H1B, respectively; for **6b**, the highest peak (0.777 eÅ^–3^) and the deepest hole (−0.625 eÅ^–3^) located at 0.98 and 0.62 Å from H11B and Cl4, respectively; for **TI45c**, the highest peak (0.645 eÅ^–3^) and the deepest hole (−0.383 eÅ^–3^) located at 1.27 and 0.60 Å from S1 and S4, respectively; for **TI58a**, the highest peak (0.332 eÅ^–3^) and the deepest hole (−0.308 eÅ^–3^) located at 0.26 and 0.64 Å from H22 and C53, respectively; for **TI58c**, the highest peak (0.291 eÅ^–3^) and the deepest hole (−0.376 eÅ^–3^) located at 1.48 and 0.63 Å from S2 and Cl2, respectively. All structural drawings have been created using the software package Crystal Impact Diamond.^67^

Crystallographic data (including structure factors) for the crystal structure of compounds **5d**, **6b**, **TI45c**, **TI58a**, and **TI58c** have been deposited with the Cambridge Crystallographic Data Centre. Copies of the data can be obtained free of charge by application to CCDC, 12 Union Road, Cambridge CB2 2EZ, U.K. FAX: (+44) 1223 336033. E-mail: deposit@ccdc.cam.ac.uk.

#### 3.3.1 Crystal Data for **5d**

C_11_H_11_N_5_S_2_, *M* = 277.37, Triclinic, space group *P*-1, *Z* = 2, *a* = 7.7047(12) Å, *b* = 9.1888(14) Å, *c* = 10.3881(17) Å, α = 68.081(5)°, β = 71.004(5)°, γ = 71.347(5)°, *V* = 628.42(17) Å^3^, μ(Mo–Kα) = 0.412 mm^–1^, *D*_c_ = 1.466 g cm^–3^, colorless plate with crystal size of 0.16 × 0.09 × 0.02 mm^3^. Of a total of 11206 reflections collected, 2274 were independent (*R*_int_ = 0.0193). Final *R*1 = 0.0261 [*I* > 2σ(*I*)] and *wR*2 = 0.0681 (all data). Data completeness to theta = 25.24°, 99.0%. CCDC 2297650.

#### 3.3.2 Crystal Data for **6b**

C_33_H_27_Cl_3_N_12_O_3_S_3_, *M* = 842.19, Orthorhombic, space group *Aba*2, *Z* = 8, *a* = 19.5761(9) Å, *b* = 49.083(2) Å, *c* = 7.6762(5) Å, *V* = 7375.7(7) Å^3^, μ(Mo–Kα) = 0.473 mm^–1^, *D*_c_ = 1.517 g cm^–3^, colorless plate with crystal size of 0.13 × 0.12 × 0.03 mm^3^. Of a total of 44823 reflections collected, 6718 were independent (*R*_int_ = 0.1202). Final *R*1 = 0.0801 [*I* > 2σ(*I*)] and *wR*2 = 0.2017 (all data). Data completeness to theta = 25.24°, 99.6%. CCDC 2297654.

#### 3.3.3 Crystal Data for **TI45c**

C_25_H_24_ClF_6_N_7_O_3_S_3_, *M* = 716.14, Orthorhombic, space group *P*2_1_2_1_2_1_, *Z* = 4, *a* = 4.7624(5)Å, *b* = 24.487(3) Å, *c* = 26.504(3) Å, *V* = 3090.8(6) Å^3^, μ(Mo–Kα) = 0.404 mm^–1^, *D*_c_ = 1.539 g cm^–3^, colorless needle with crystal size of 0.23 × 0.09 × 0.08 mm^3^. Of a total of 38389 reflections collected, 5635 were independent (*R*_int_ = 0.0697). Final *R*1 = 0.0693 [*I* > 2σ(*I*)] and *wR*2 = 0.1877 (all data). Data completeness to theta = 25.24°, 99.5%. CCDC 2297653.

#### 3.3.4 Crystal Data for **TI58a**

C_50_H_46_N_10_O_4_S_2_, *M* = 915.09, Orthorhombic, space group *Pbca*, *Z* = 8, *a* = 12.5901(7) Å, *b* = 19.9359(13) Å, *c* = 35.2244(18) Å, *V* = 8841.1(9) Å^3^, μ(Mo–Kα) = 0.180 mm^–1^, *D*_c_ = 1.375 g cm^–3^, colorless plate with crystal size of 0.32 × 0.18 × 0.06 mm^3^. Of a total of 64,200 reflections collected, 8049 were independent (*R*_int_ = 0.1727). Final *R*1 = 0.0727 [*I* > 2σ(*I*)] and *wR*2 = 0.1641 (all data). Data completeness to theta = 25.24°, 99.6%. CCDC 2297652.

#### 3.3.5 Crystal Data for **TI58c**

C_23_H_16_ClN_5_OS, *M* = 445.92, Triclinic, space group *P*-1, *Z* = 4, *a* = 9.3284(4) Å, *b* = 14.4219(7) Å, *c* = 15.0593(7) Å, α = 86.687(4)°, β = 83.820(4)°, γ = 87.883(4) °, *V* = 2009.87(16) Å^3^, μ(Mo–Kα) = 0.321 mm^–1^, *D*_c_ = 1.474 g cm^–3^, colorless needle with crystal size of 0.20 × 0.04 × 0.02 mm^3^. Of a total of 25459 reflections collected, 7031 were independent (*R*_int_ = 0.0777). Final *R*1 = 0.0641 [*I* > 2σ(*I*)] and *wR*2 = 0.1317(all data). Data completeness to theta = 25.24°, 95.7%. CCDC 2297651.

### 3.4 Target Prediction

The ready-to-use Mondrian conformal prediction (MCP) models for target prediction that are provided by the ChEMBL team and are got as docker image through the following link: “*docker run -p 8080:8080 chembl/mcp*”, were used to predict targets for the thiazoloindazole template (Figure 2). The docker image was accessed through the Docker v20.10.14.^68^ The MCP models developed by Bosc et al.^47^ have been reimplemented with LightGBM, which is a gradient boosting framework that uses tree-based learning algorithms, in Python by Scikit-learn version 0.19,^69^ and the conformal prediction framework was developed using the nonconformist package version 2.1.0.^70^

### 3.5 Virtual Library of Thiazoloindazole-Based Derivatives

In order to synthesize the most promising thiazoloindazole-based derivatives, which will be subjected to virtual screening against AChE activity by molecular docking, the following virtual libraries were built, comprising 177 molecules (Figure S73). The virtual derivatives were assembled using the Chemaxon Reactor program version 22.11.0 (Chemaxon Ltd., Budapest, Hungary). The strategy used to build the virtual library started from the following reaction scheme (eq 1), through a combinatorial approach of two blocks, namely, reactants library 1 and reactants library 2, and visualizing the reaction products. The libraries of reactants 1 and 2 comprise 3 cores (TlXa, TlXb, and TlXc) and 59 substituent groups (R), respectively, in accordance with Figure S73.
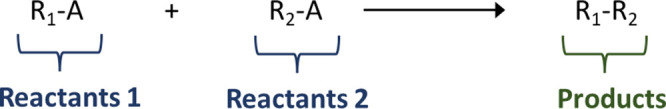
1

### 3.6 Molecular Docking Studies

The optimization of the 3D structure of the synthesized precursors, fused-amino-thiazoloindazole **5a**–**e**, thiazolo[5,4-*e*]indazol-2-acetamides **6a**–**c** and the azido-thiazolo[5,4-*e*]indazole derivatives **7a**–**c**, the 177 compounds of the virtual library (according to Figure S73), the thiazoloindazole-template (Figure 2), and the positive control (donepezil, AChE inhibitor used for Alzheimer disease therapy) was carried out with JChem CXCALC (JChem 22.11.0, 2022, Chemaxon Ltd., Budapest, Hungary). The software program OpenBabel (version 2.3.1)^71^ was used to convert the mol2 files to PDBQT files. PDBQT files were used for docking to AChE enzyme (PDB ID 4EY4) with AutoDock Vina (version 1.1).^72^ Water molecules, ions, and ligands were removed from 4EY4 prior to docking using the AutoDockTools (http://mgltools.scripps.edu/, accessed on 19 May 2022). The search space coordinates were center *X*: −12.903 *Y*: −39.296 *Z*: 26.578; dimensions *X*: 40.000 *Y*: 40.000 *Z*: 40.000. Ligand tethering of the AChE enzyme was performed by regulating the genetic algorithm (GA) parameters, using 10 runs of the GA criteria. DockThor, a web service for molecular docking simulation (https://dockthor.lncc.br/v2/, accessed on 12 June 2024), was used to perform molecular docking of the three most promising thiazoloindazole-based derivatives (**TI45c**, **Tl45a** and **TI45b**, Table 1) and the positive control (donepezil) against AChE enzyme (PDB ID 4EY4).^50^ The search space coordinates were center *X*: −12.903 *Y*: −39.296 *Z*: 26.578; dimensions *X*: 20,000 *Y*: 20,000 *Z*: 20,000. AChE enzyme ligand tethering was performed by regulating the parameters of the GA, using 12,750 and 500,000 runs, population size, and number of evaluations of the GA criteria, respectively. The docking binding poses were visualized with PyMOL Molecular Graphics System, Version 2.0 Schrödinger, LLC, UCSF Chimera,^73^ and Protein–Ligand Interaction Profiler (PLIP) web tool.^74^

### 3.7 AChE/BuChE Inhibitory Activity Assays

The inhibitory activity of the selected compounds toward AChE and BuChE was evaluated spectrophotometrically through a modified 96-well microplate Ellman’s method.^54^ The enzyme solutions of both *Electrophorus electricus* AChE (eeAChE) and equine serum BuChE were prepared as 0.025 U/mL in phosphate buffer, at pH 8, from stock solutions of 5.05 and 7.50 U/mL, respectively. In the selection of eeAChE, we took into account that this enzyme displays a considerable sequence identity (>85%) with the human enzyme hAChE used for the theoretical studies. Furthermore, it has also been demonstrated that hAChE and eeAChE present fully overlapping binding pockets, with crystallography of huprine presenting similar binding modes when docked to both enzymes.^55^ Additional advantages of eeAChE in this primary screening were its prompt availability and low cost. The stock solutions of the selected compounds were prepared in DMSO at concentrations that would allow the assay performance with a percentage of DMSO below 1% in the wells. The remaining assay solutions were all prepared in 0.1 M phosphate buffer, at pH 8, and consisted of 0.5 mM 5,5′-dithiobis(2-nitrobenzoic acid) (DTNB), 2.5 mM acetylthiocholine iodide (ATChI), for the inhibition of AChE, and 2.5 mM butyrylthiocholine iodide (BuTChI), for the inhibition of BuChE. The assay began with 5 min incubation at 37 °C of 50 μL of the test compound (or phosphate buffer at pH 8, i.e., blank samples) and 100 μL of the enzyme (eeAChE or BuChE). After the incubation period, 50 μL of ATChI and 50 μL of DTNB were added to each well, thus initiating the enzymatic reaction. The absorbance values were measured at 415 nm, at every 2.5 min, for 15 min, using a microplate reader (Synergy multimode reader; BioTek). The absorbance was measured at different concentrations, with a dilution factor of 2 between each concentration, converted into % of inhibition, and plotted against the used inhibitor concentration. The half-maximal inhibitory concentrations (IC_50_) were derived from nonlinear regression analysis, using the “log (inhibitor) vs response (three parameters)” function in GraphPad Prism 8.0.2 software (GraphPad Software, Inc., La Jolla, CA). All the reactions were performed in triplicate, including donepezil used as the standard inhibitor.

## 4 Conclusions

In this work, we unveil a remarkable advancement in developing novel thiazoloindazole-based derivatives that show great potential as efficient AChE inhibitors for treating neurodegenerative disorders. We identified potential promising inhibitors through computational studies using molecular docking by predicting their binding interactions with the AChE enzyme. Many of the thiazoloindazole-based derivatives exhibited enhanced Δ*G*_B_ (less than or equal to −12.0 kcal/mol) compared to donepezil (Δ*G*_B_ = −8.8 kcal/mol), a well-known AChE inhibitor used for Alzheimer’s disease treatment.

Guided by these promising results, two different series of compounds were synthesized, one bearing a triazole moiety (**Tl45a–c**) and the other a carbazole moiety (**Tl58a–c**). The synthesis involved nucleophilic substitution and Cu(I)-catalyzed azide–alkyne cycloaddition (CuAAC) reactions, providing efficient and practical pathways to obtain structurally diverse thiazoloindazole-based derivatives in very good to excellent yields.

To validate our predictions, we conducted comprehensive experimental evaluations of the synthesized compounds **Tl45a–c** and **Tl58a–c**, along with their precursors, to assess their AChE and BuChE inhibitory activities.

This evaluation showed that thiazoloindazole-based **6b** core derivatives as selective AChE inhibitors, with IC_50_ values below 1.0 μM and superior to 50 μM for BuChE, supporting theoretical studies. The structure–activity relationship (SAR) analysis revealed that derivatives featuring the bis(trifluoromethyl)phenyl-triazolyl substituent demonstrated the most promising activity against AChE. On the other hand, the presence of a carbazolyl moiety seemed to reduce AChE activity when compared to more flexible substituents. Notably, derivative **Tl45b** emerged as a standout AChE inhibitor, boating the best IC_50_ value (0.071 μM), likely attributed to its preferential interaction with two PAS residues of the AChE enzyme, namely with the Trp286 and Tyr72 residues when compared to the other evaluated derivatives. Moreover, derivatives **6a**–**c** with moderately activating electron-donating substituents, such as the amide group, exhibited superior AChE activity over those featuring strongly activating electron-donating substituents like the amine group present in derivatives **5a**–**c**.

This study demonstrates an exemplary combination of computational predictions and experimental evaluations, paving the way to developing and fine-tuning thiazoloindazole core as a potent and selective AChE inhibitor. This combination of molecular docking and experimental synthesis has allowed us to strategically modify the thiazoloindazole core with the appropriate units, thereby enhancing their AChE inhibitor properties and significantly expediting the drug discovery process while minimizing costs. These findings provide valuable insights into the potential of thiazoloindazole-based derivatives for the treatment of neurodegenerative disorders such as Alzheimer’s disease, opening new avenues for drug development in the persistent fight against these debilitating conditions.

## Supporting Information Available

Virtual library, NMR and mass [MS-ESI(+) and HRMS-ESI(+)] spectra, single-crystal X-ray diffraction complementary information, and molecular docking data against AChE enzyme data (PDF)
