# Supplementary material for: Design of Promising Thiazoloindazole-Based Acetylcholinesterase Inhibitors Guided by Molecular Docking and Experimental Insights
Source: ACS Chem Neurosci. 2024 Jul 22;15(15):2853–69. doi: 10.1021/acschemneuro.4c00241 (PMC11311138; doi:10.1021/acschemneuro.4c00241)
Supplement: Supplementary file 1 — cn4c00241_si_001.pdf [file cn4c00241_si_001.pdf]

Supporting Information

Design of Promising Thiazoloindazole-based Acetylcholinesterase Inhibitors Guided by Molecular Docking and Experimental Insights

Fatima Ezzahra Laghchioua,[1] Carlos F. M. Silva,[2] Diana C. G. A. Pinto,[2] José A. S. Cavaleiro,[2] Ricardo F. Mendes,[3] Filipe A. Almeida Paz,[3] Maria A. F. Faustino,[2] El Mostapha Rakib,[1,4,*] Maria da Graça P. M. S. Neves,[2] Florbela Pereira,[5,*] Nuno M. M. Moura[2,*]

[1] *Laboratory of Molecular Chemistry, Materials and Catalysis, Faculty of Sciences and Technics, Sultan Moulay Slimane University, Beni-Mellal, BP 523, Morocco*

[2] *LAQV-REQUIMTE, Department of Chemistry, University of Aveiro, 3810-193 Aveiro, Portugal*

[3] *CICECO – Aveiro Institute of Materials, Department of Chemistry, University of Aveiro, 3810-193 Aveiro, Portugal*

[4] *Higher School of Technology, Sultan Moulay Slimane University, BP 336, Fkih Ben Salah, Morocco*

[5] *LAQV-REQUIMTE, NOVA School of Science and Technology, Universidade Nova de Lisboa, 2829-516 Caparica, Portugal*

Corresponding authors: E.RAKIB@usms.ma (El Mostapha Rakib); florbela.pereira@fct.unl.pt (Florbela Pereira); nmoura@ua.pt (Nuno M. M. Moura)

Table of contents

1. NMR spectra **S4**
2. MS-ESI(+) spectra **S22**
3. HRMS-ESI(+) spectra **S31**
4.Single-crystal X-ray diffraction **S48**
5. Virtual library **S49**
6. Molecular docking data against AChE enzyme **S51**

1. NMR spectra

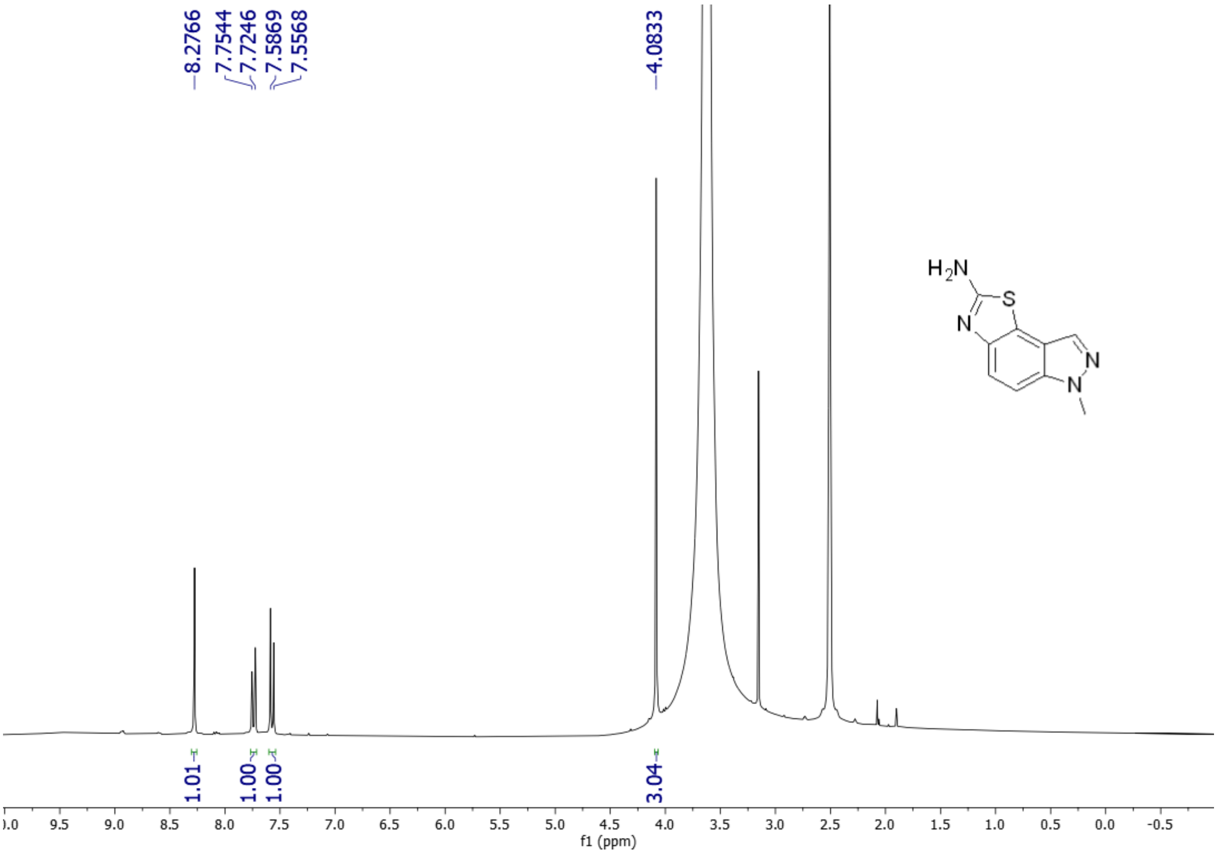

Figure S1. ^{1}H NMR spectrum of compound **5a** in DMSO-d_6.

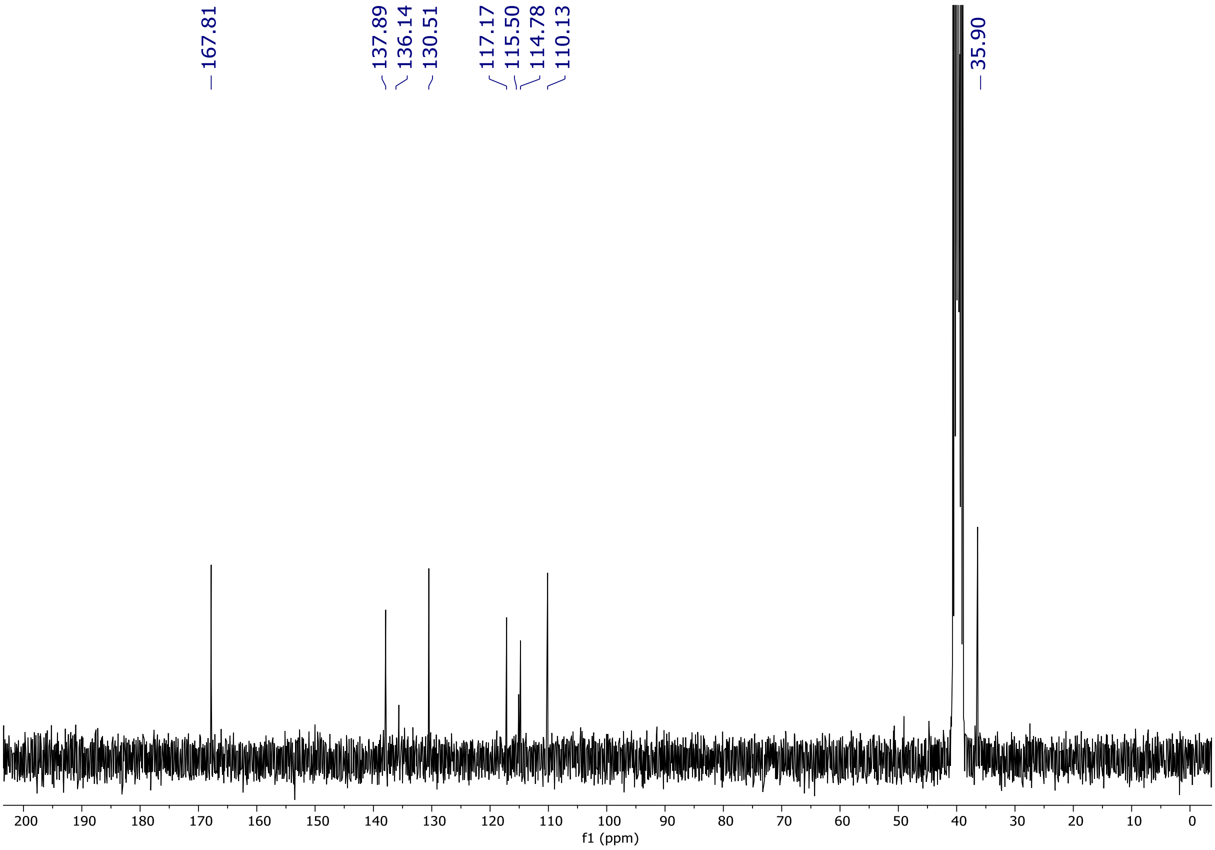

Figure S2. ^{13}C NMR spectrum of compound **5a** in DMSO-d_6.

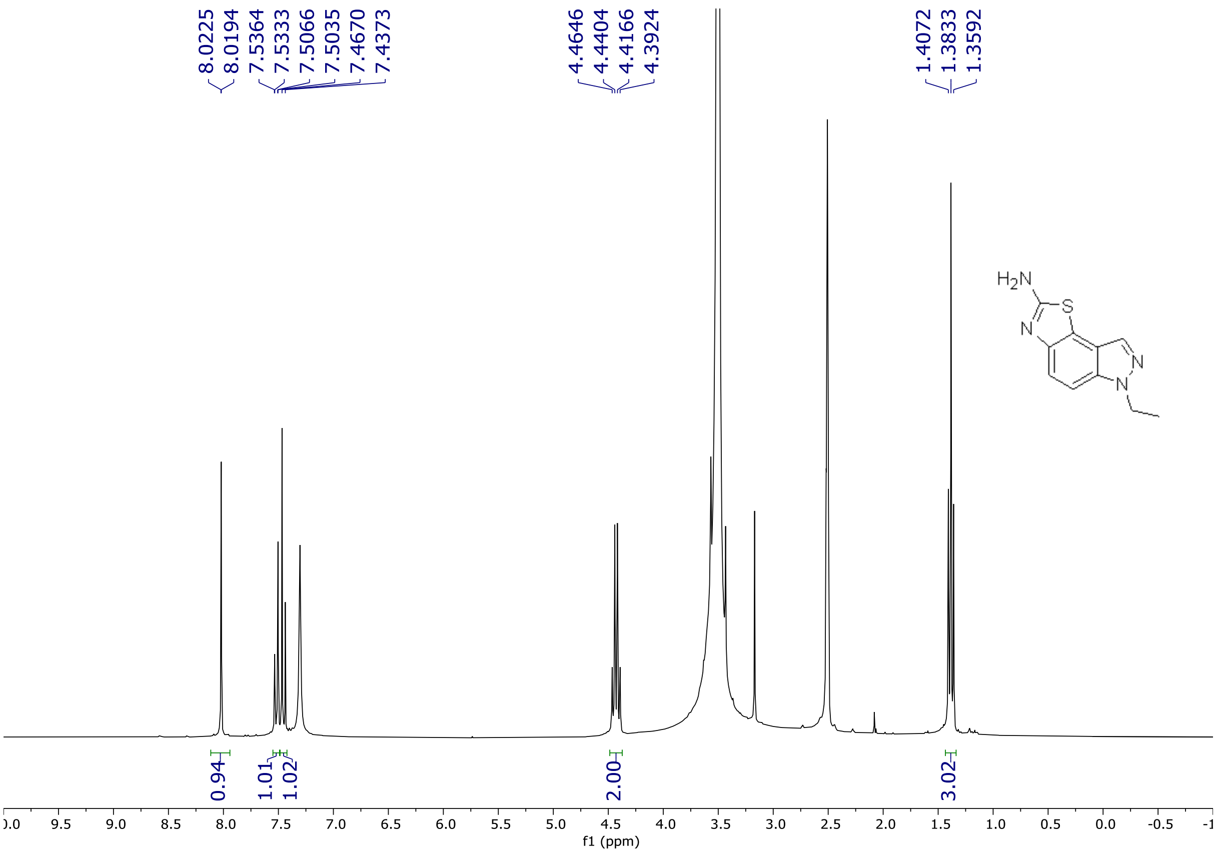

Figure S3. ^{1}H NMR spectrum of compound **5b** in DMSO-d6.

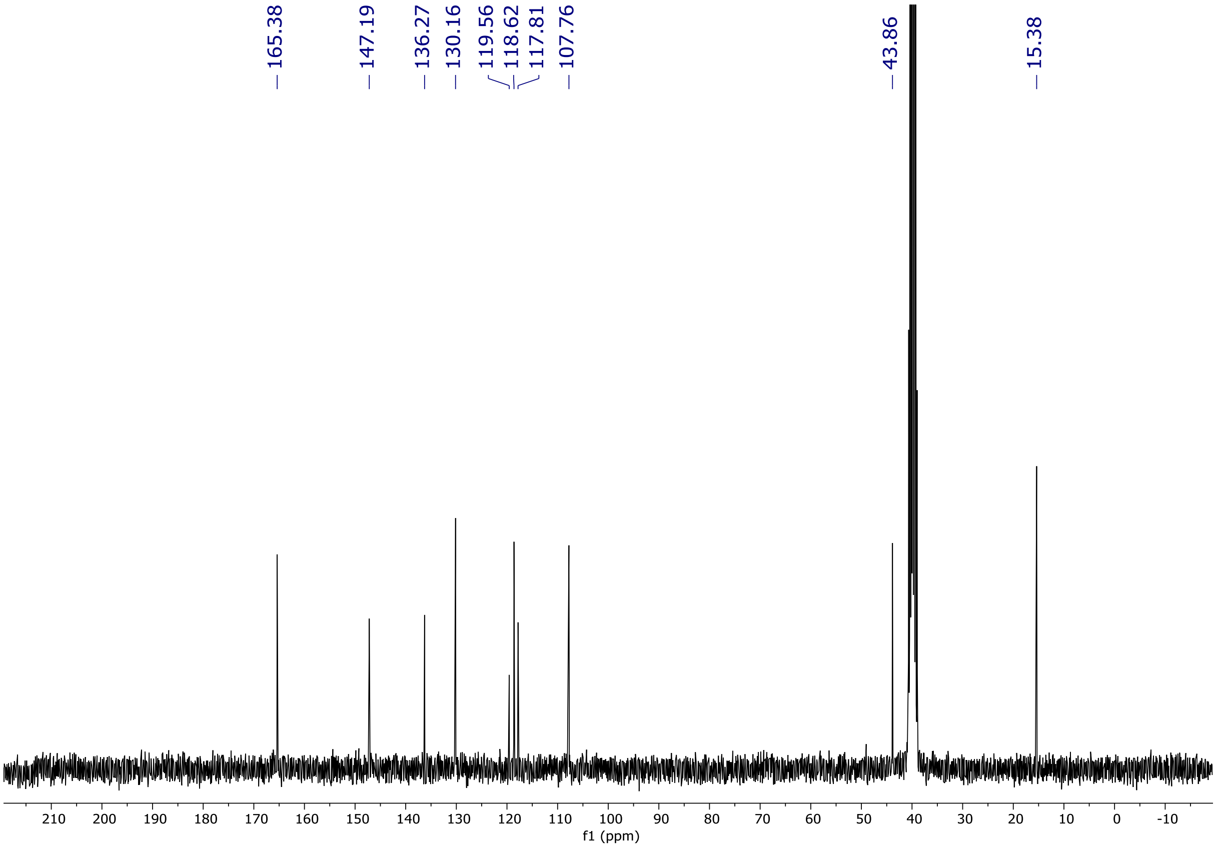

Figure S4. ^{13}C NMR spectrum of compound **5b** in DMSO-d6.

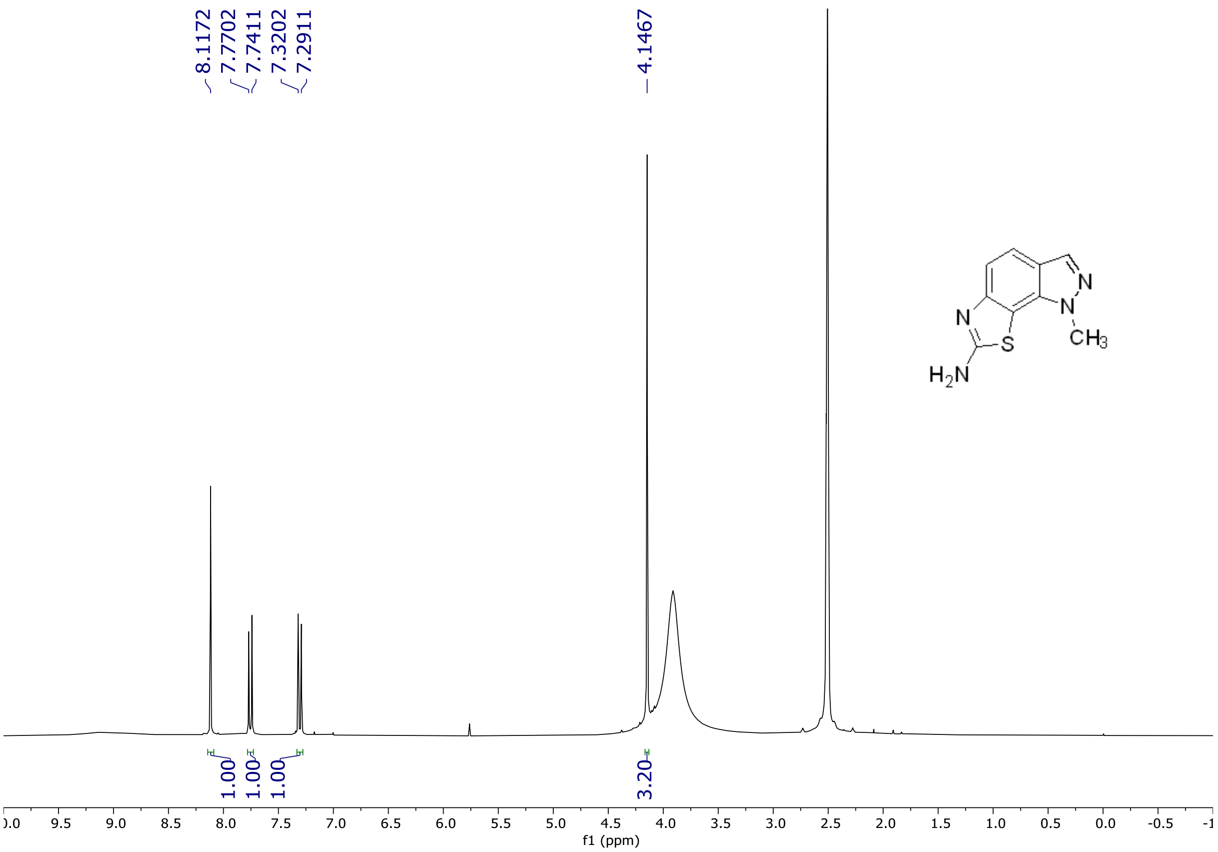

Figure S5. ^{1}H NMR spectrum of compound **5c** in DMSO-d$_6$.

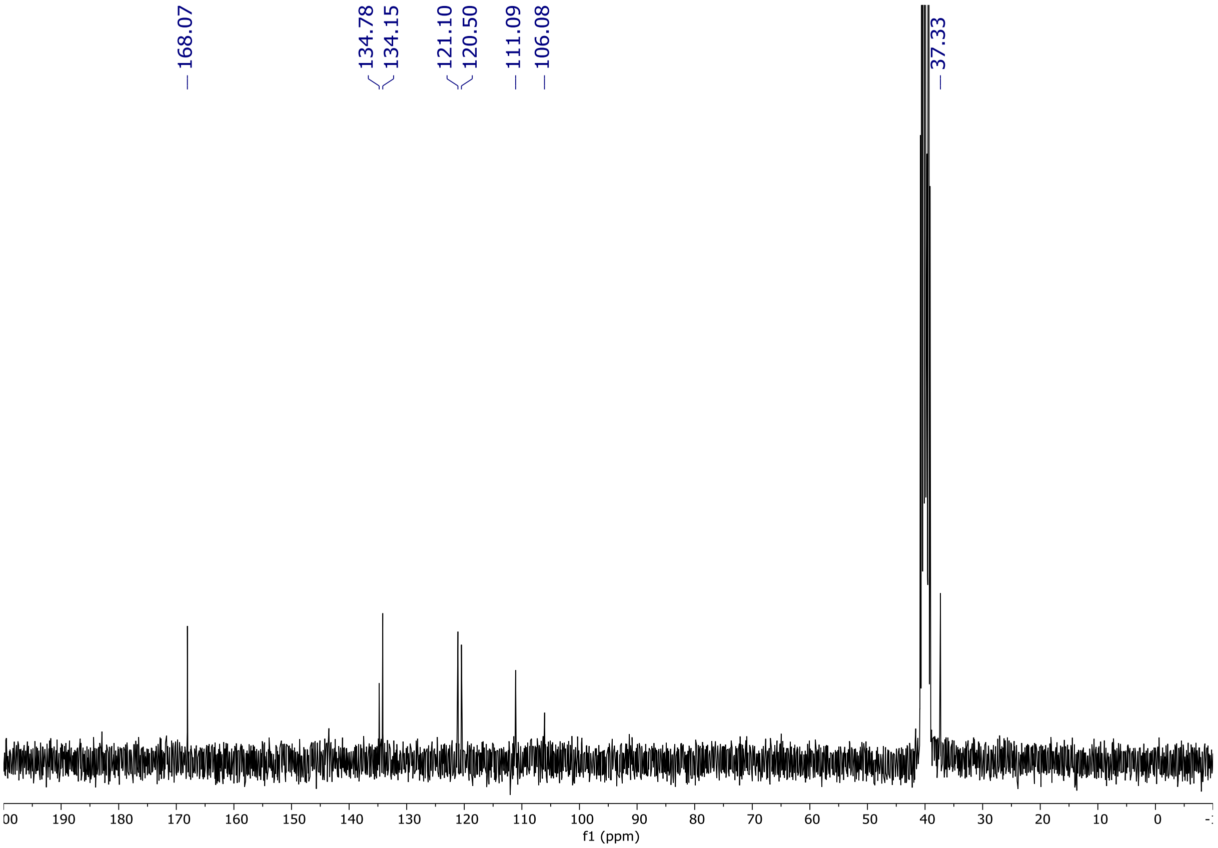

Figure S6. ^{13}C NMR spectrum of compound **5c** in DMSO-d$_6$.

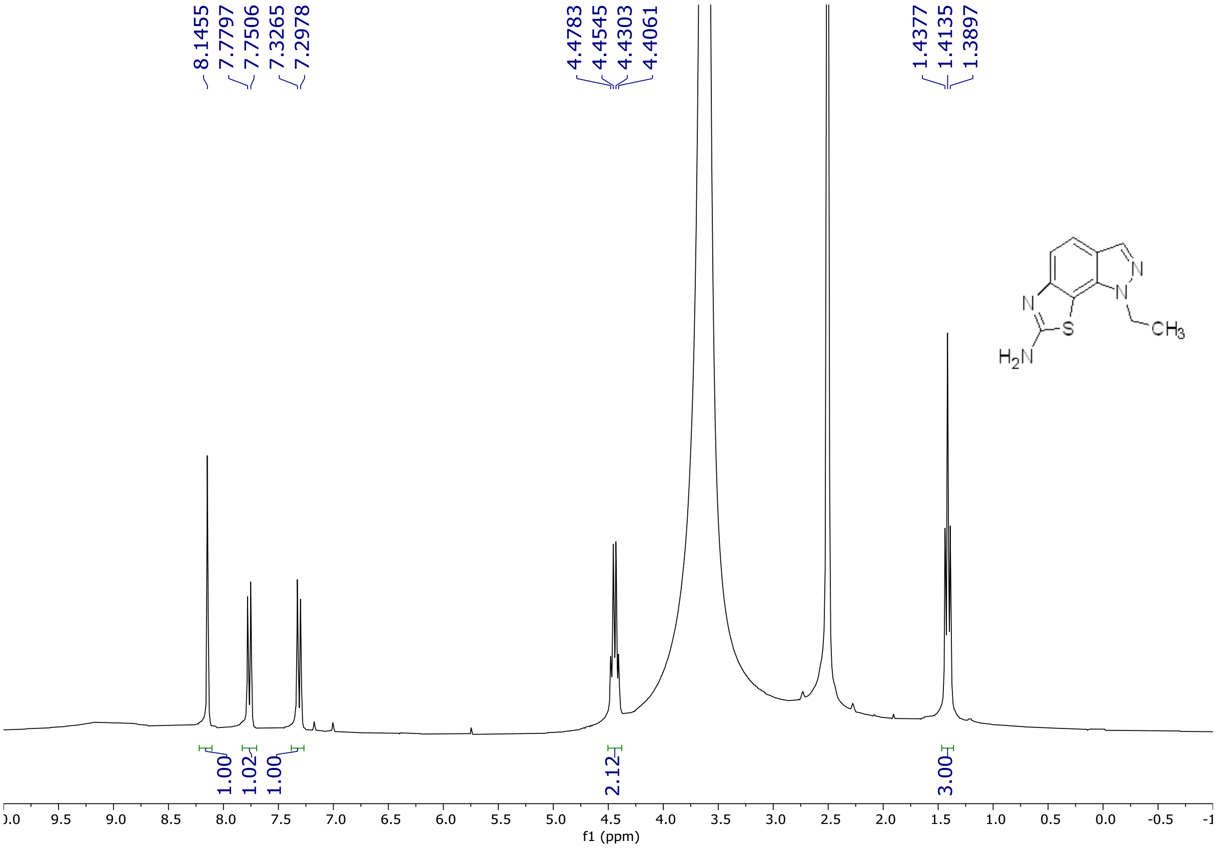

Figure S7. ^{1}H NMR spectrum of compound **5d** in DMSO-d$_6$.

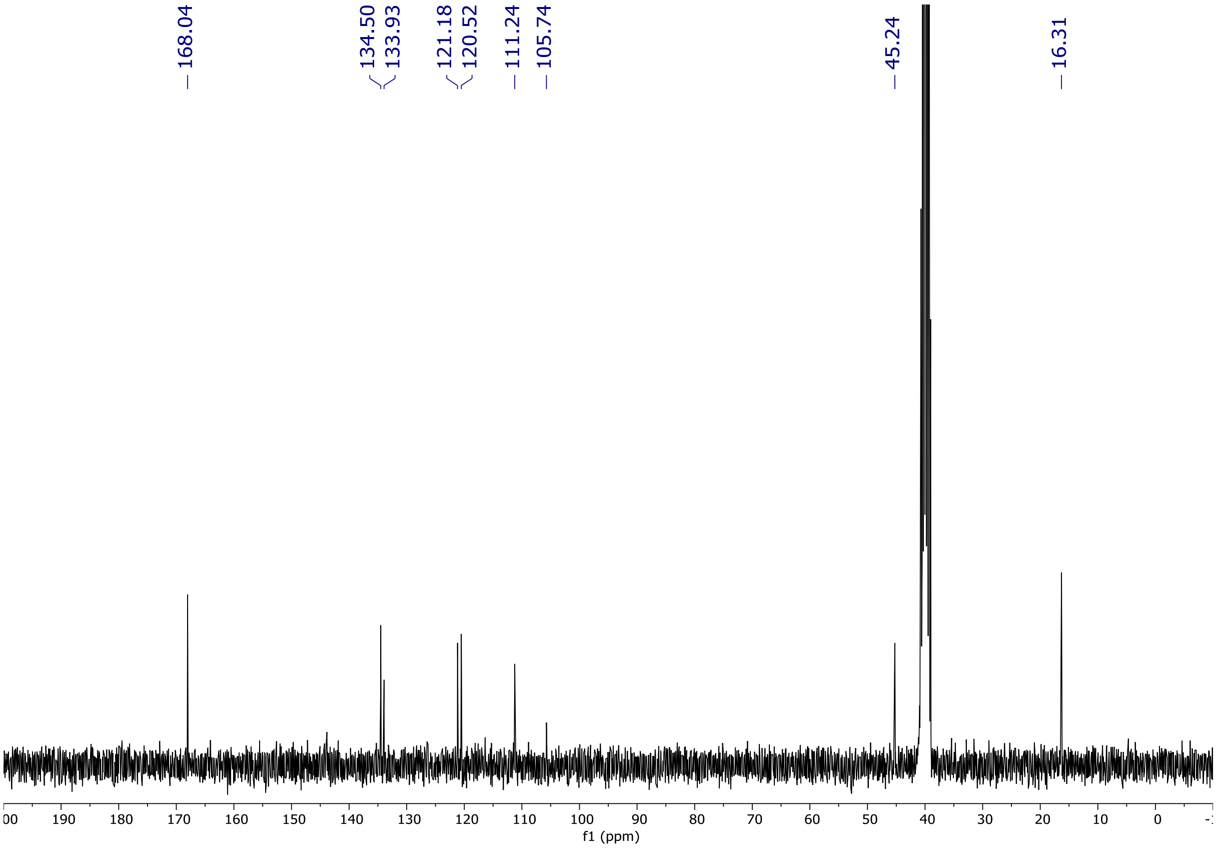

Figure S8. ^{13}C NMR spectrum of compound **5d** in DMSO-d$_6$.

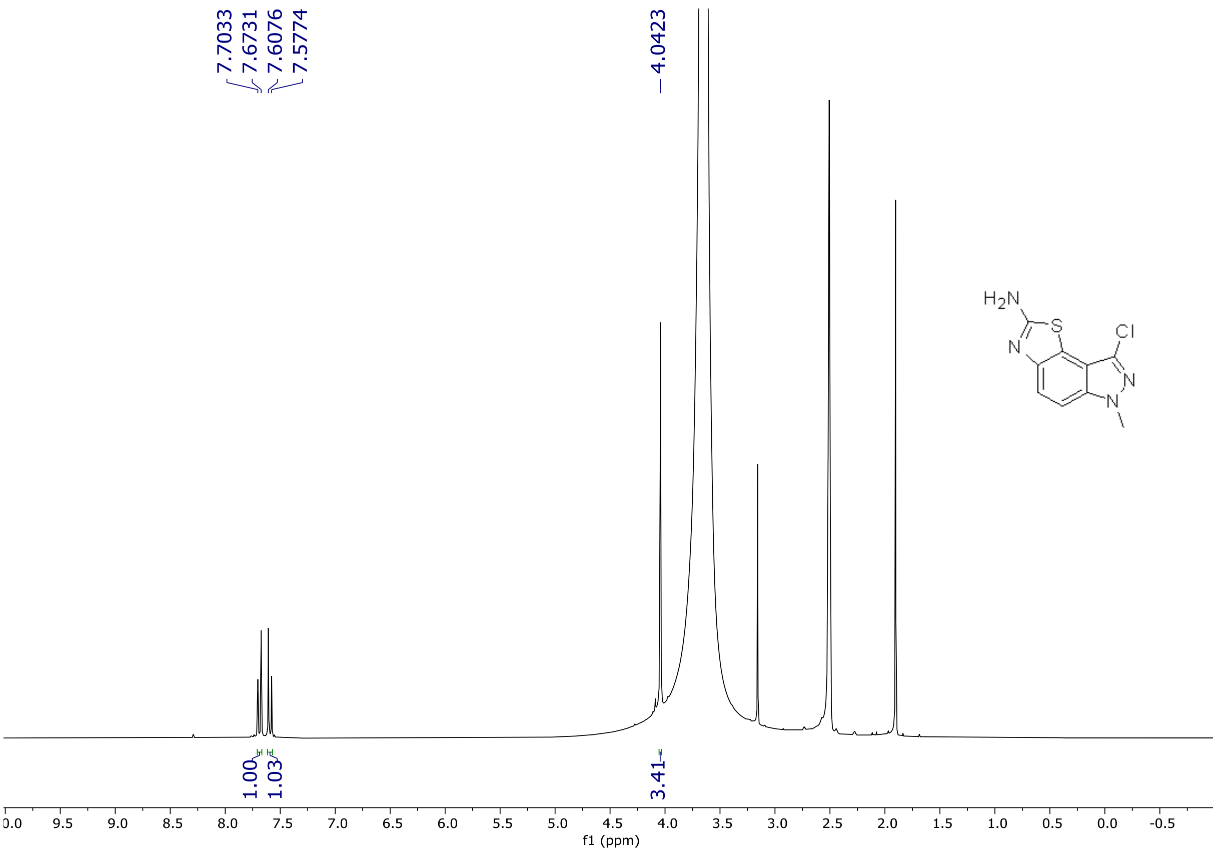

Figure S9. ^{1}H NMR spectrum of compound **5e** in DMSO-d$_6$.

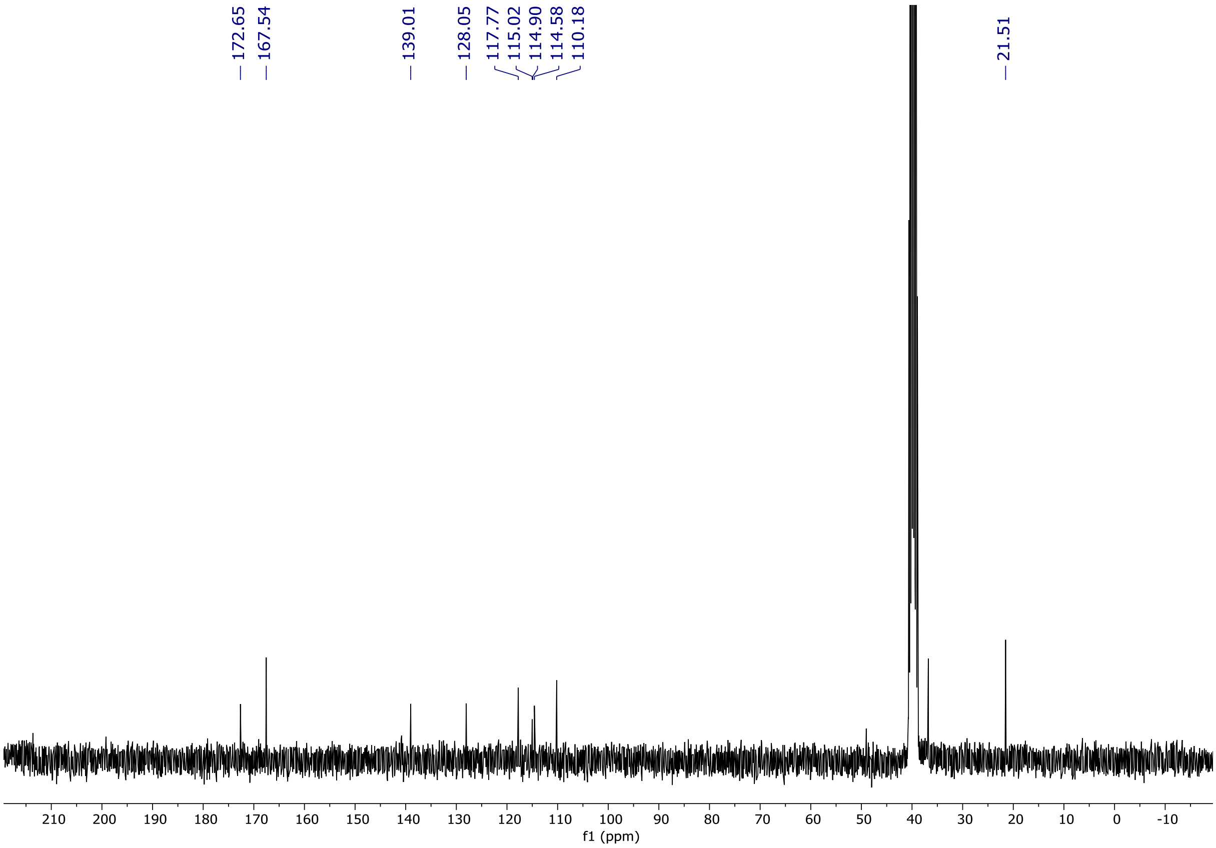

Figure S10. ^{13}C NMR spectrum of compound **5e** in DMSO-d$_6$.

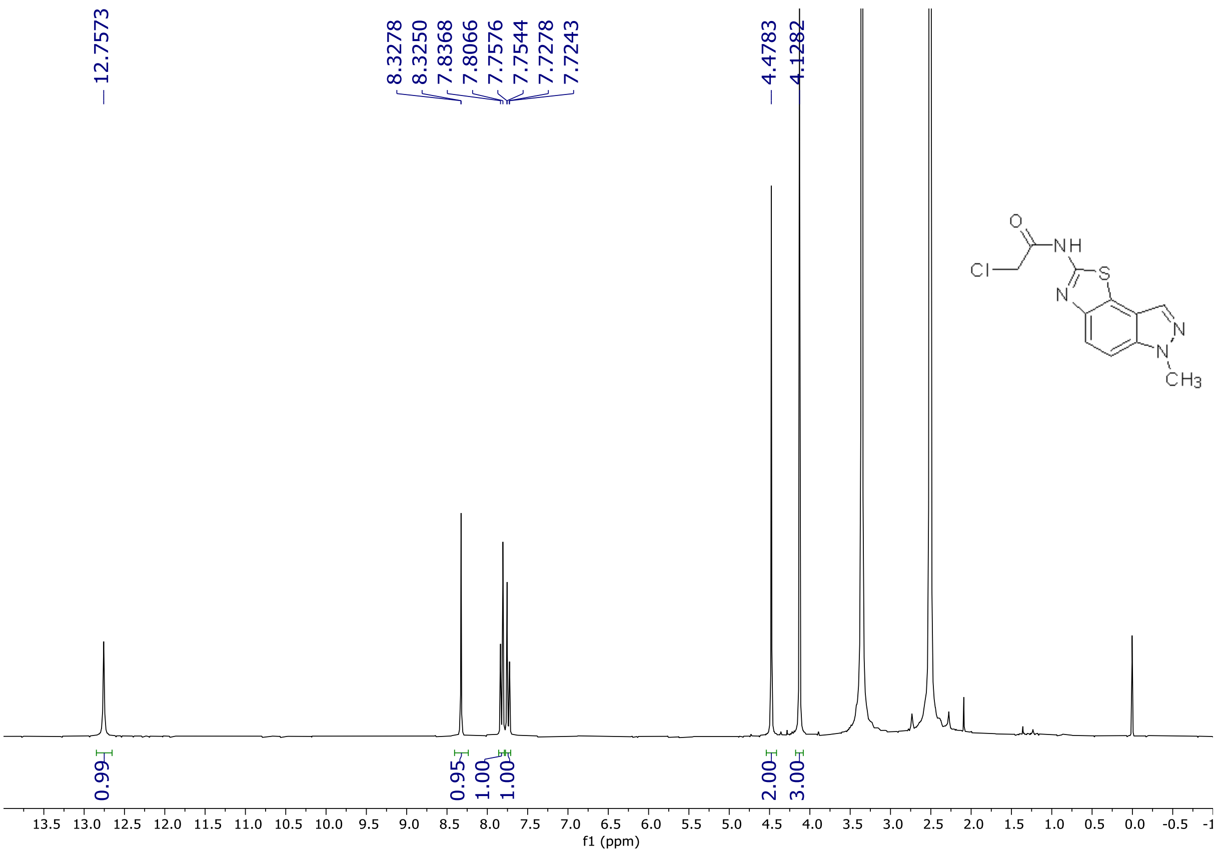

Figure S11. ^{1}H NMR spectrum of compound **6a** in DMSO-d$_6$.

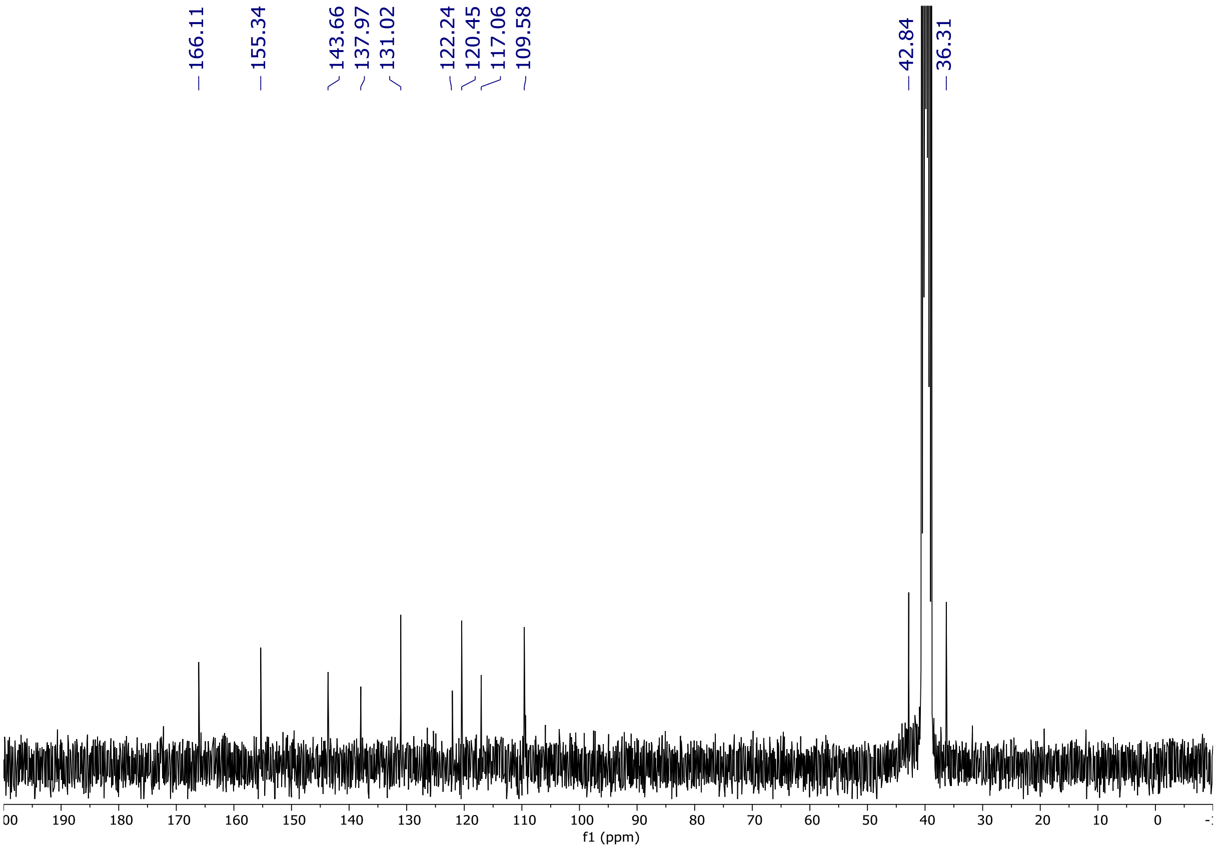

Figure S12. ^{13}C NMR spectrum of compound **6a** in DMSO-d$_6$.

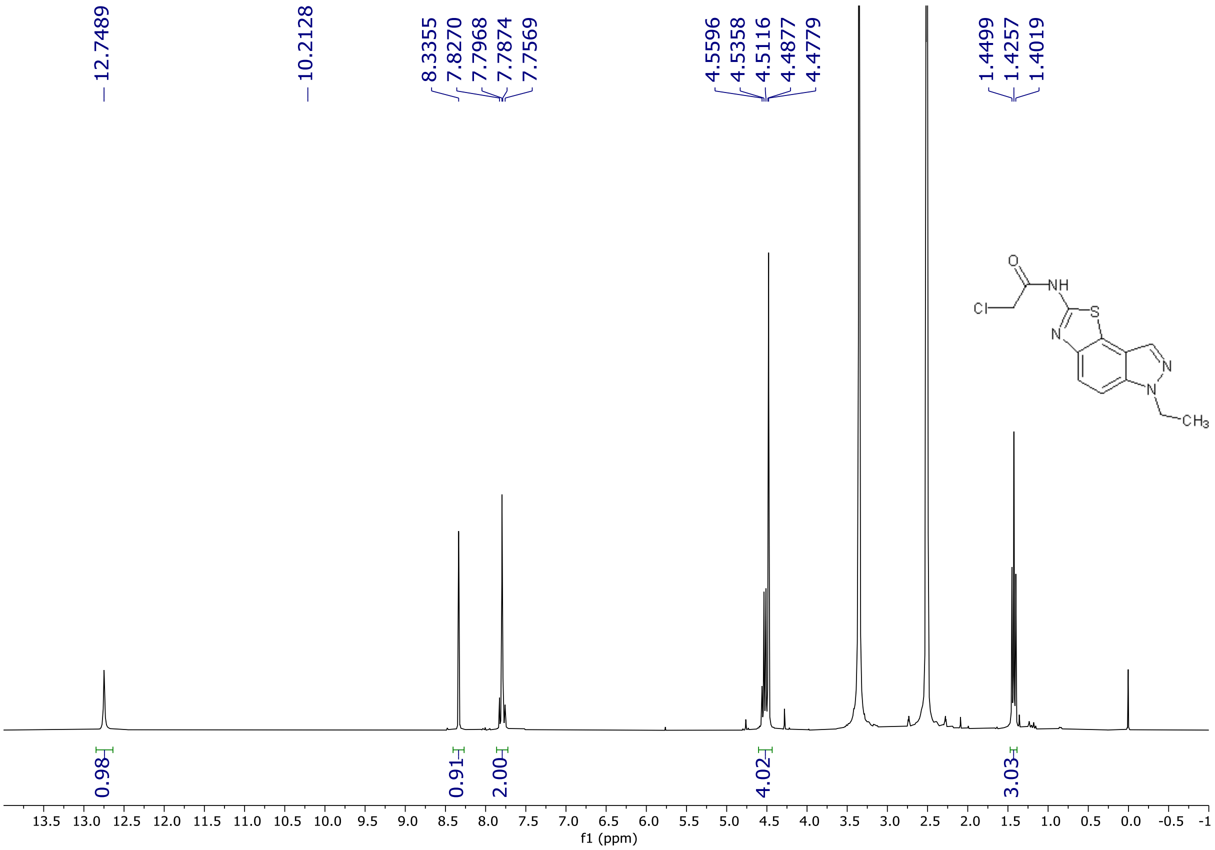

Figure S13. ^{1}H NMR spectrum of compound **6b** in DMSO-d_6.

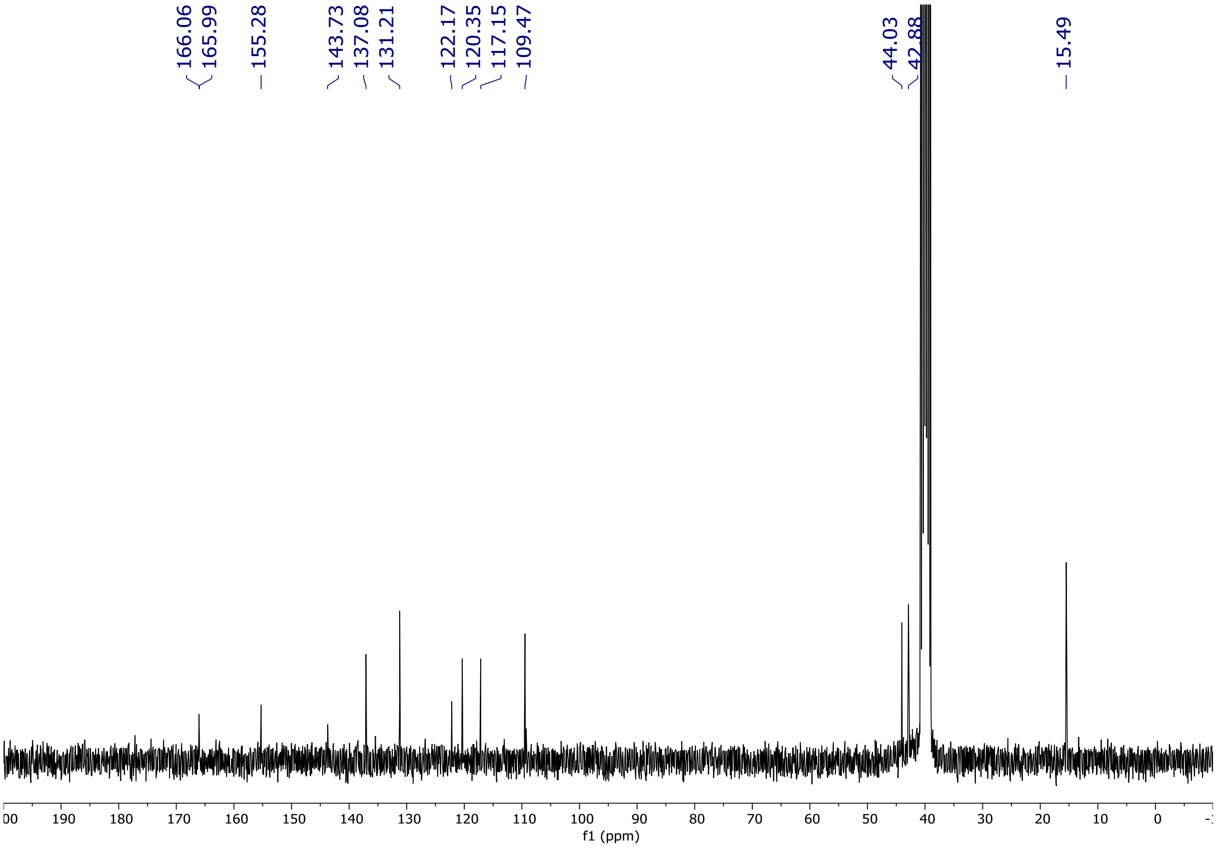

Figure S14. ^{13}C NMR spectrum of compound **6b** in DMSO-d_6.

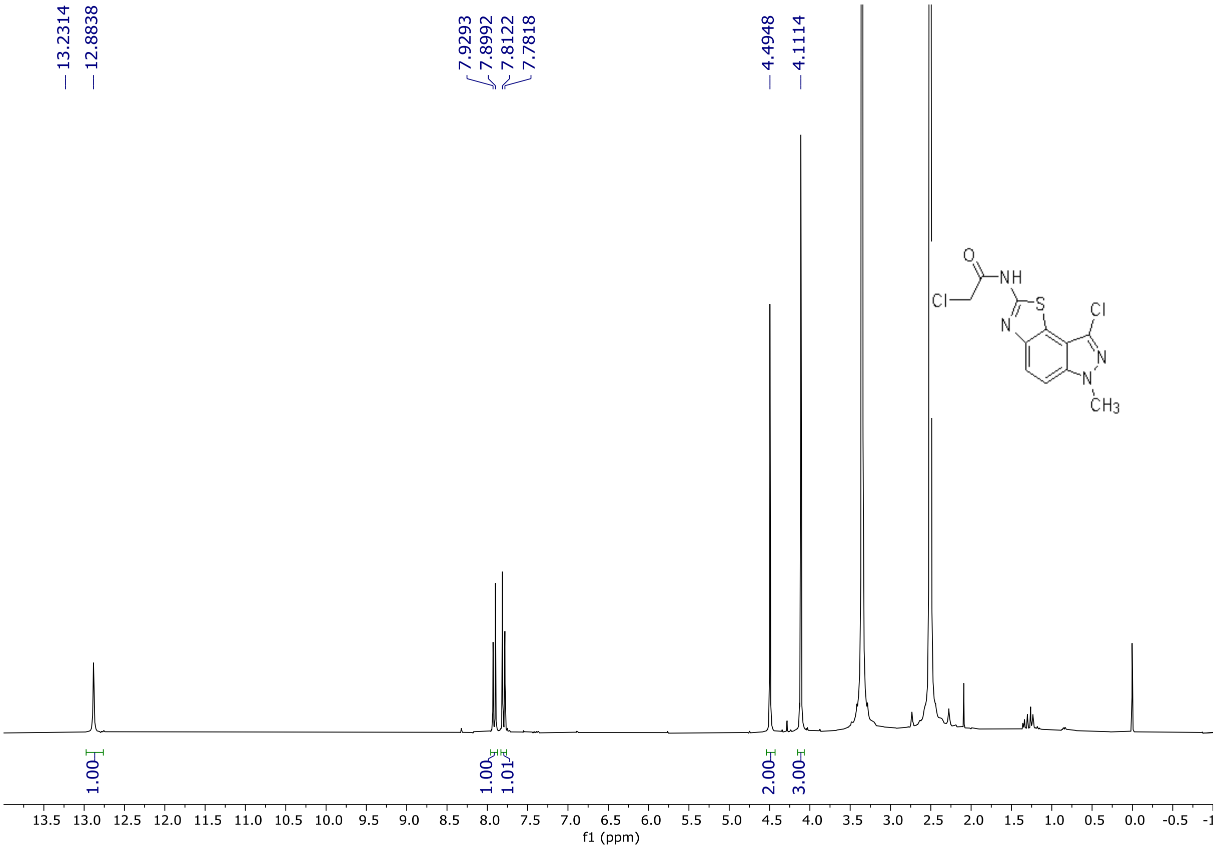

Figure S15. ^{13}C NMR spectrum of compound **6c** in DMSO-d_6.

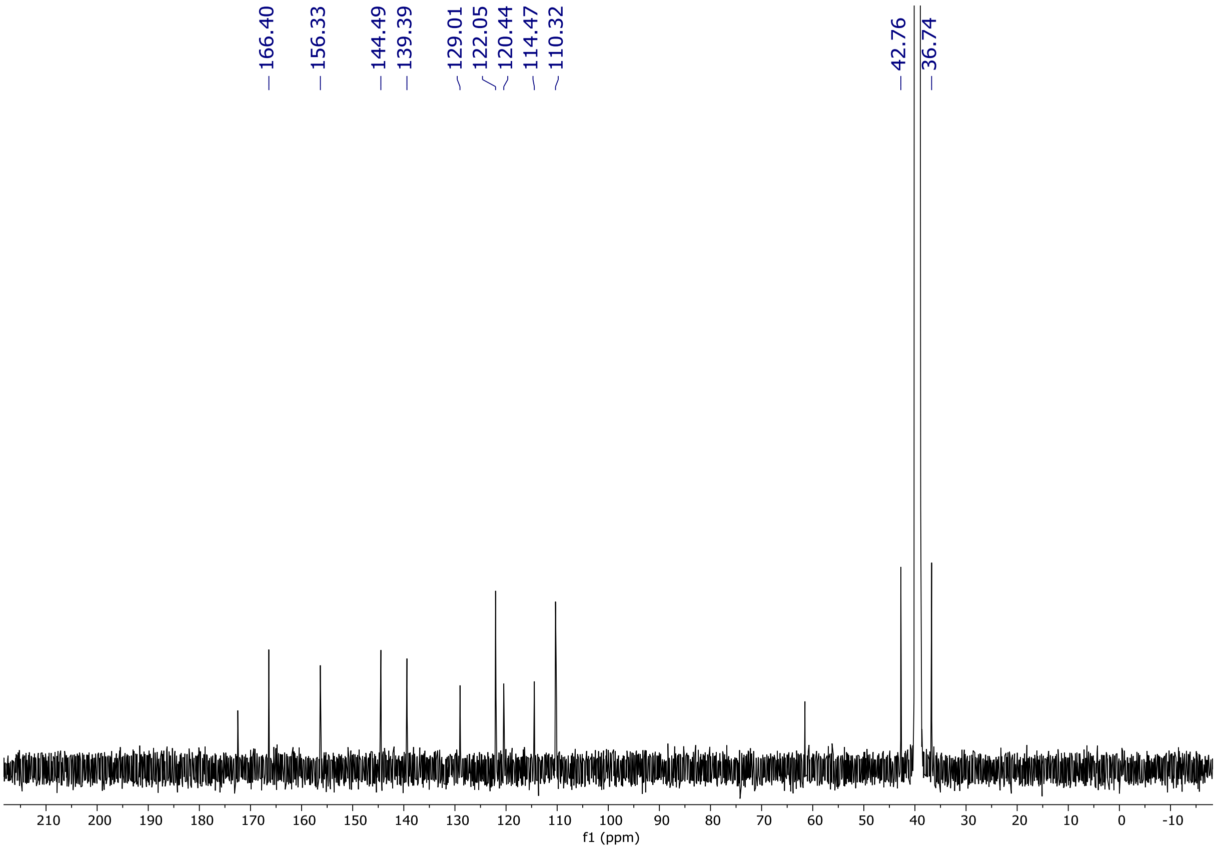

Figure S16. ^{13}C NMR spectrum of compound **6c** in DMSO-d_6.

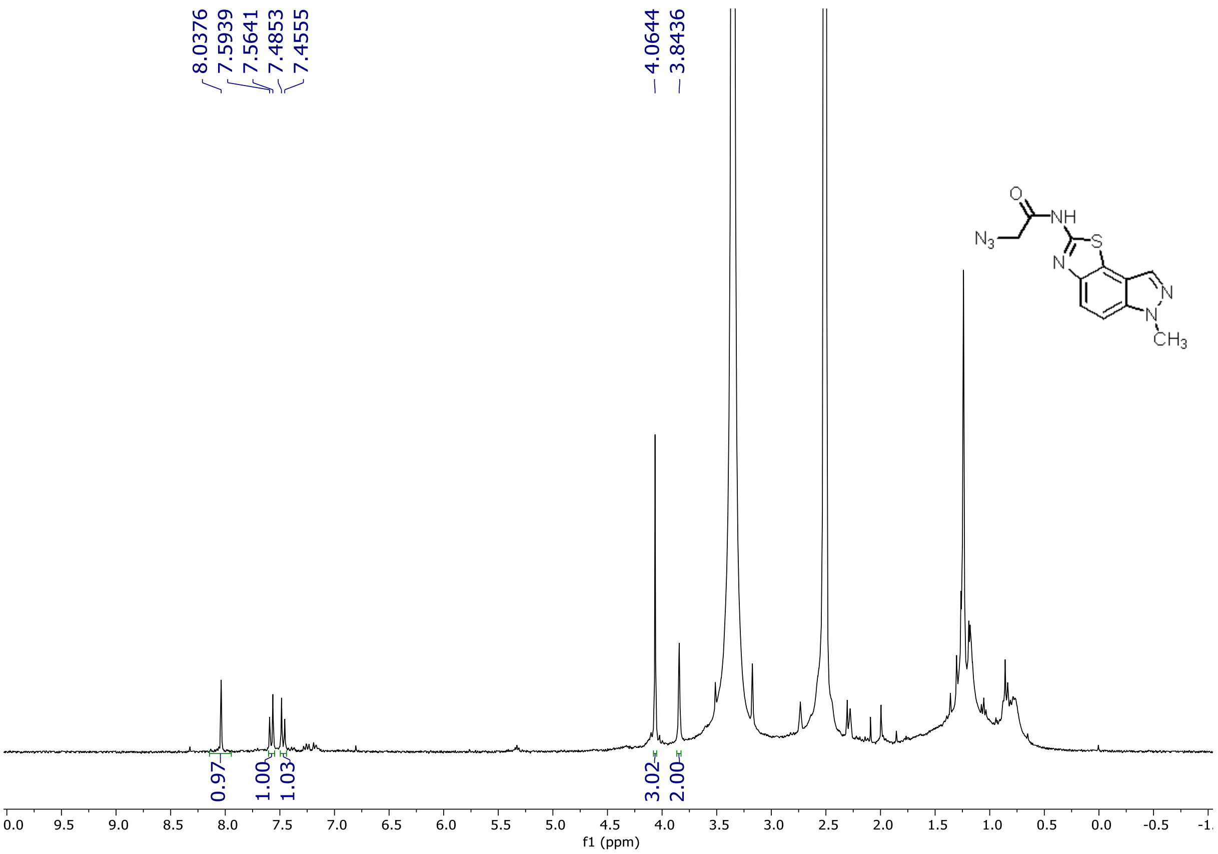

Figure S17. ^{1}H NMR spectrum of compound **7a** in DMSO-d_6.

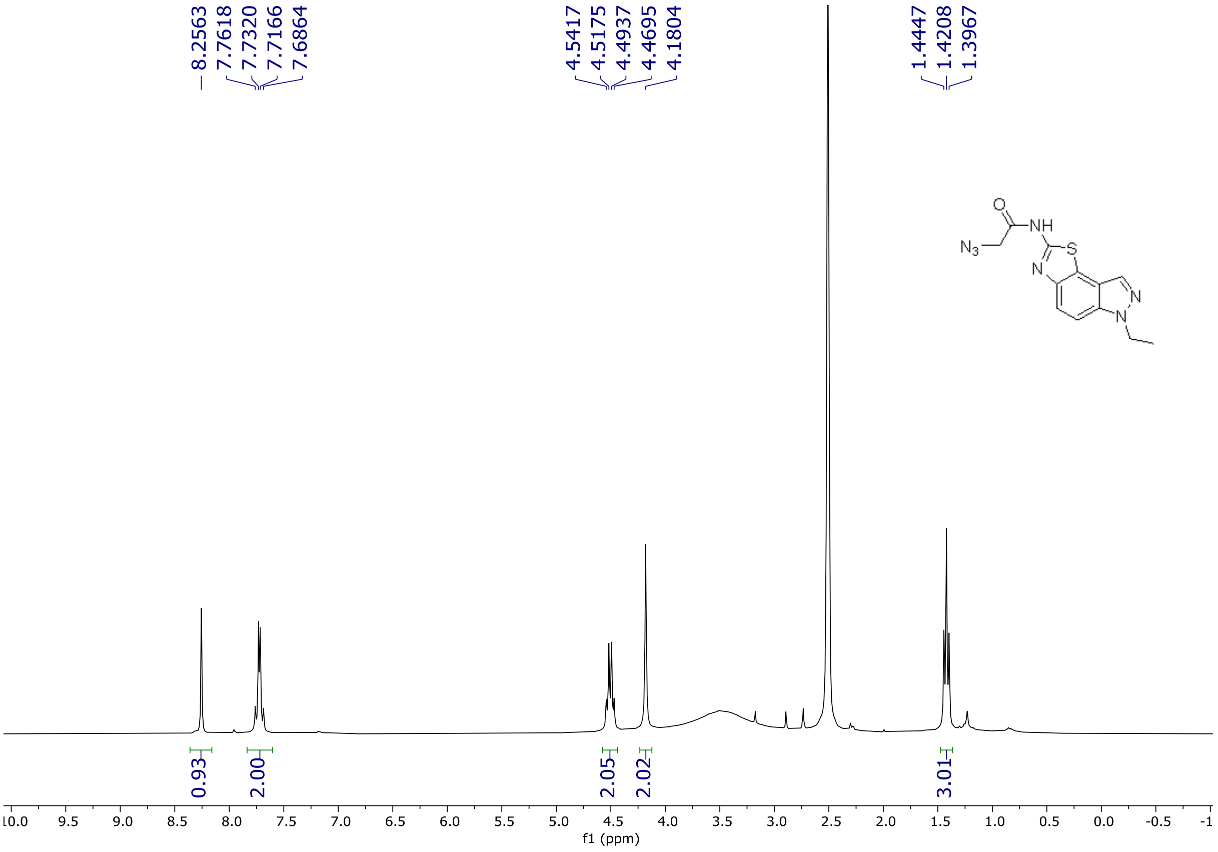

Figure S18. ^{1}H NMR spectrum of compound **7b** in DMSO-d_6.

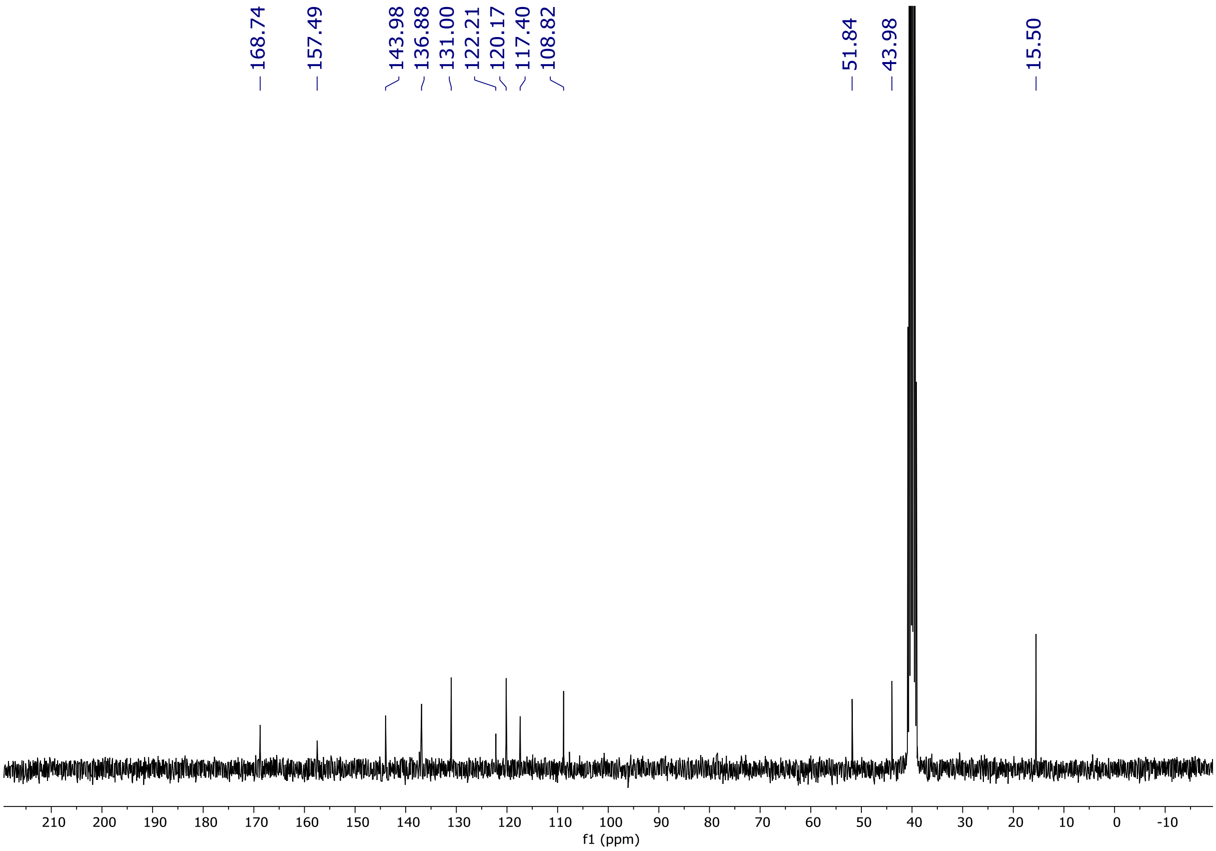

Figure S19. ^{13}C NMR spectrum of compound **7b** in DMSO-d$_6$.

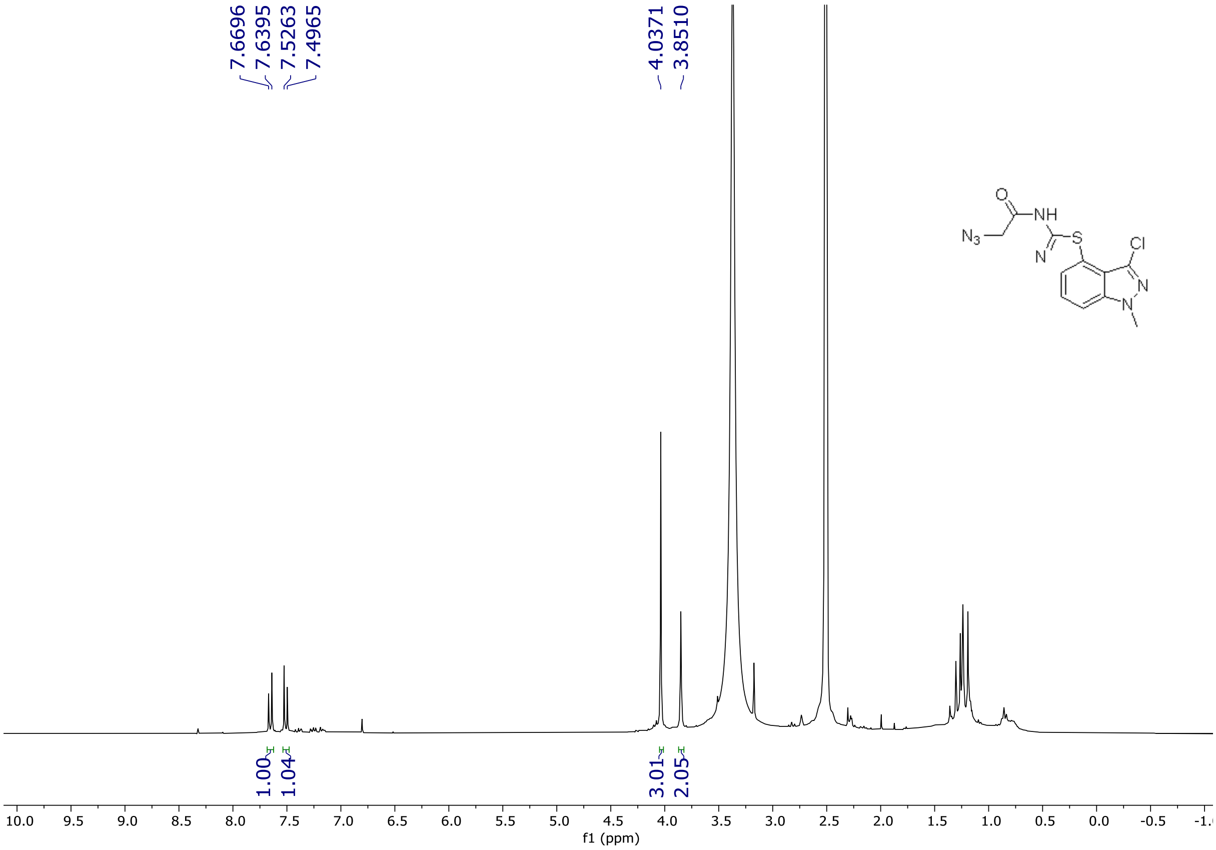

Figure S20. ^{1}H NMR spectrum of compound **7c** in DMSO-d$_6$.

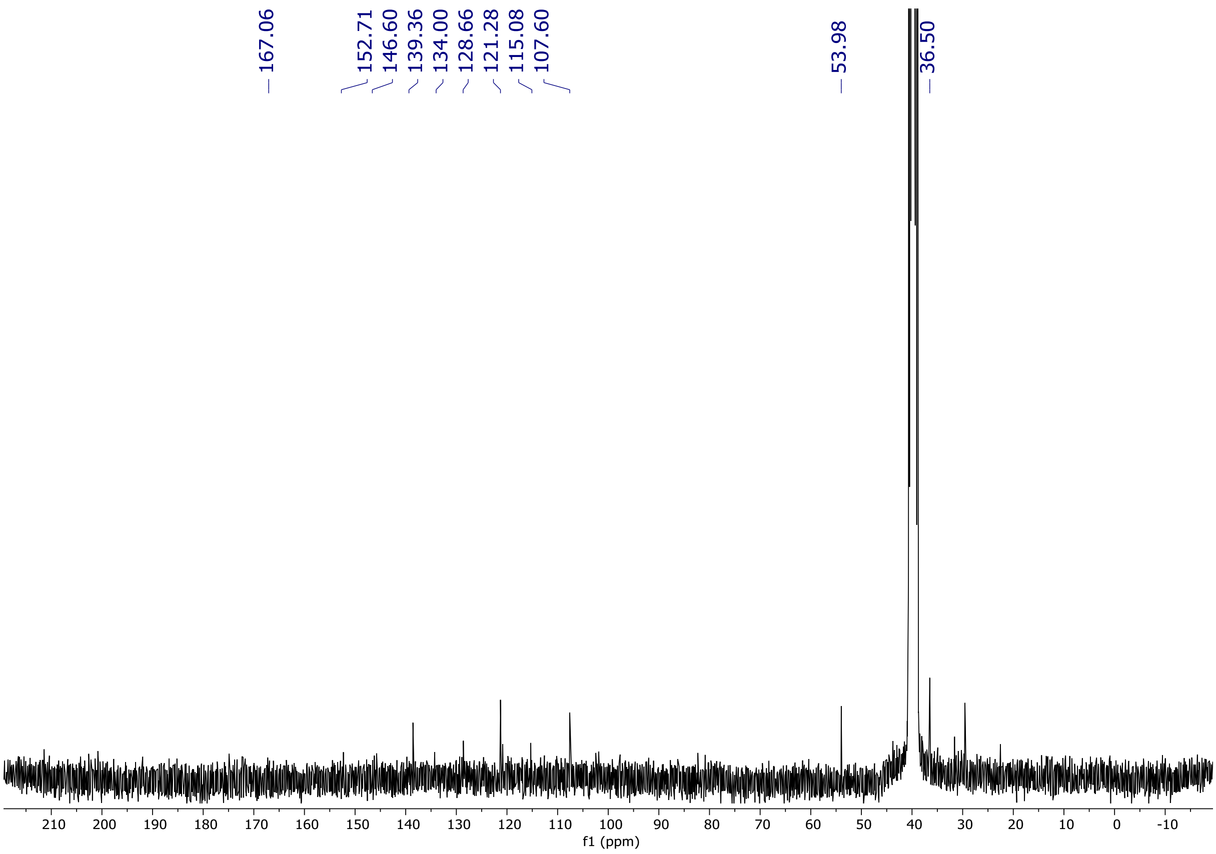

Figure S21. ^{13}C NMR spectrum of compound **7c** in DMSO-d_6.

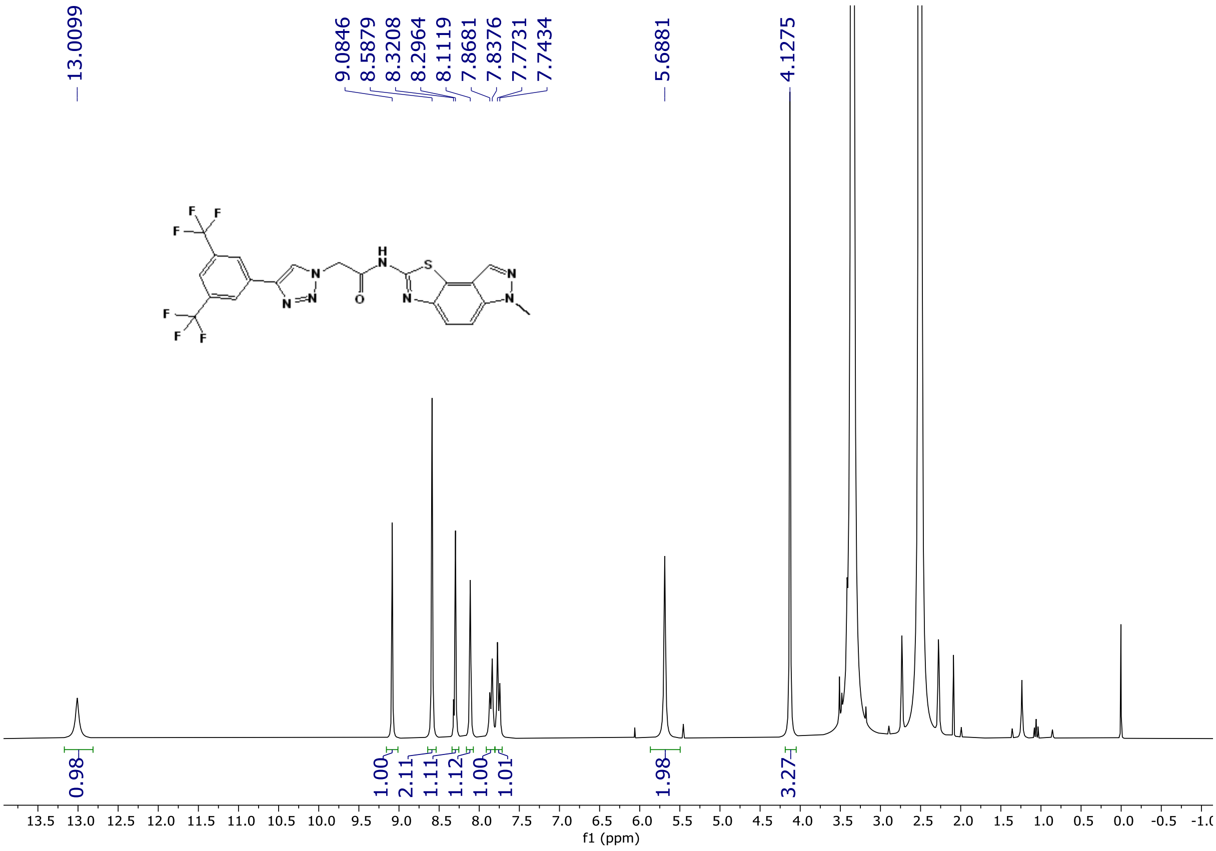

Figure S22. ^{1}H NMR spectrum of compound **TI45a** in DMSO-d_6.

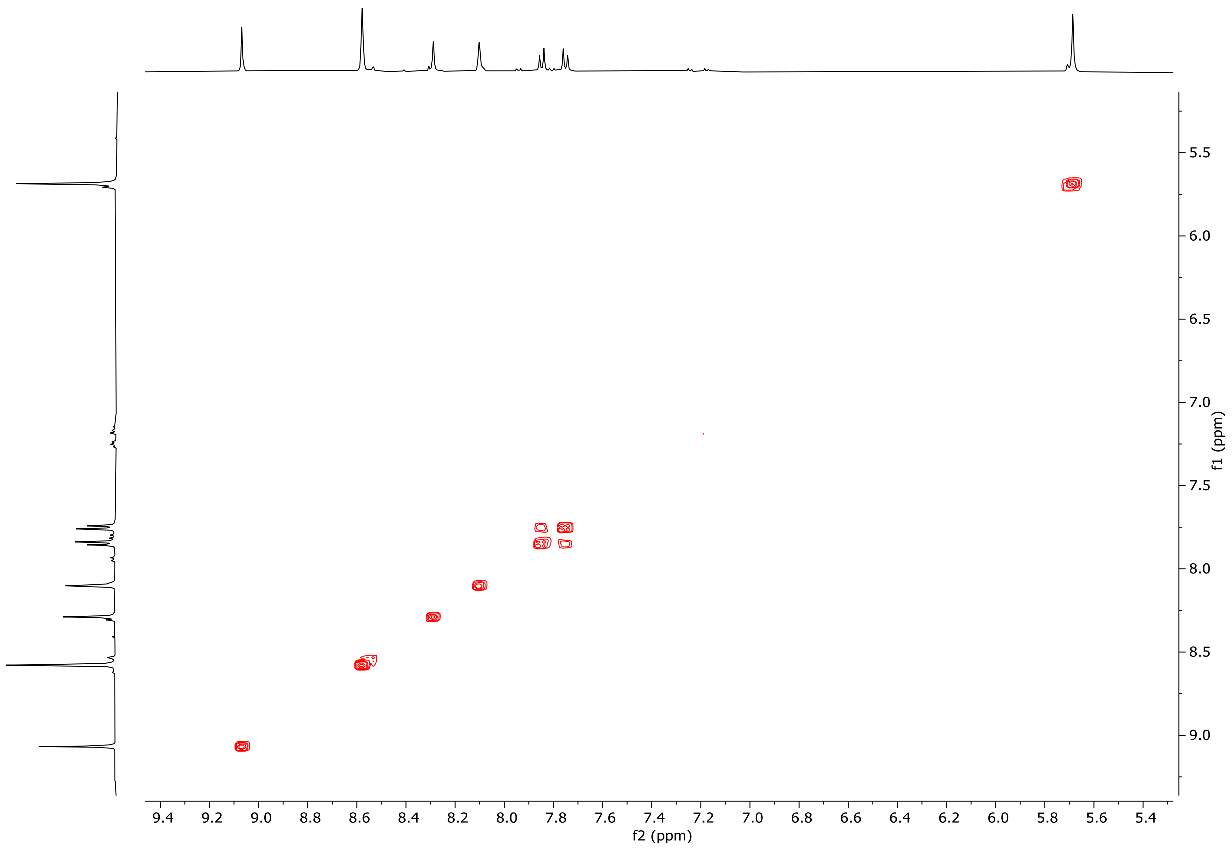

Figure S23. ^{1}H/^{1}H COSY NMR spectrum of compound **TI45a** in DMSO-d_6.

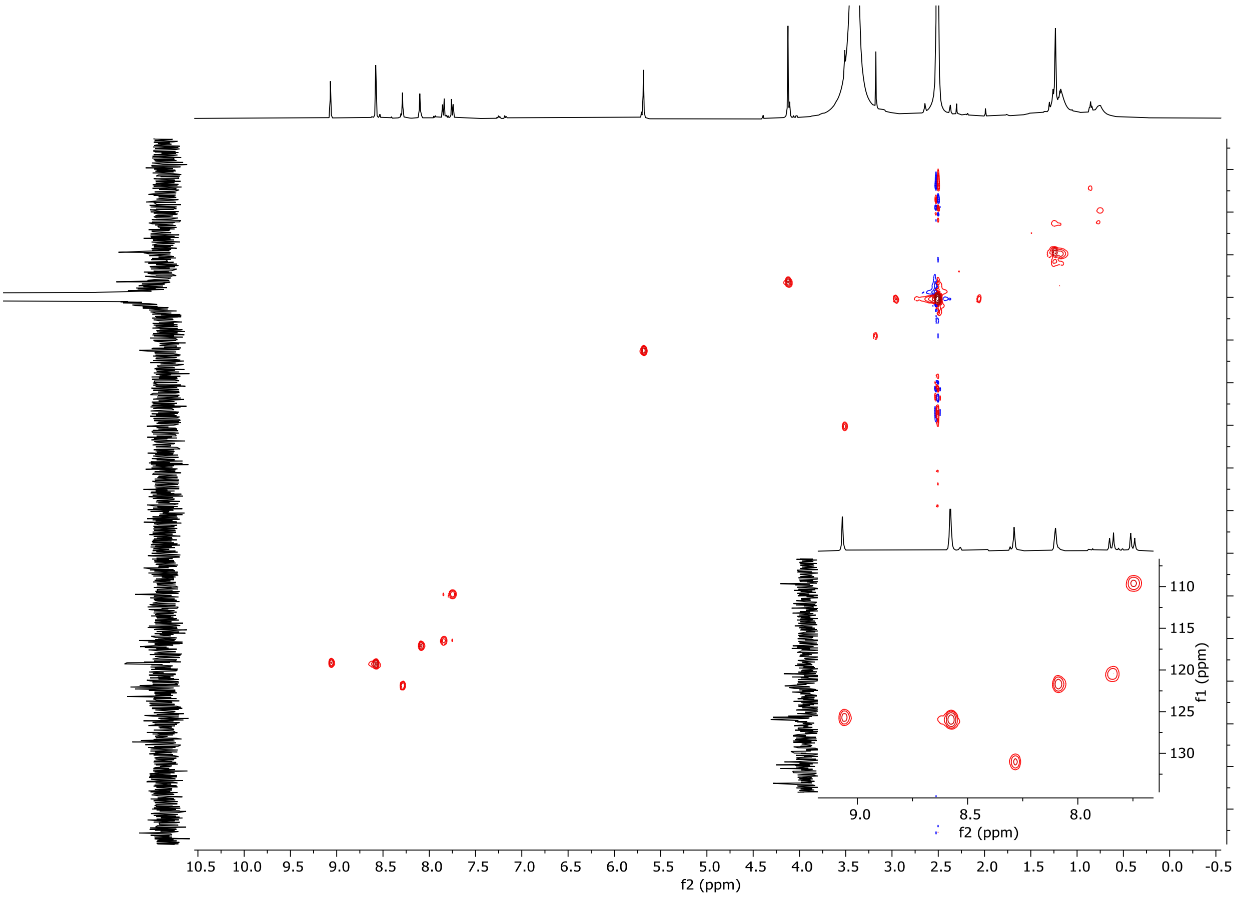

Figure S24. $^{1}H/^{13}C$ HSQC NMR spectrum of compound **TI45a** in DMSO-d_6.

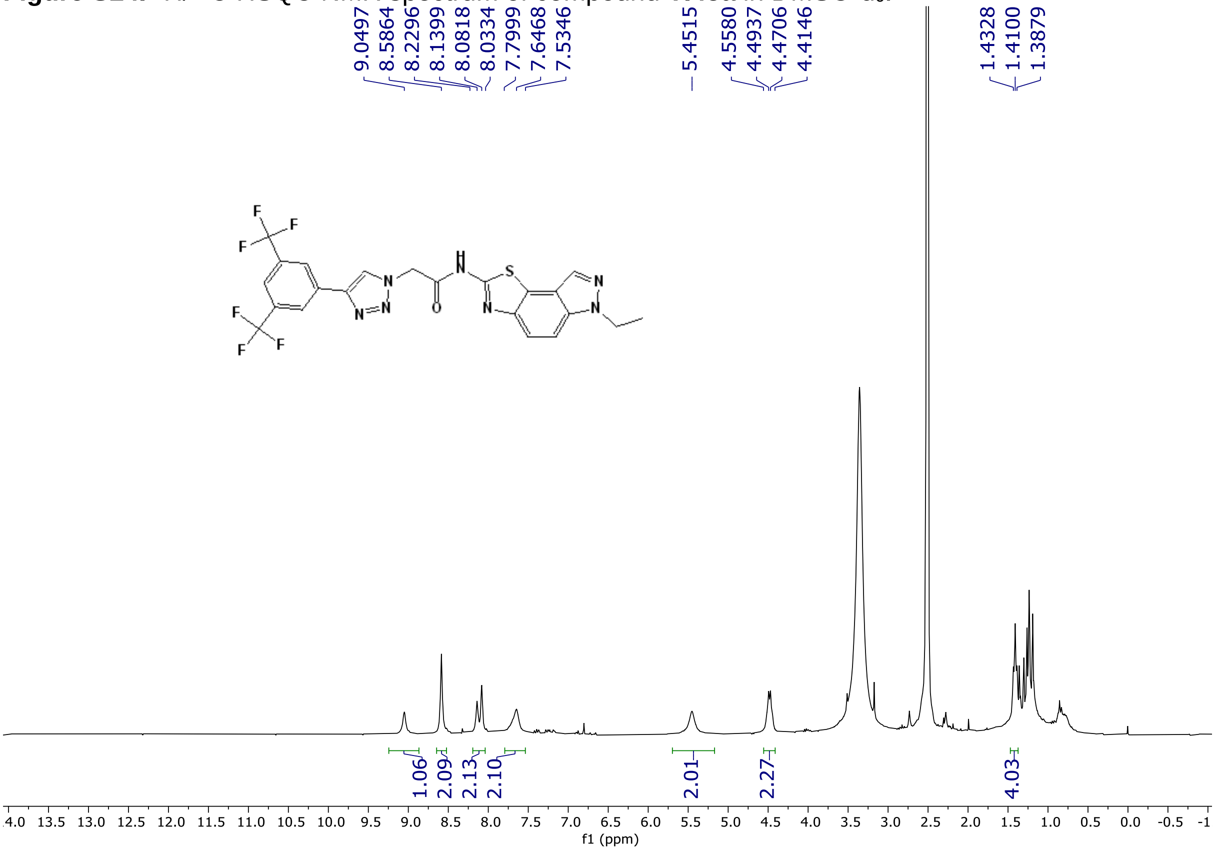

Figure S25. ^{1}H NMR spectrum of compound **TI45b** in DMSO-d_6.

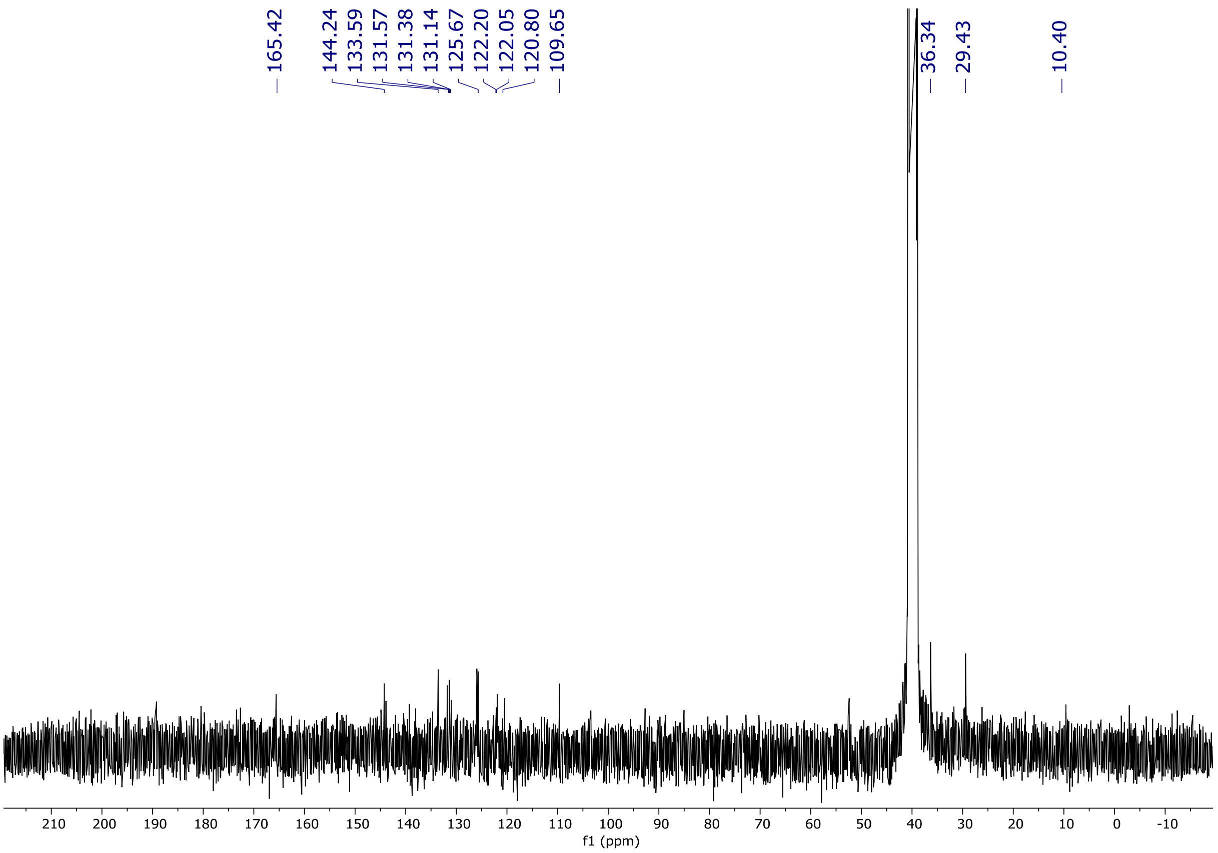

Figure S26. ^{13}C NMR spectrum of compound **TI45b** in DMSO-d_6.

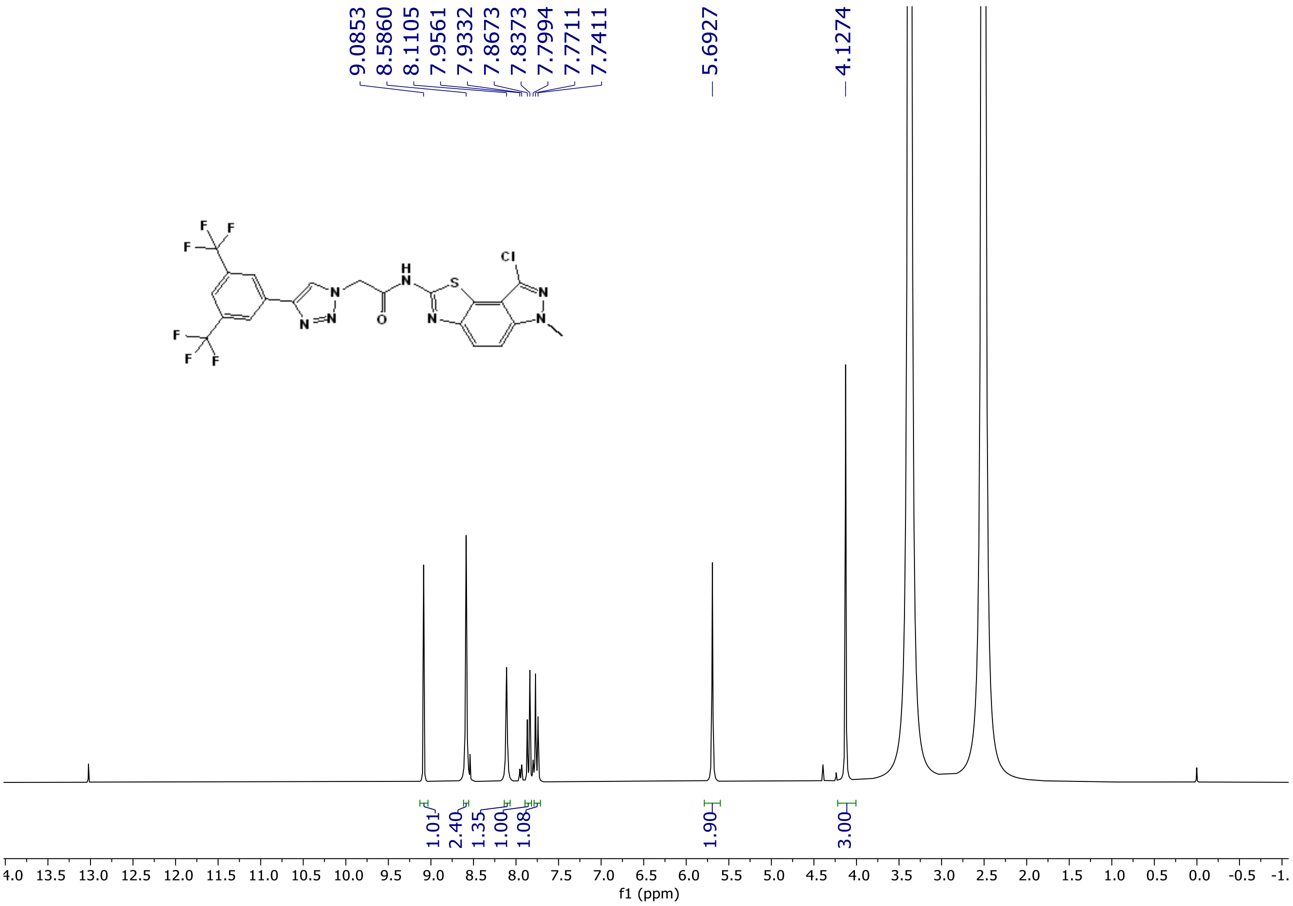

Figure S27. ^{1}H NMR spectrum of compound **TI45c** in DMSO-d6.

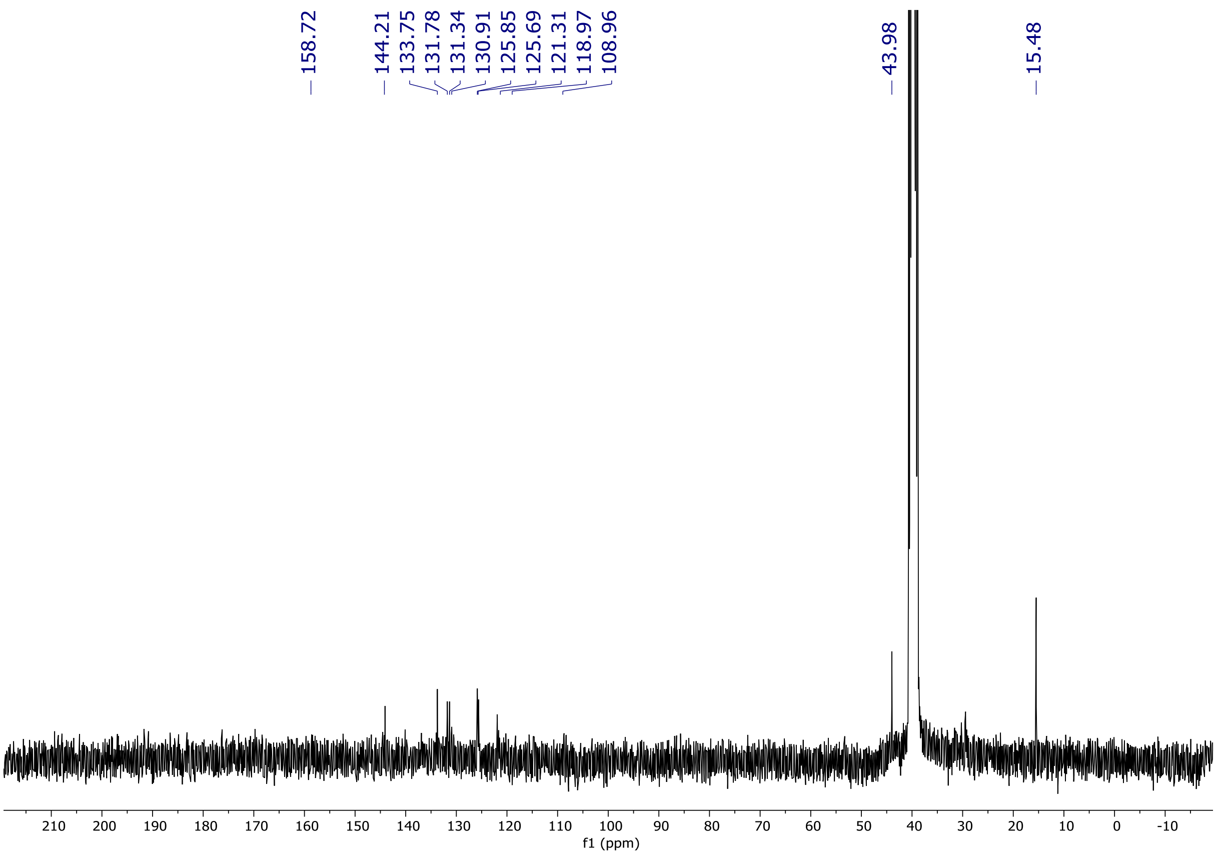

Figure S28. ^{1}H NMR spectrum of compound **TI45c** in DMSO-d6.

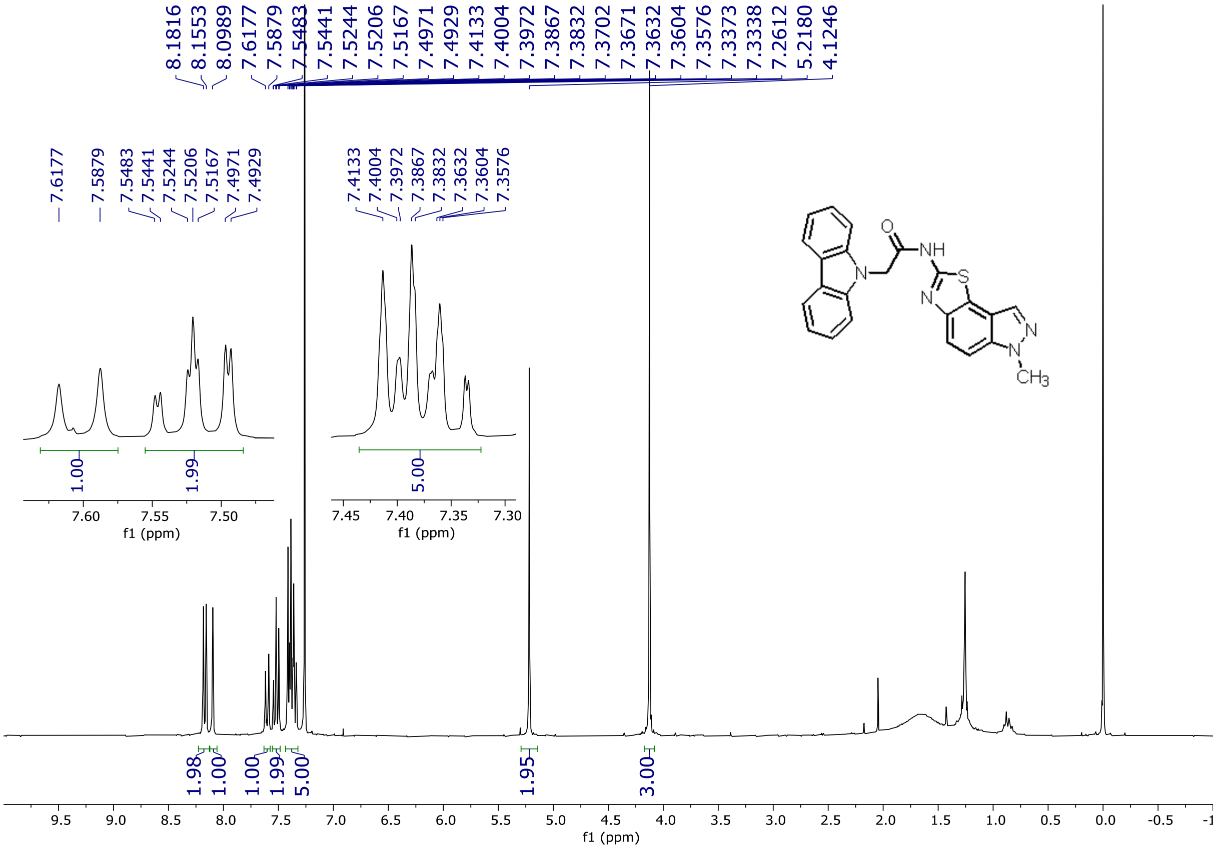

Figure S29. ^{1}H NMR spectrum of compound **TI58a** in $CDCl_3$.

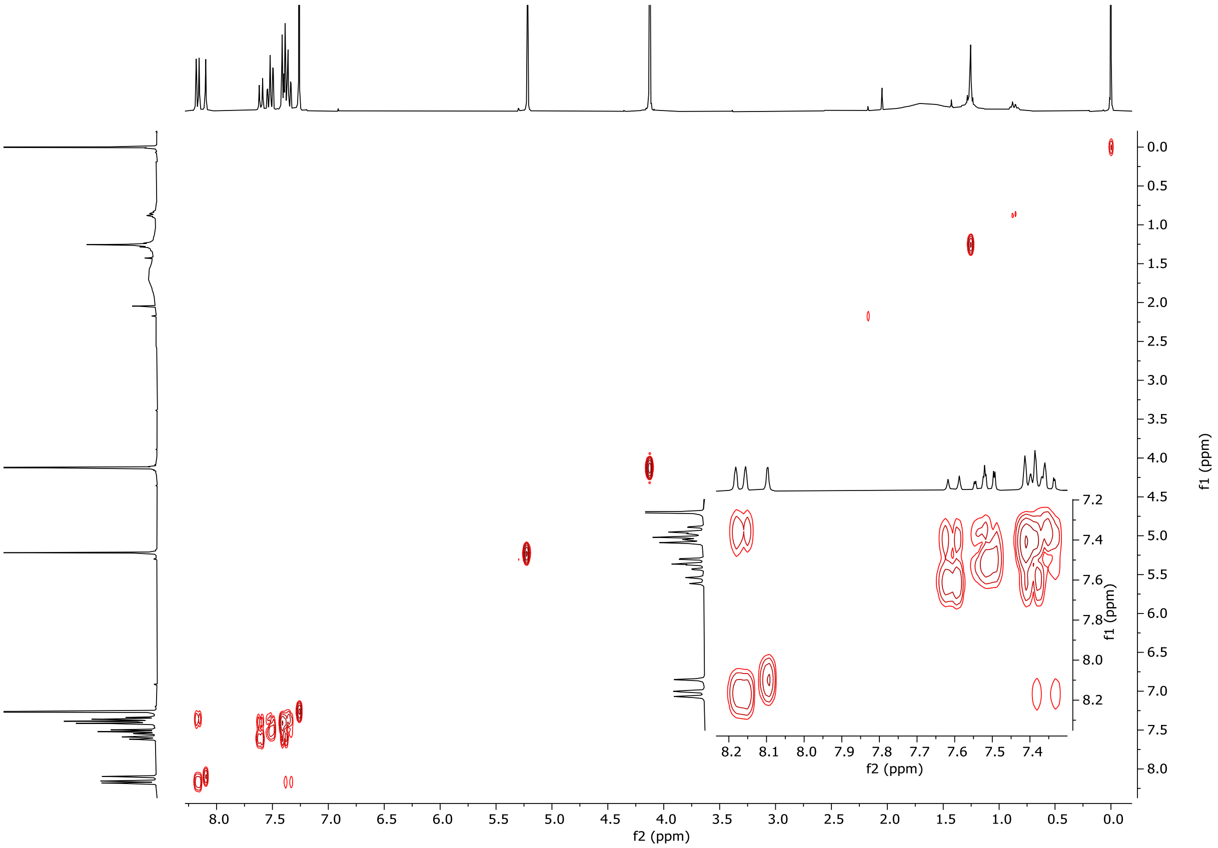

Figure S30. $^{1}H/^{1}H$ COSY spectrum of compound **TI58a** in $CDCl_3$.

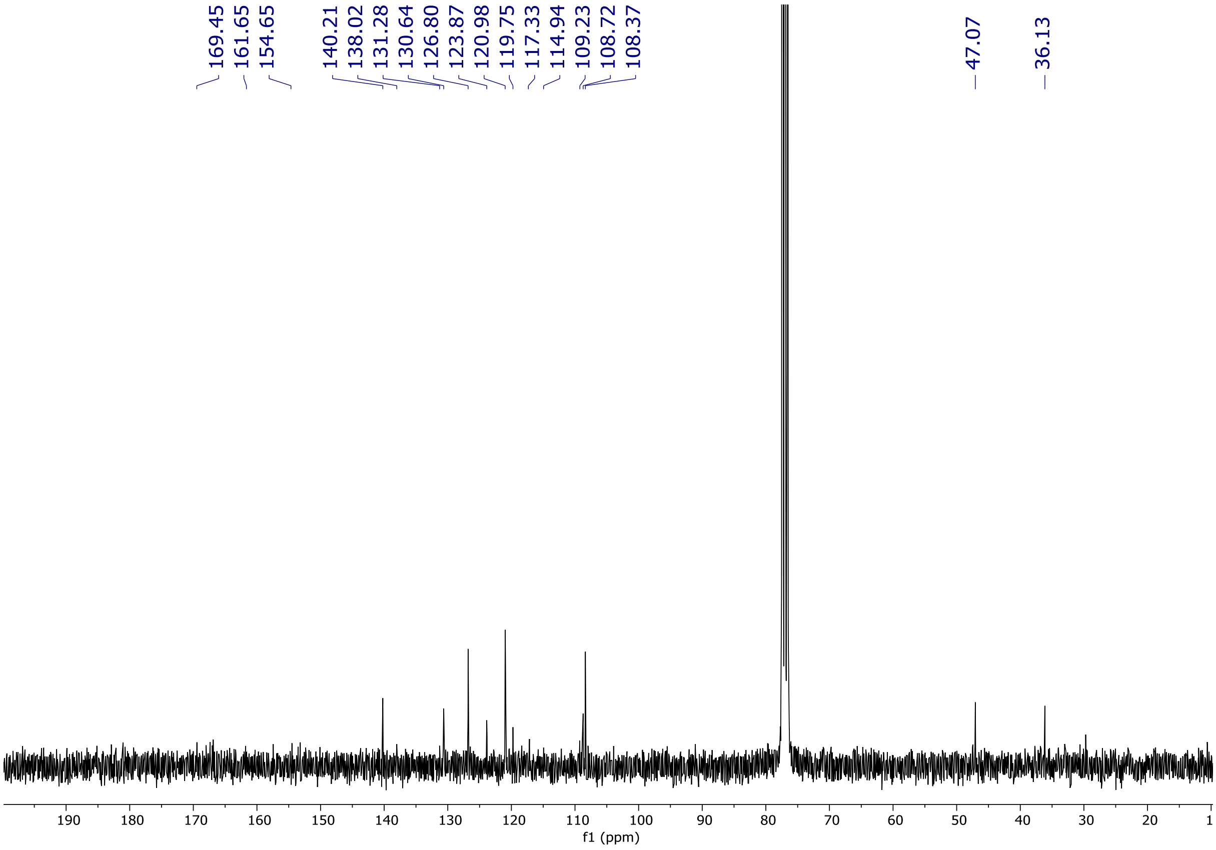

Figure S31. ^{13}C NMR spectrum of compound **TI58a** in $CDCl_3$.

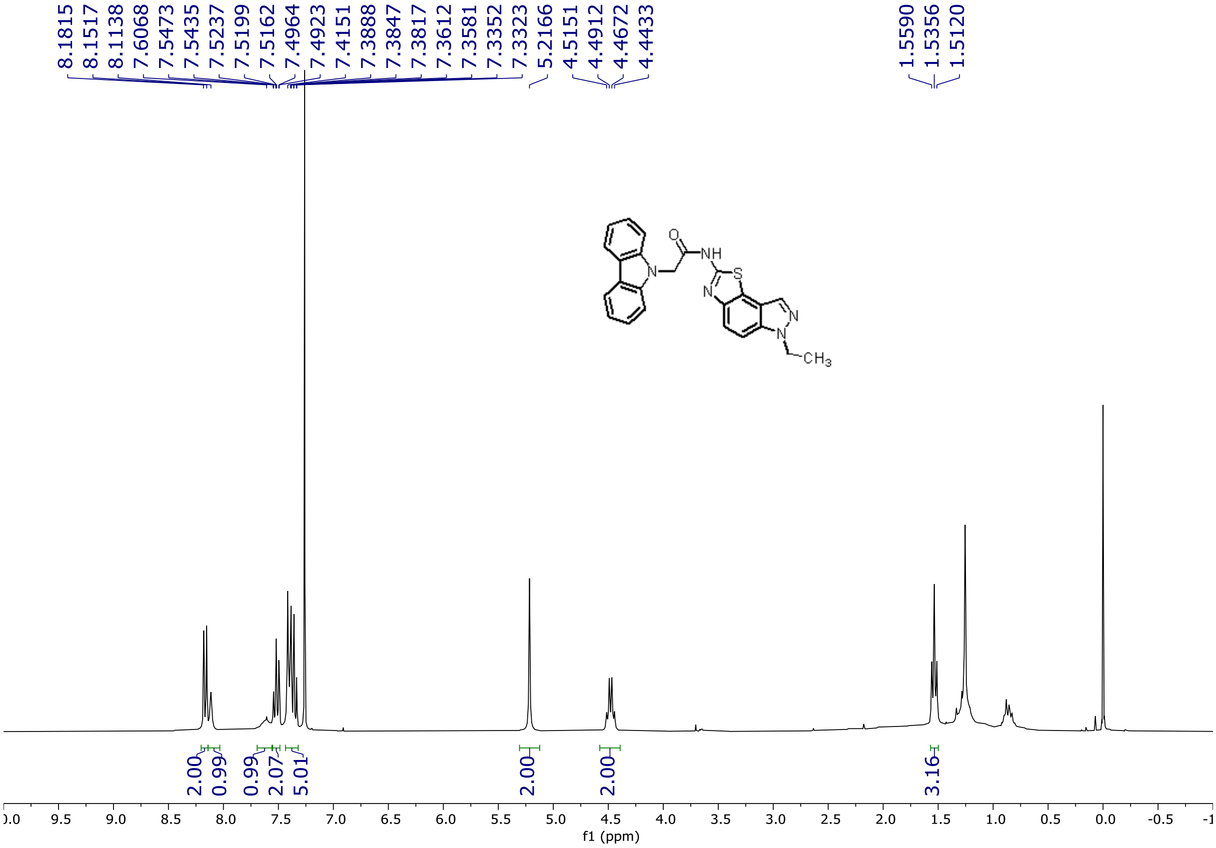

Figure S32. ^{1}H NMR spectrum of compound **TI58b** in $CDCl_3$.

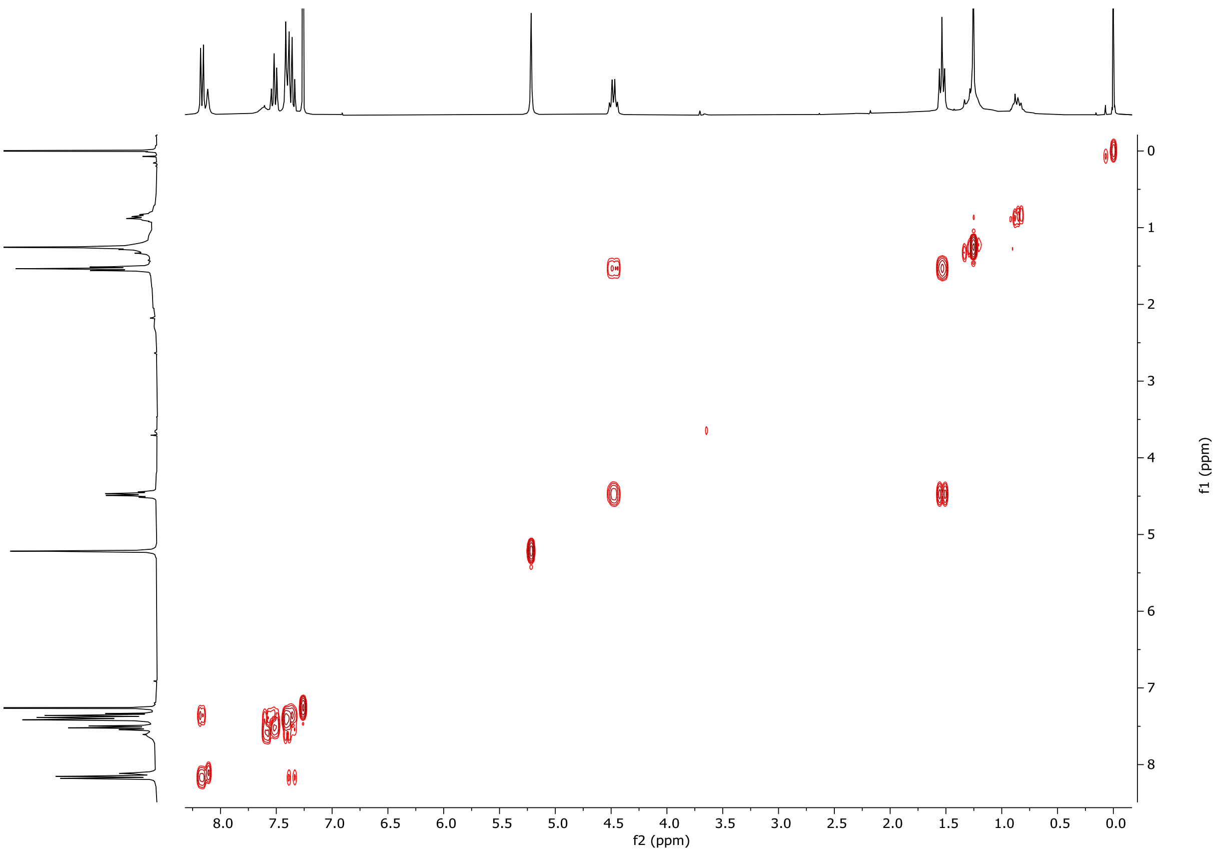

Figure S33. $^{1}H/^{1}H$ COSY spectrum of compound **TI58b** in $CDCl_3$.

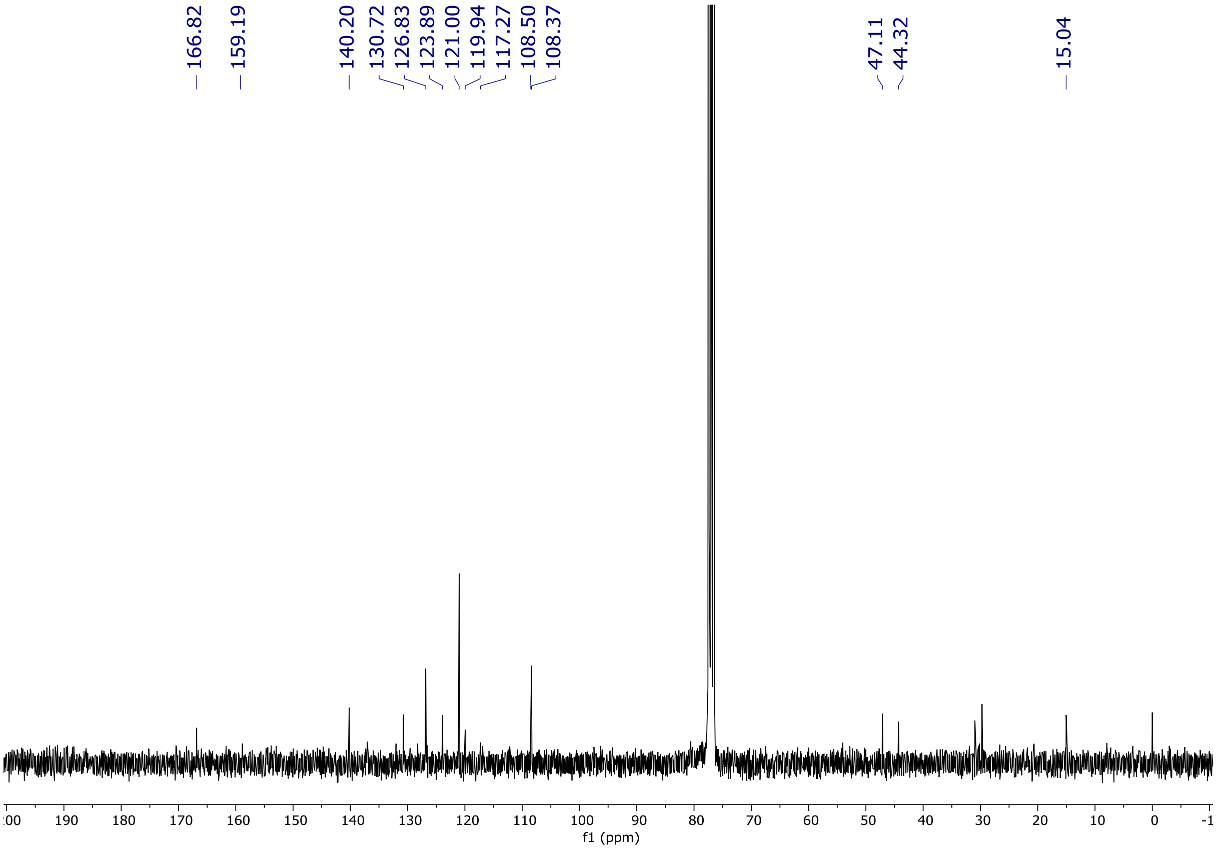

Figure S34. ^{13}C NMR spectrum of compound **TI58b** in $CDCl_3$.

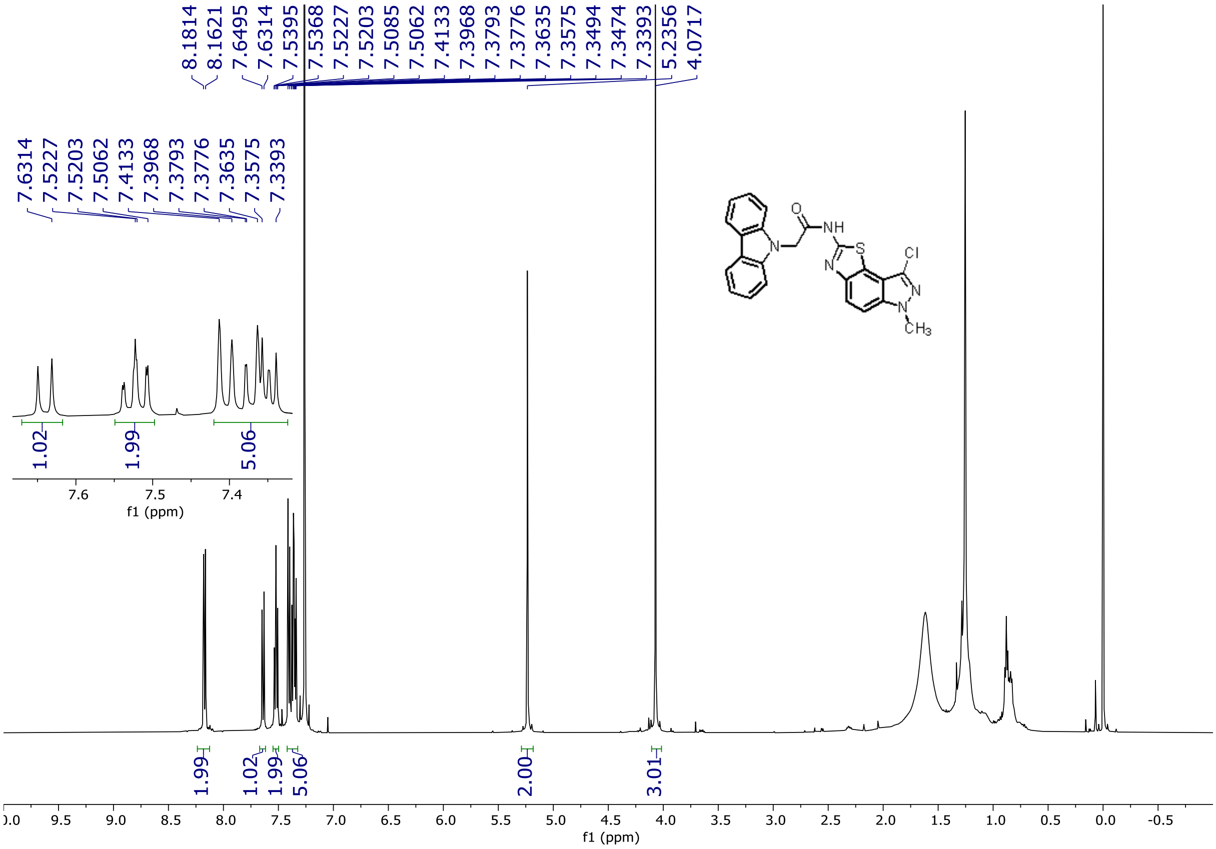

Figure S35. ^{1}H NMR spectrum of compound **TI58c** in $CDCl_3$.

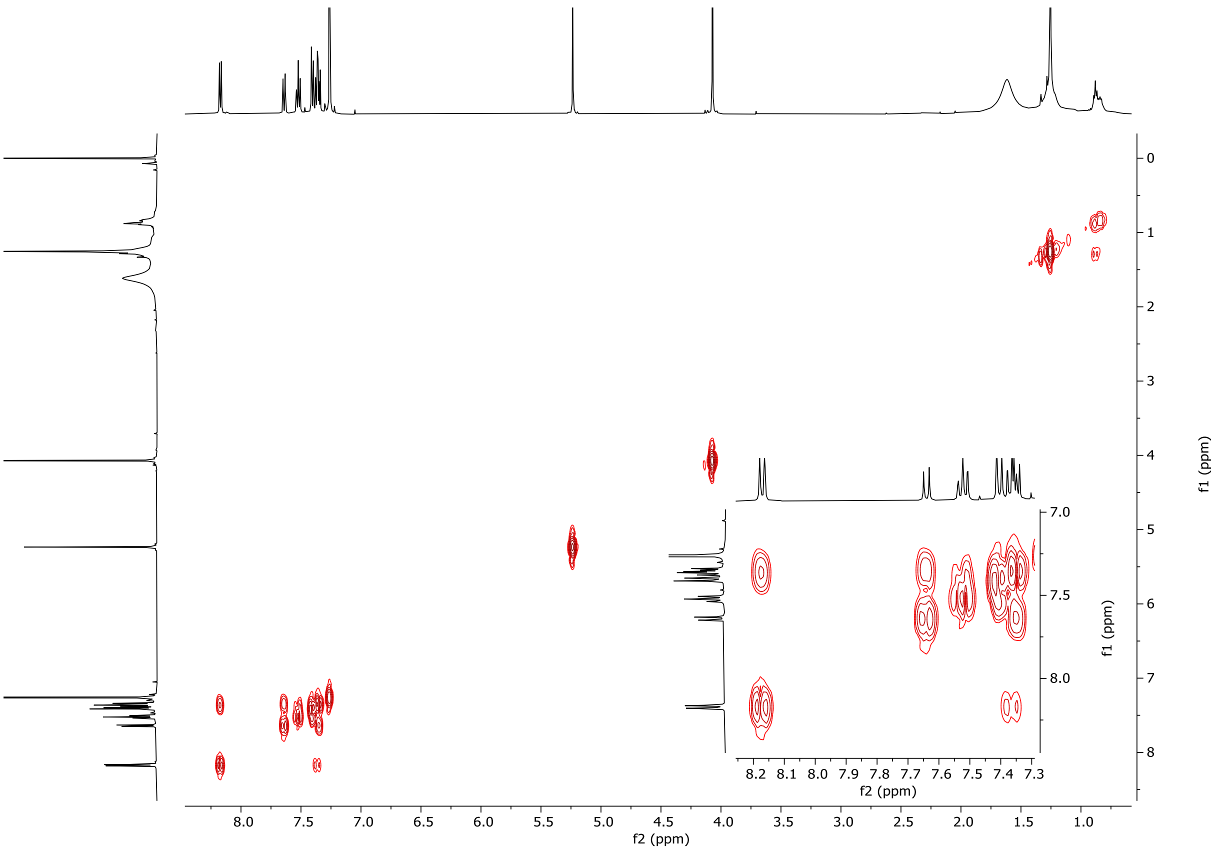

Figure 36. $^{1}H/^{1}H$ COSY spectrum of compound **TI58c** in $CDCl_3$.

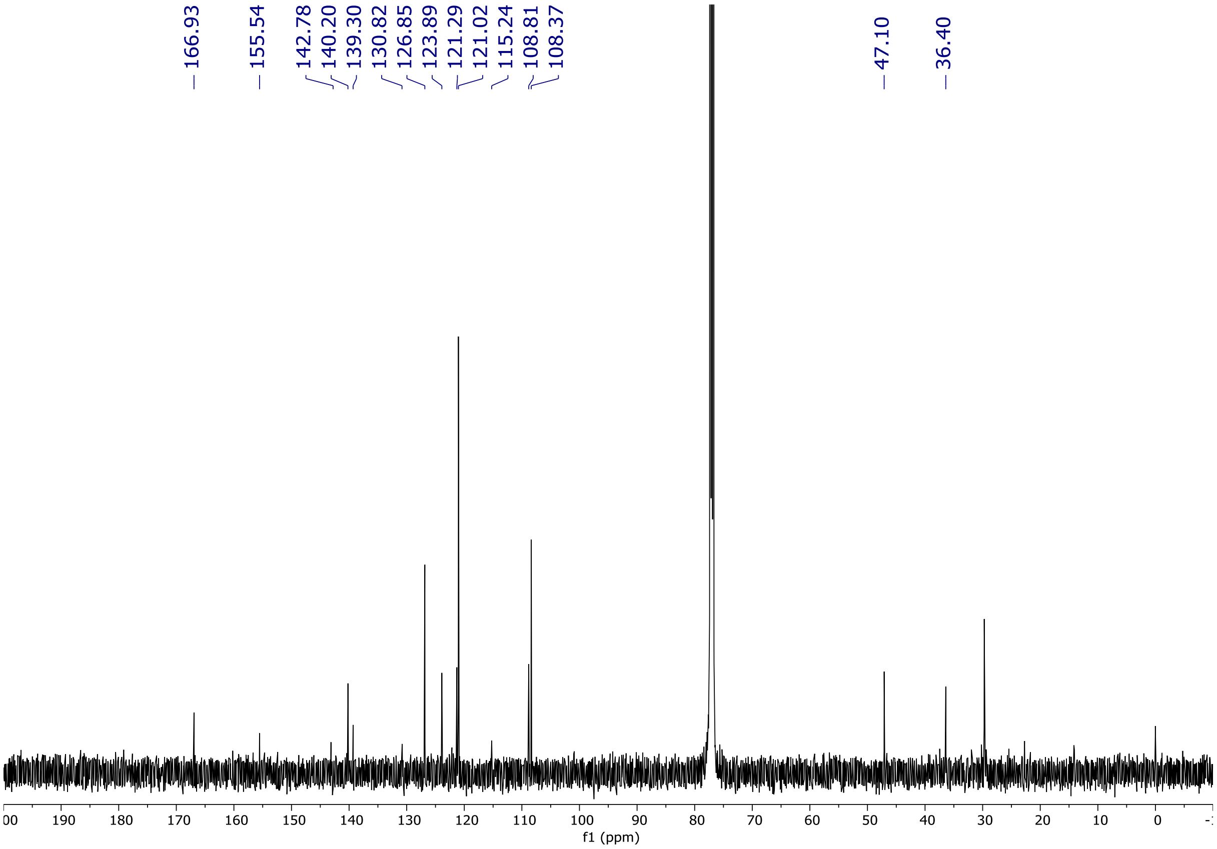

Figure S37. ^{13}C NMR spectrum of compound **TI58c** in $CDCl_3$.

2. MS-ESI(+) spectra

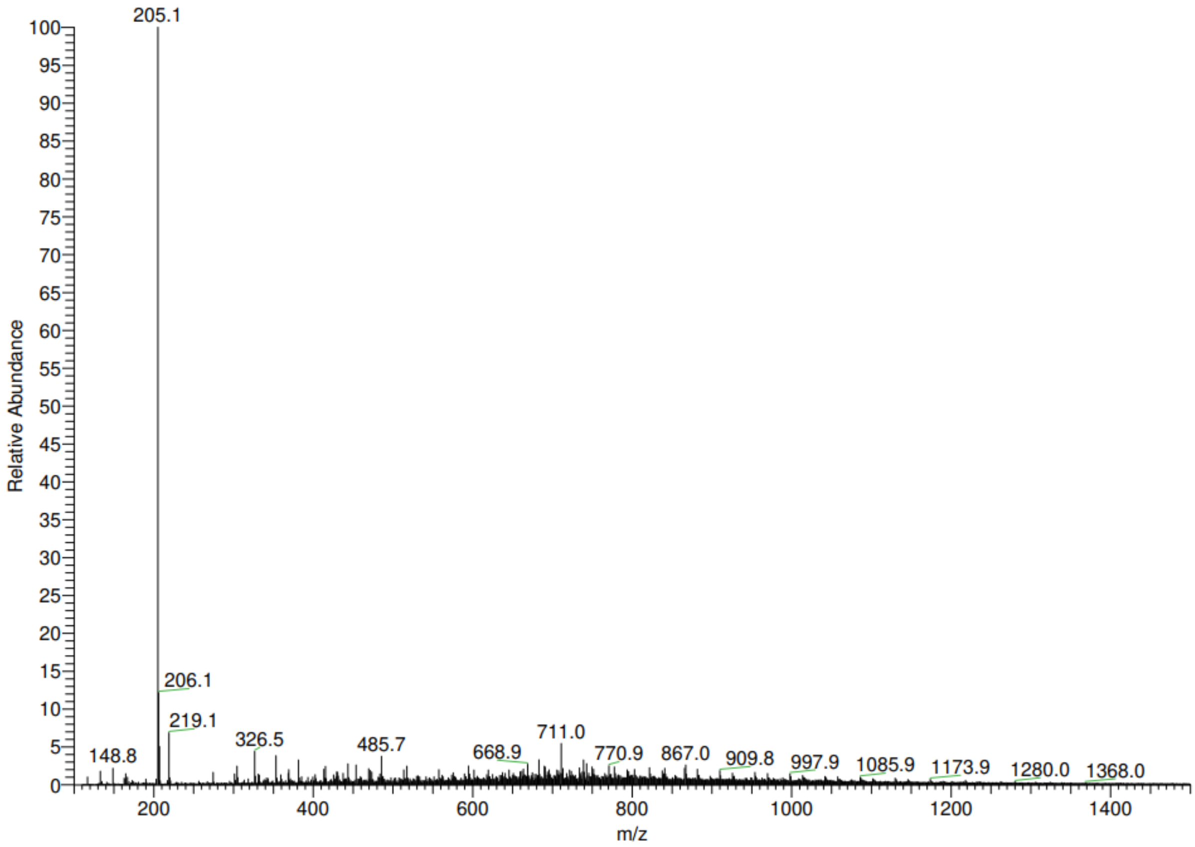

Figure S38. MS-ESI(+) spectrum of compound **5a**.

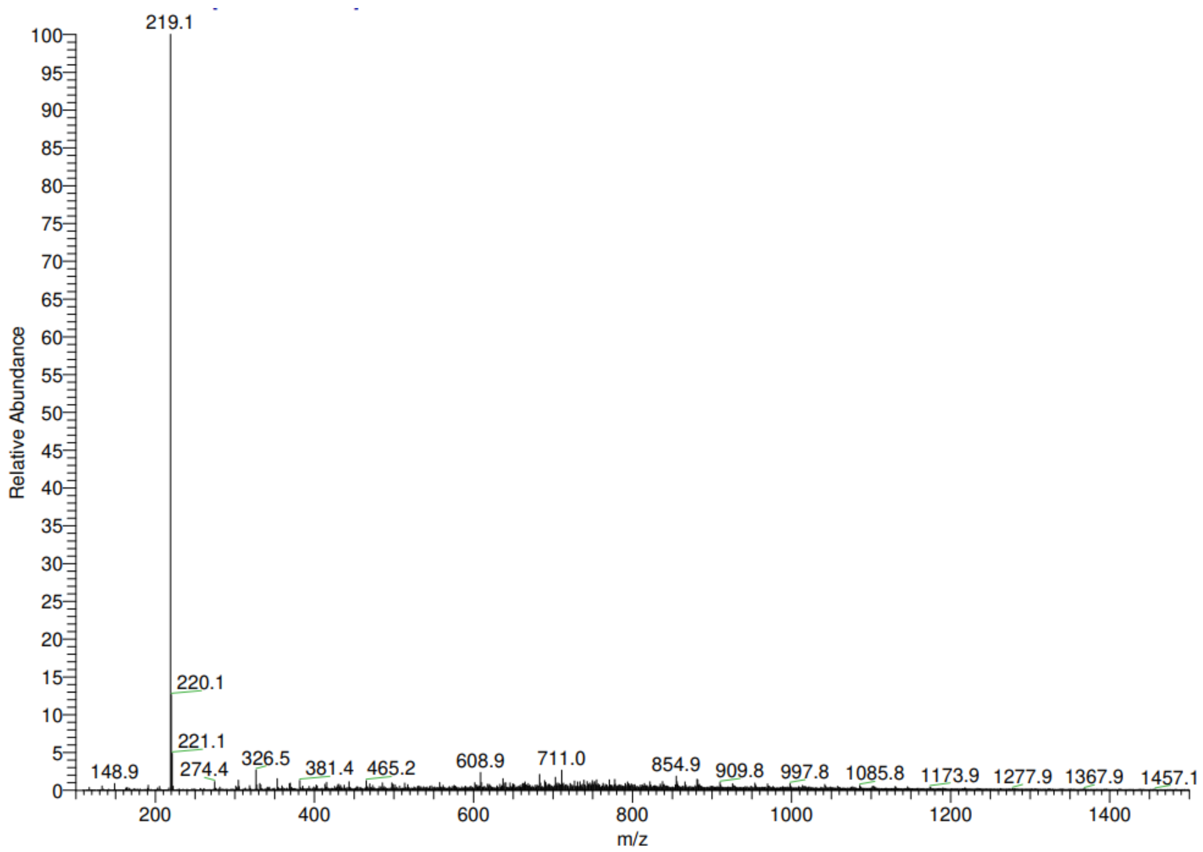

Figure S39. MS-ESI(+) spectrum of compound **5b**.

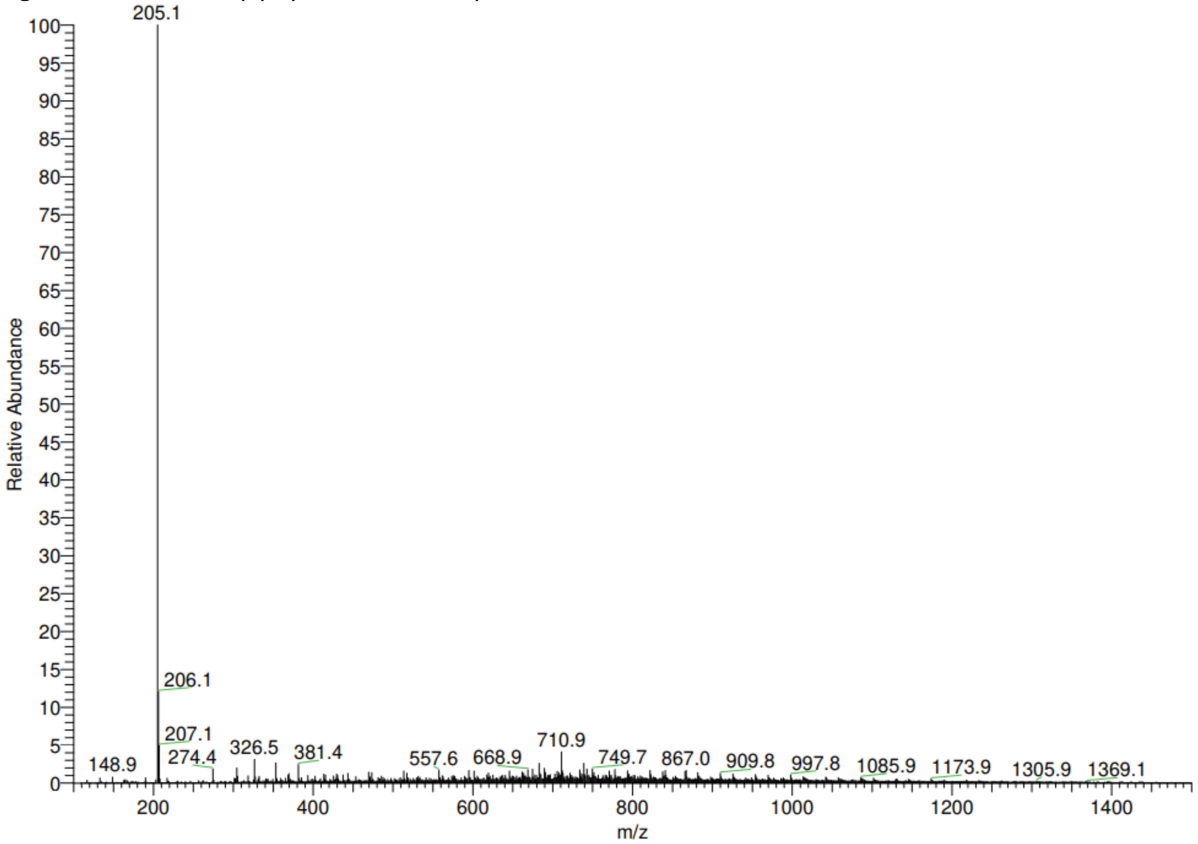

Figure S40. MS-ESI(+) spectrum of compound **5c**.

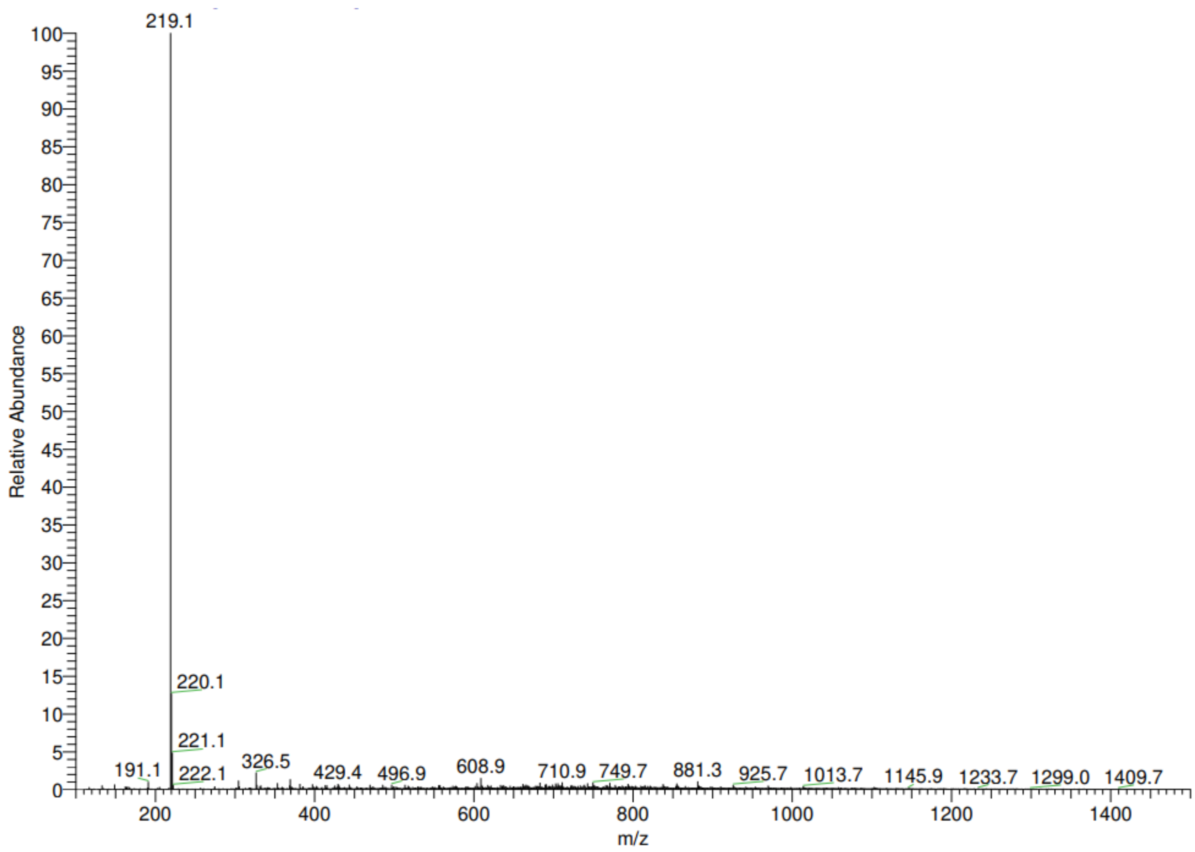

Figure S41. MS-ESI(+) spectrum of compound **5d**.

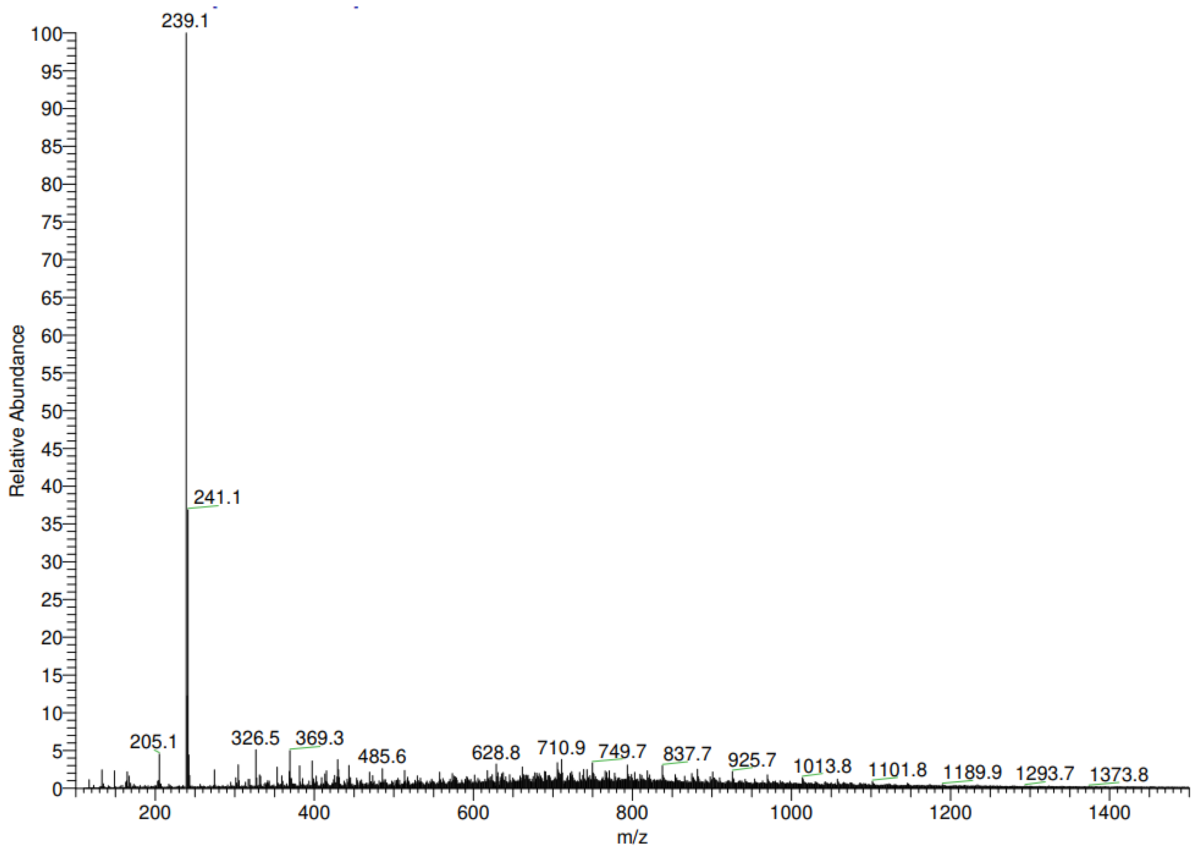

Figure S42. MS-ESI(+) spectrum of compound **5e**.

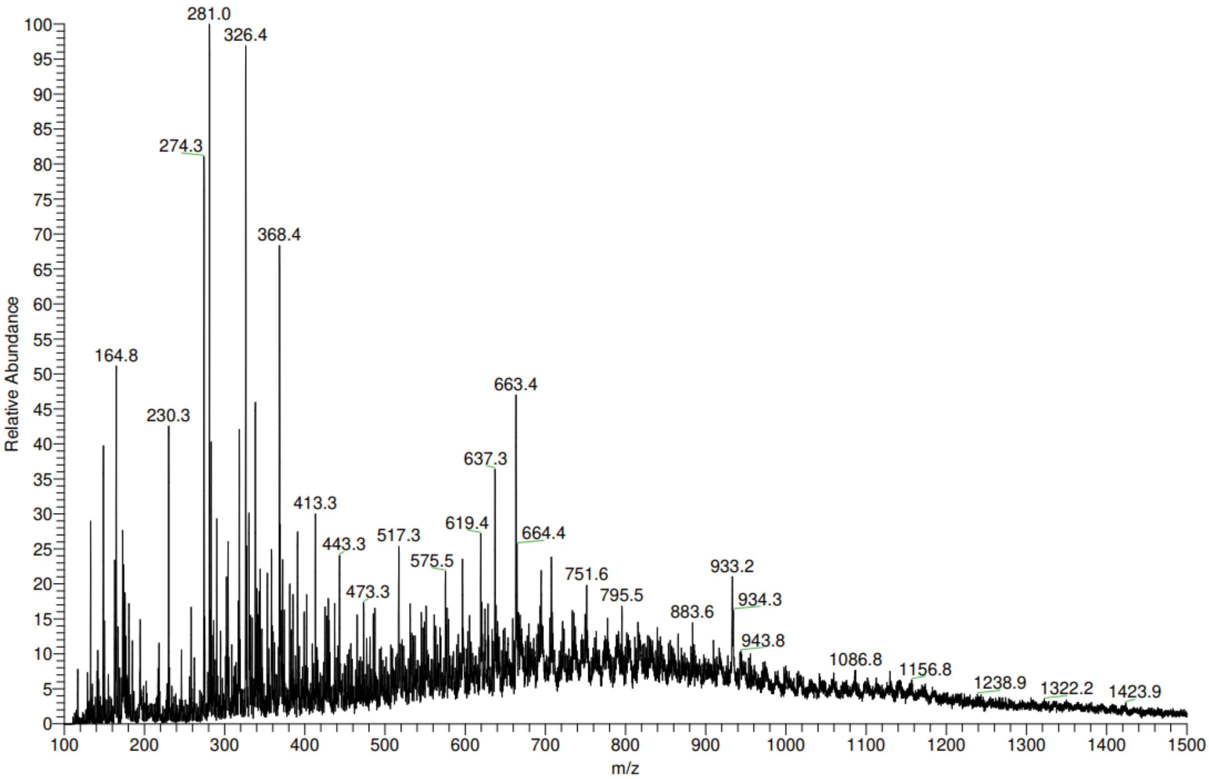

Figure S43. MS-ESI(+) spectrum of compound **6a**.

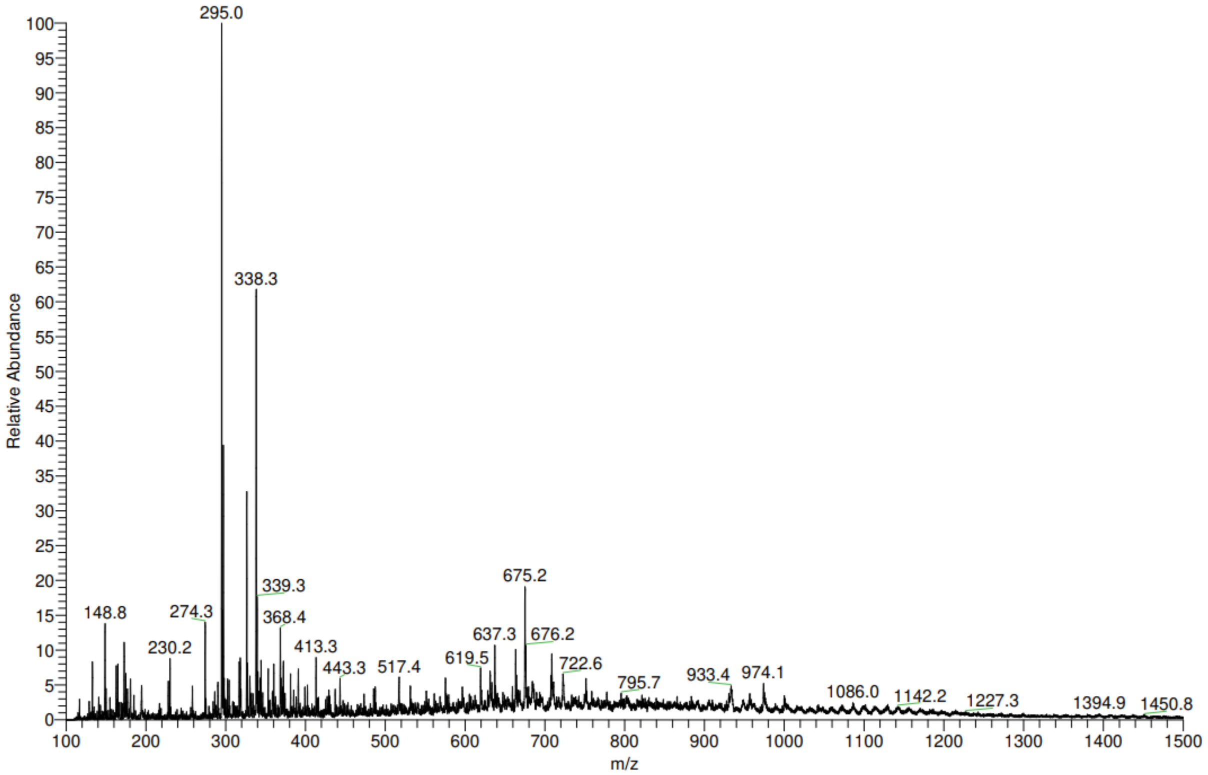

Figure S44. MS-ESI(+) spectrum of compound **6b**.

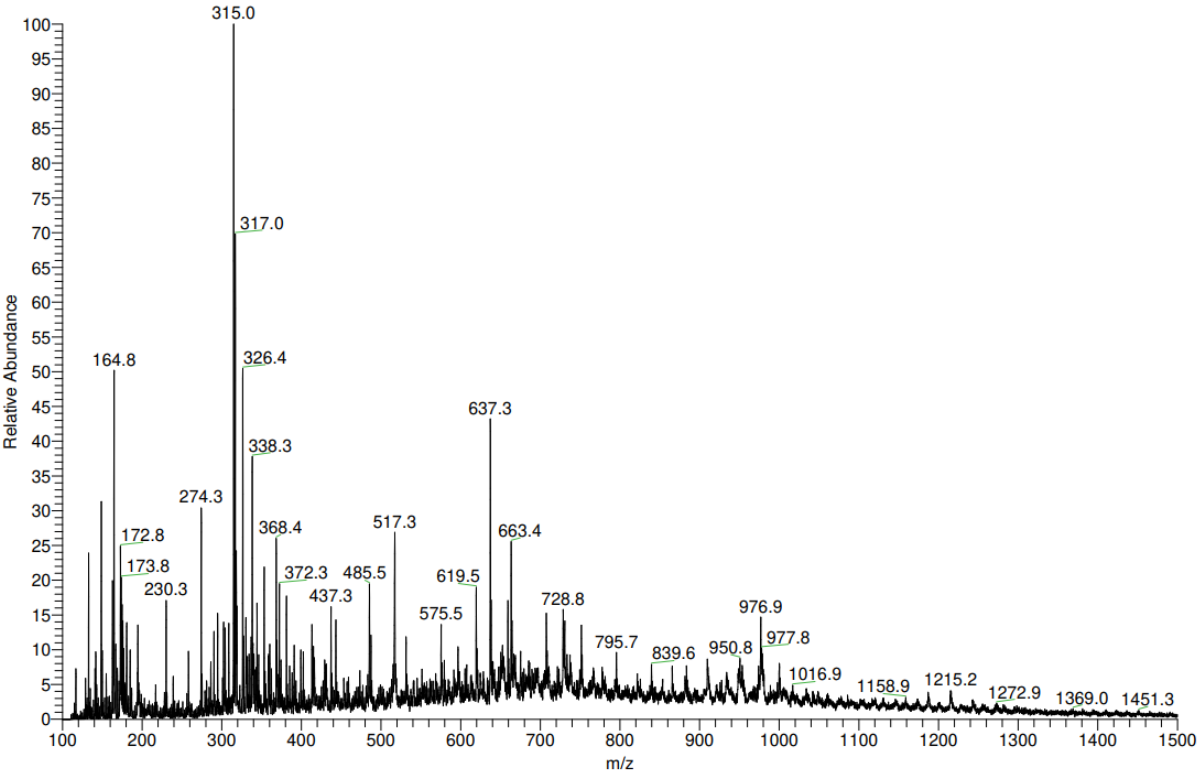

Figure S45. MS-ESI(+) spectrum of compound **6c**.

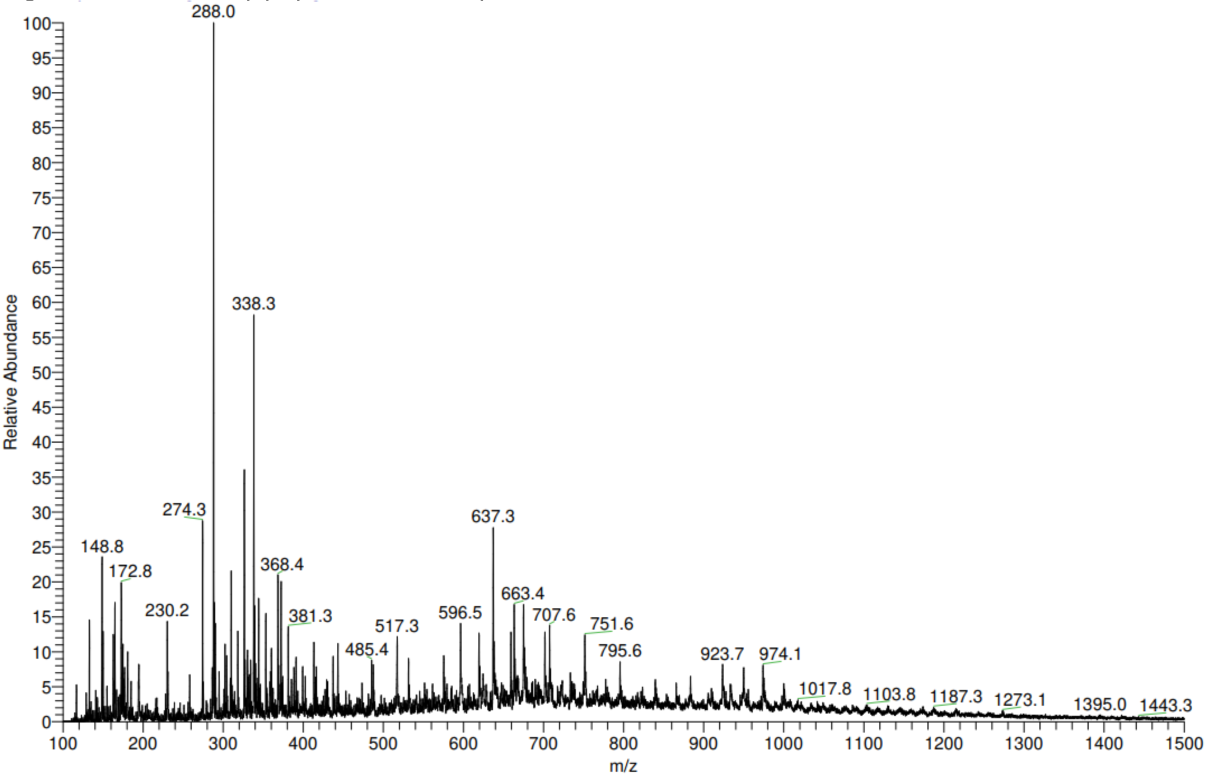

Figure S46. MS-ESI(+) spectrum of compound **7a**.

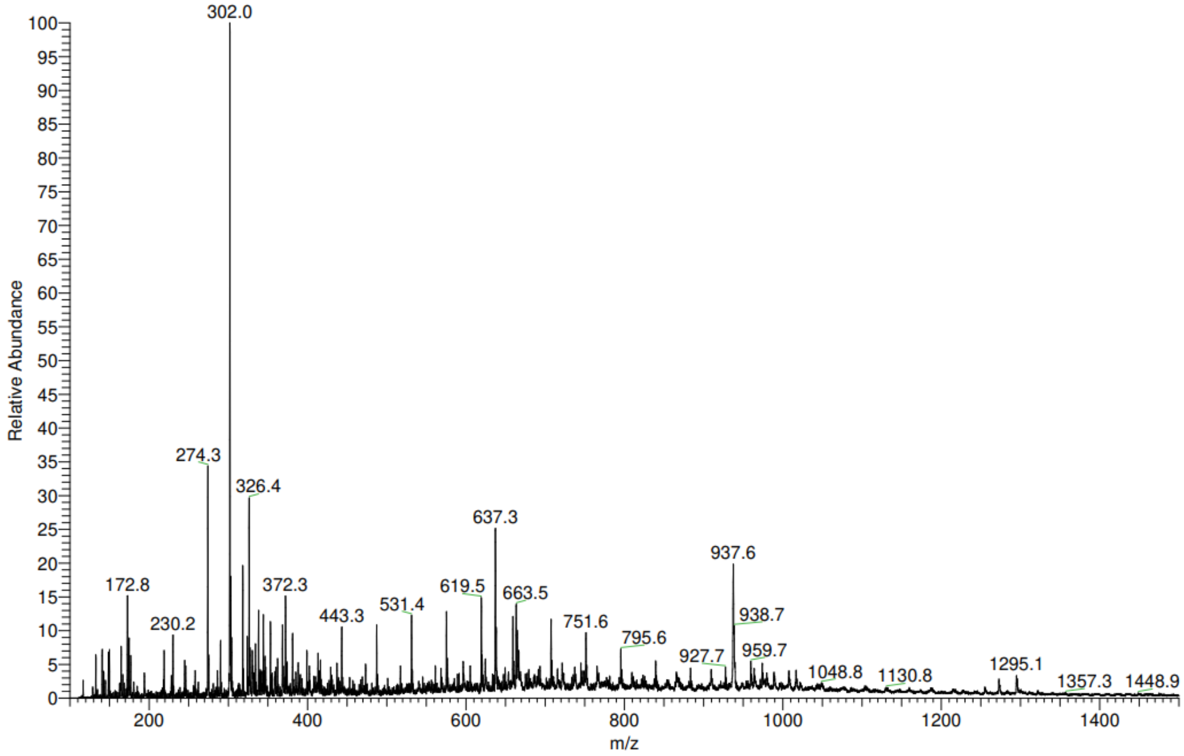

Figure S47. MS-ESI(+) spectrum of compound **7b**.

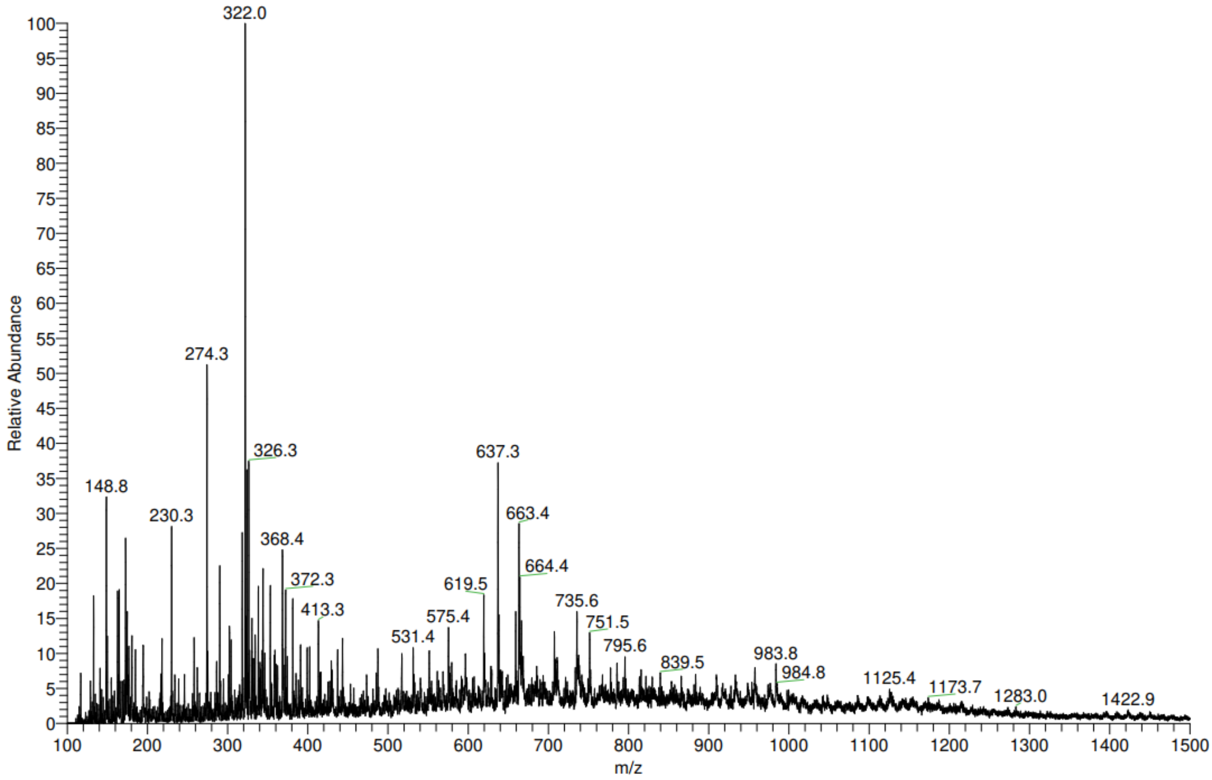

Figure S48. MS-ESI(+) spectrum of compound **7c**.

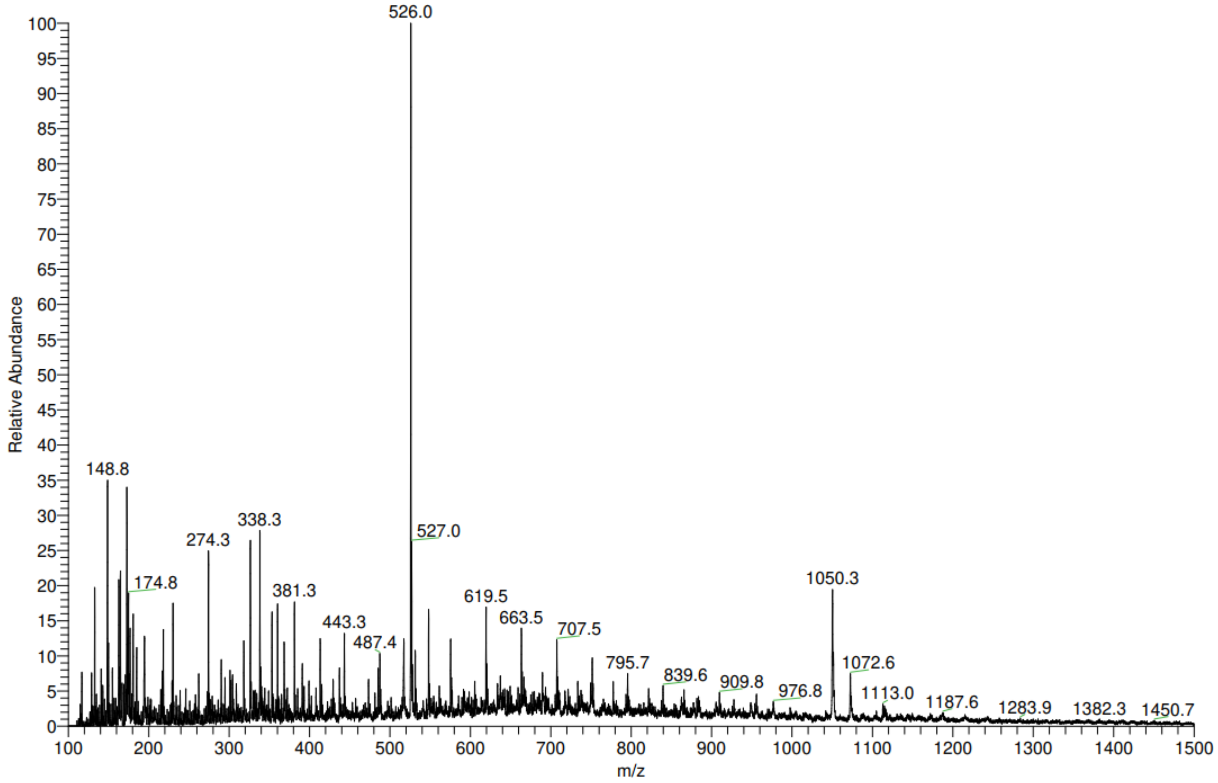

Figure S49. MS-ESI(+) spectrum of compound **TI45a.**

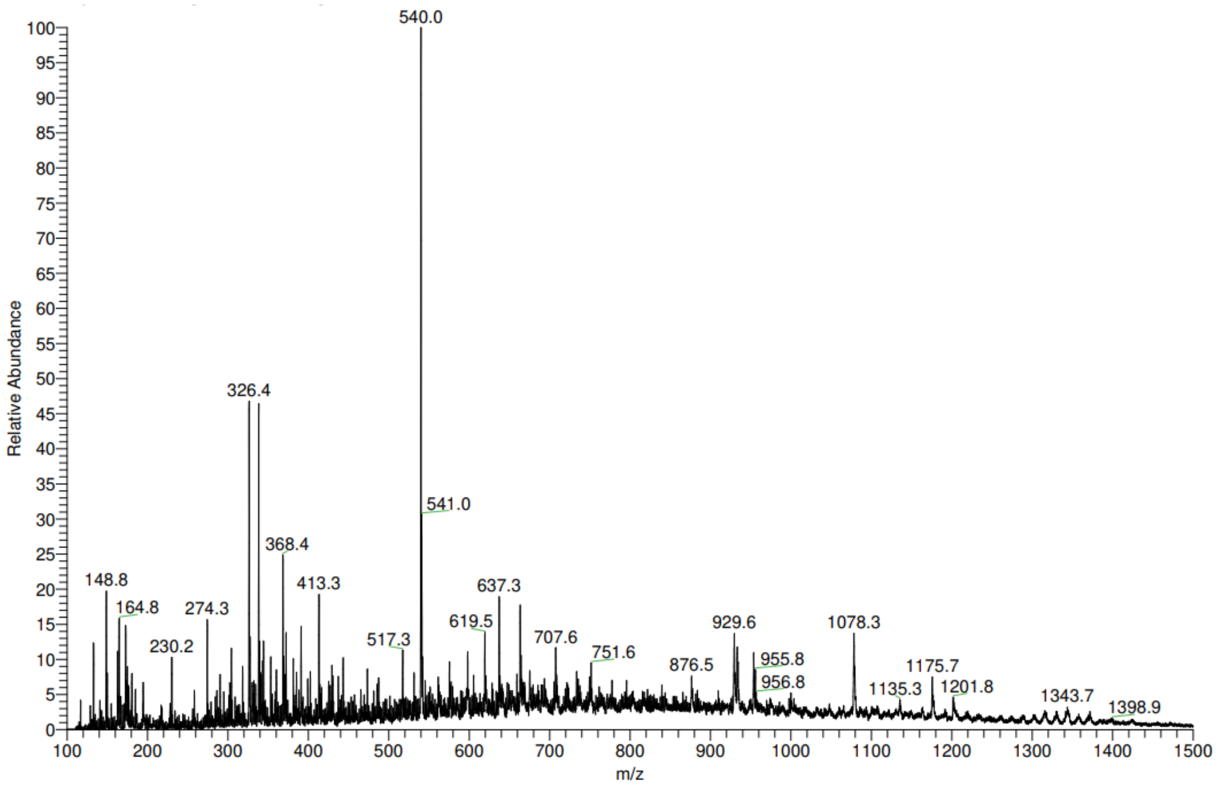

Figure S50. MS-ESI(+) spectrum of compound **TI45b**.

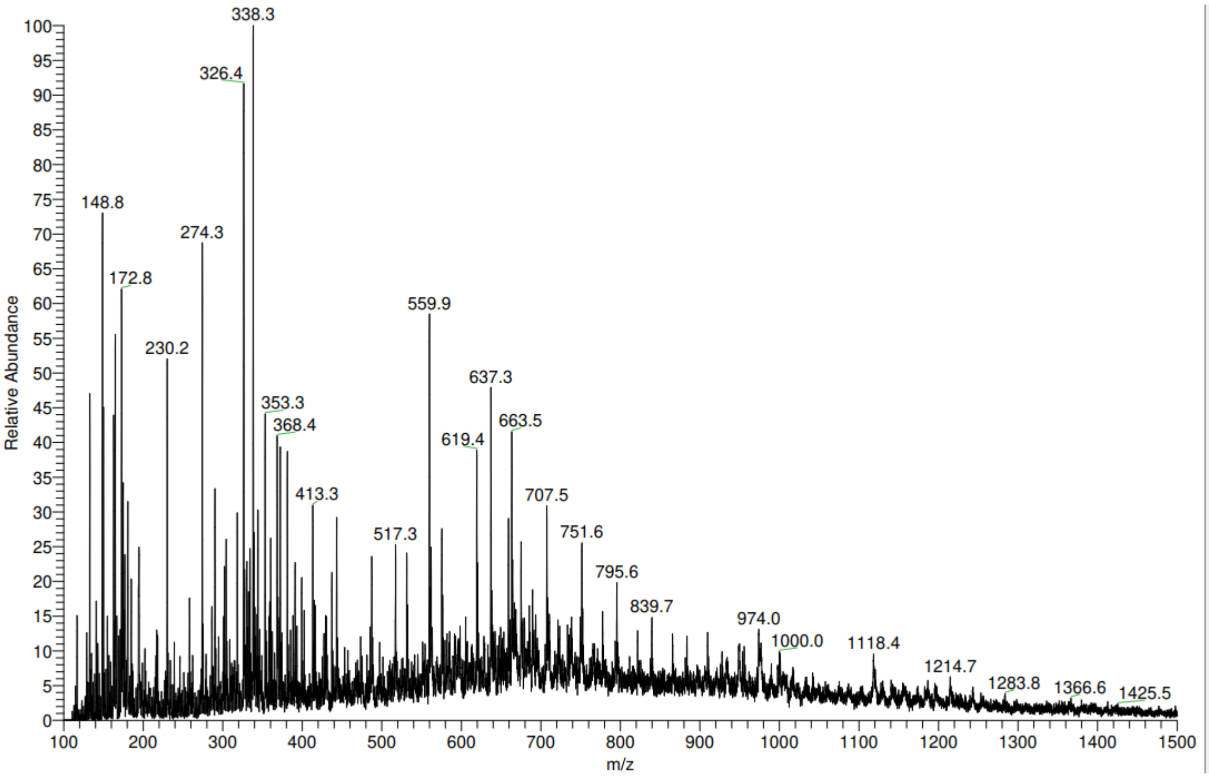

Figure S51. MS-ESI(+) spectrum of compound **TI45c**.

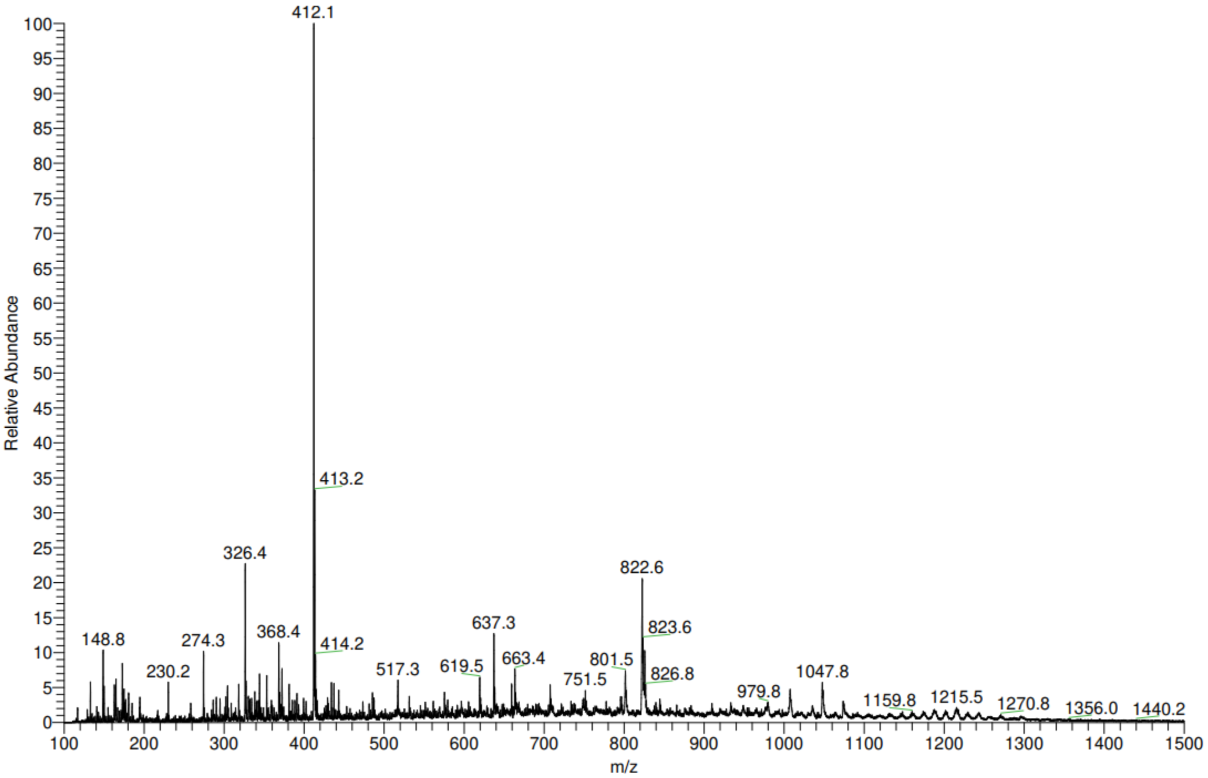

Figure S52. MS-ESI(+) spectrum of compound **TI58a**.

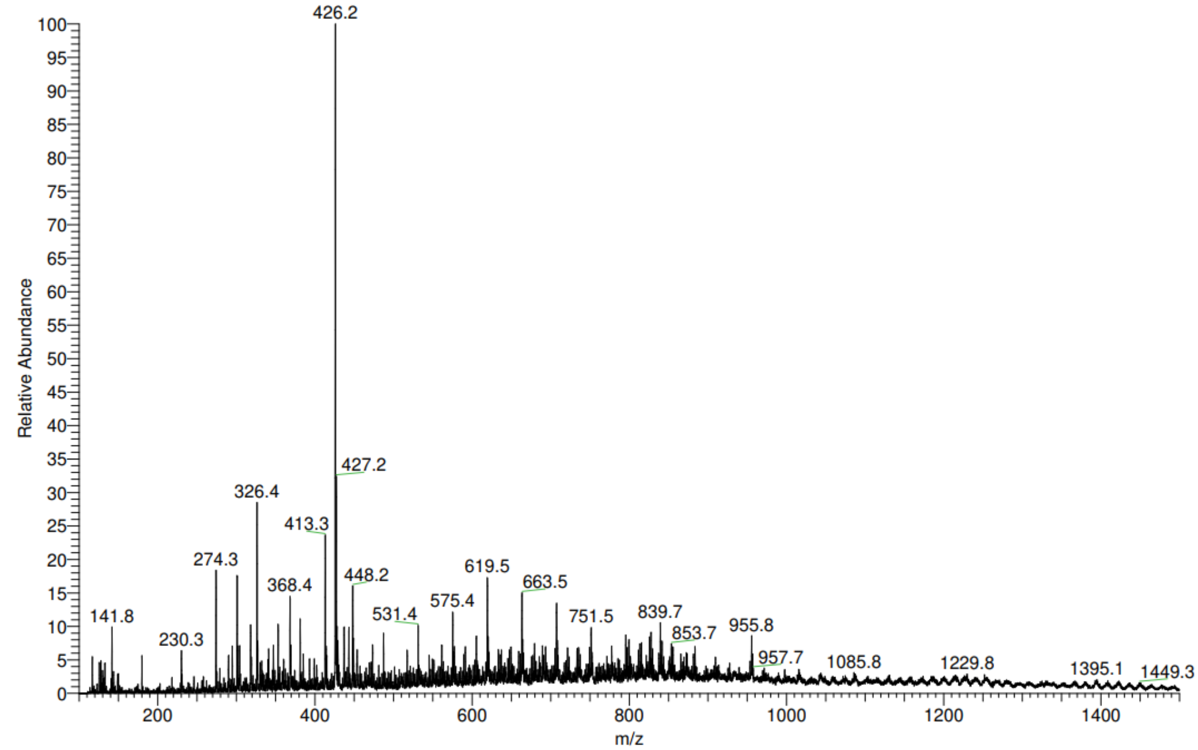

Figure S53. MS-ESI(+) spectrum of compound **TI58b**.

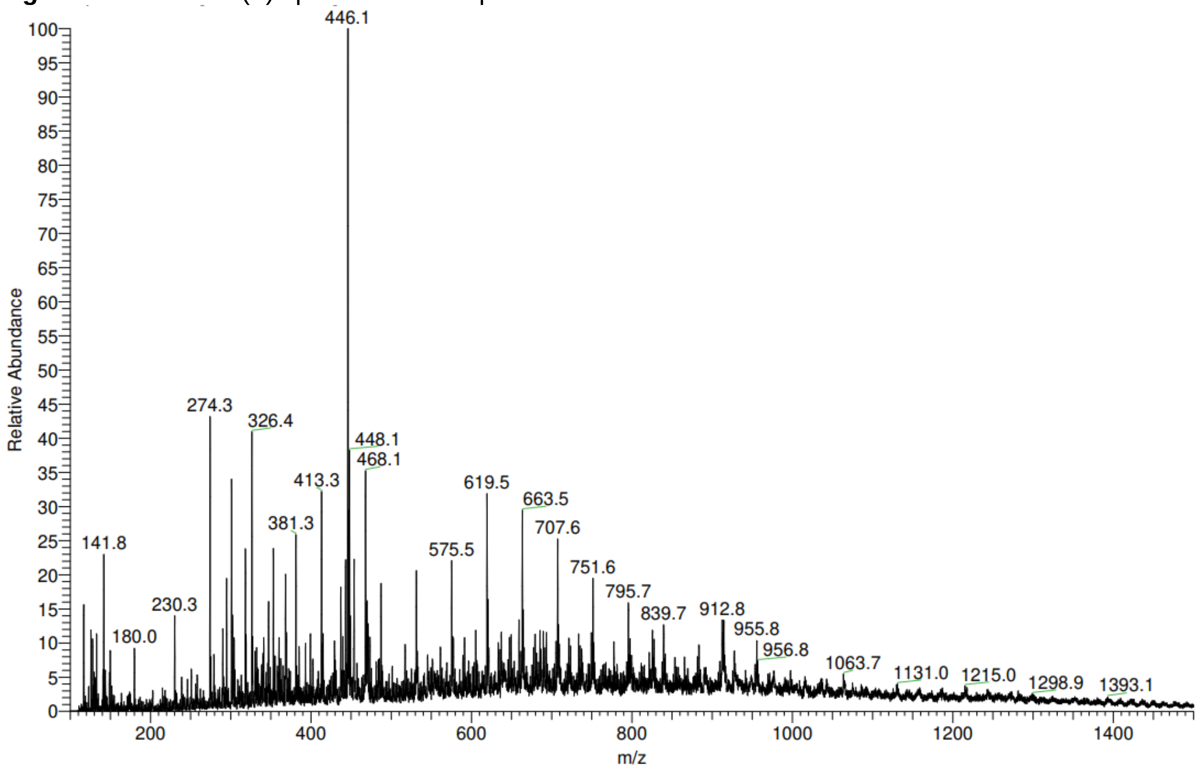

Figure S54. MS-ESI(+) spectrum of compound **TI58c**.

3. HRMS-ESI(+) spectra

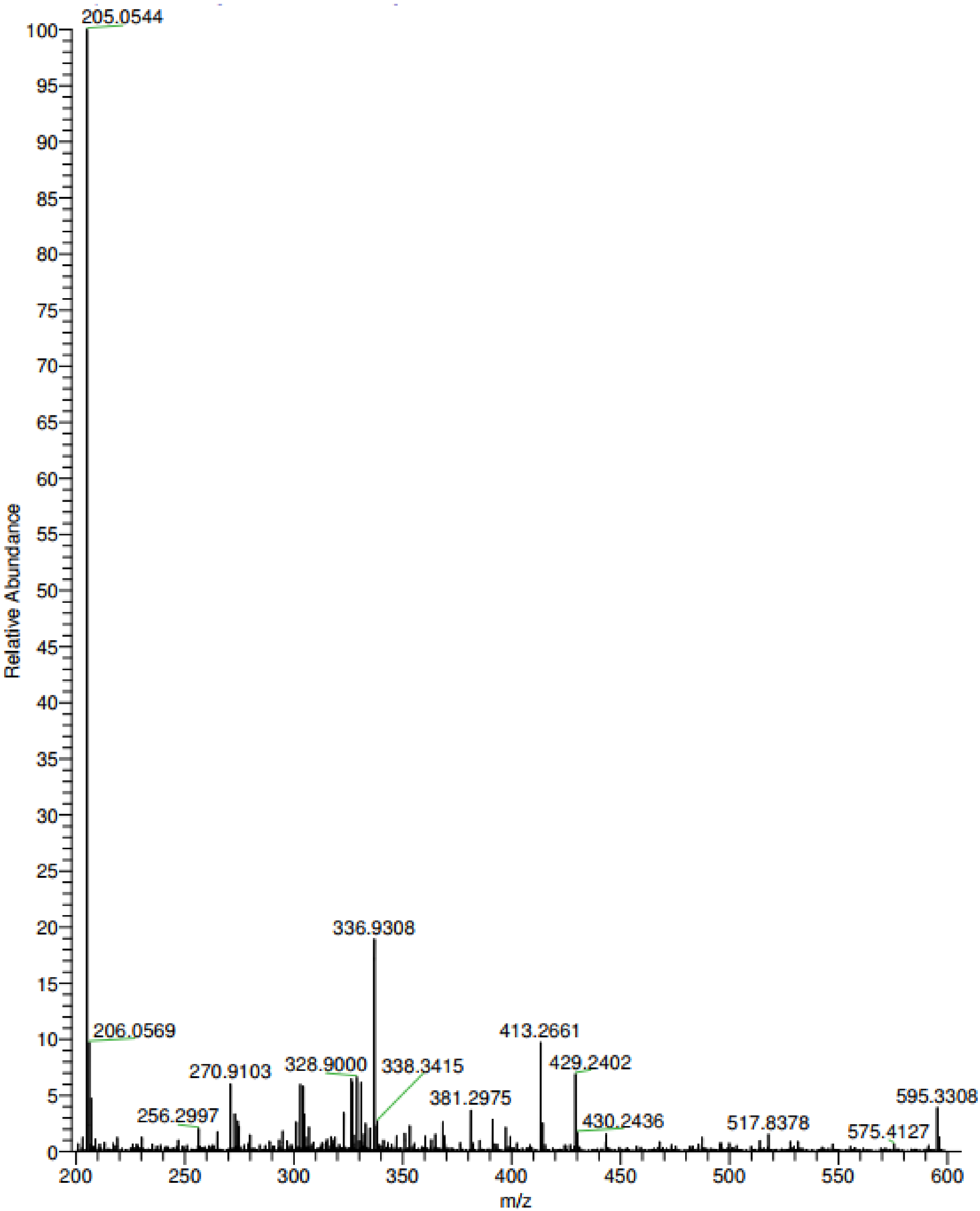

Figure S55. HRMS-ESI(+) spectrum of compound **5a**.

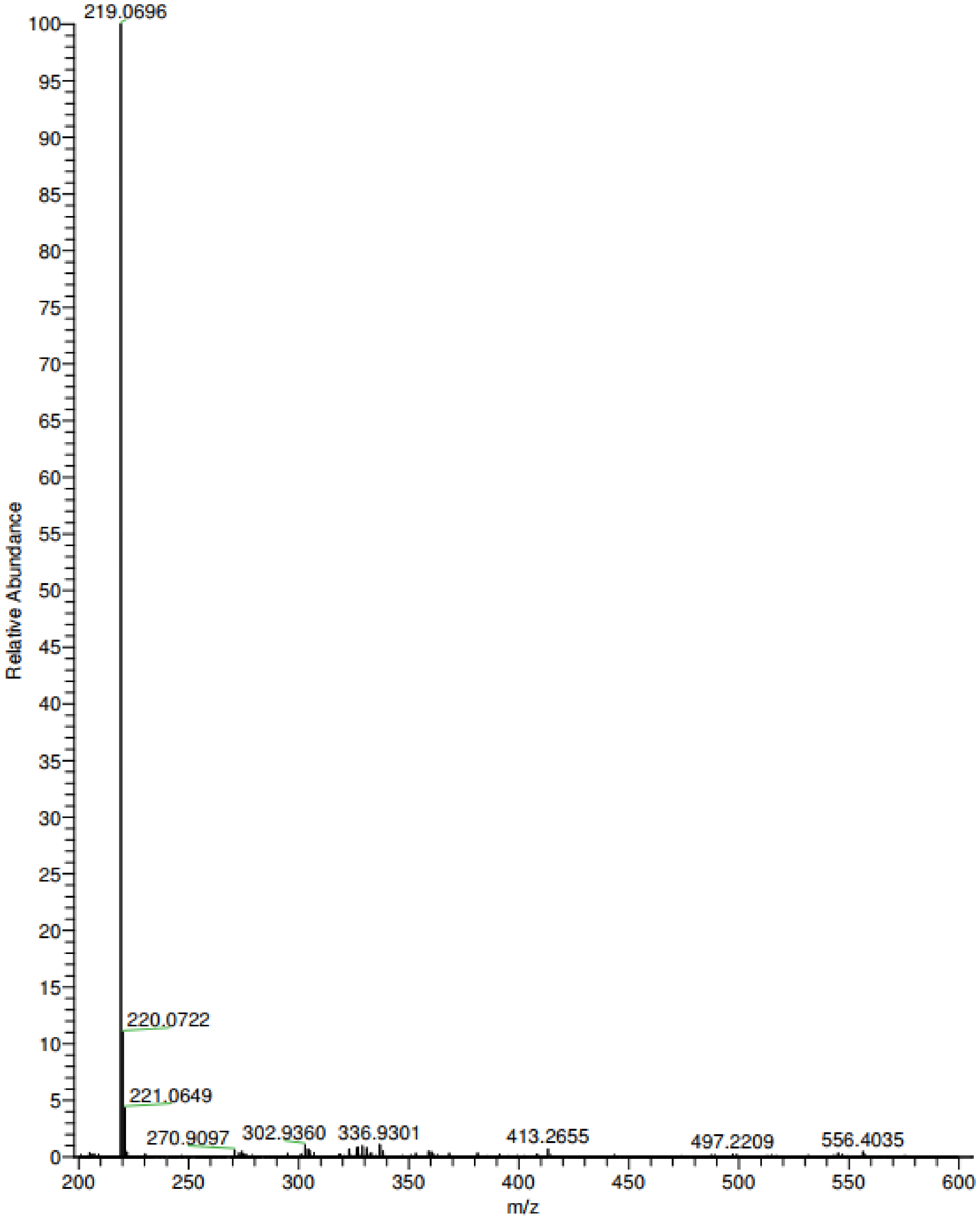

Figure S56. HRMS-ESI(+) spectrum of compound **5b**.

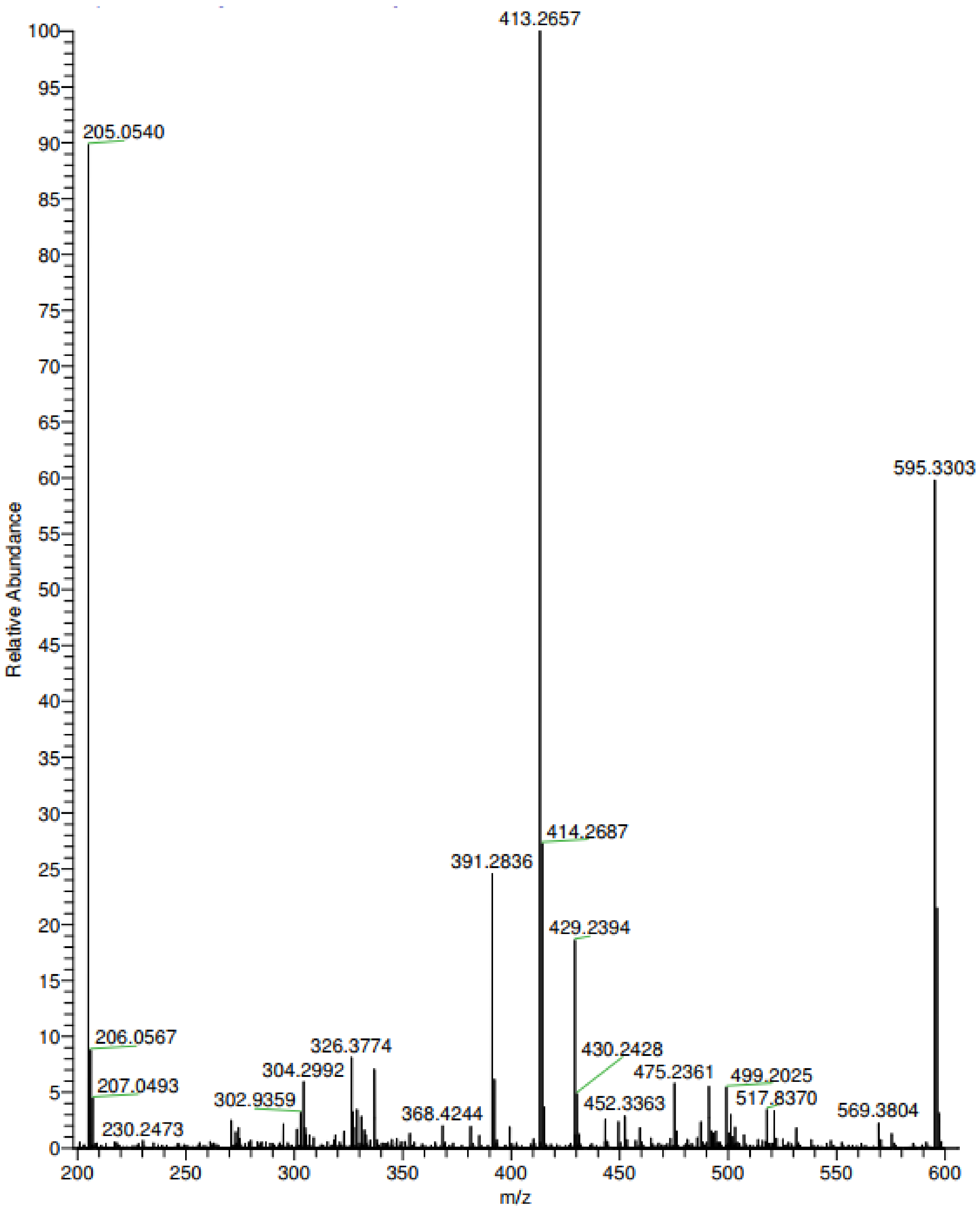

Figure S57. HRMS-ESI(+) spectrum of compound **5c**.

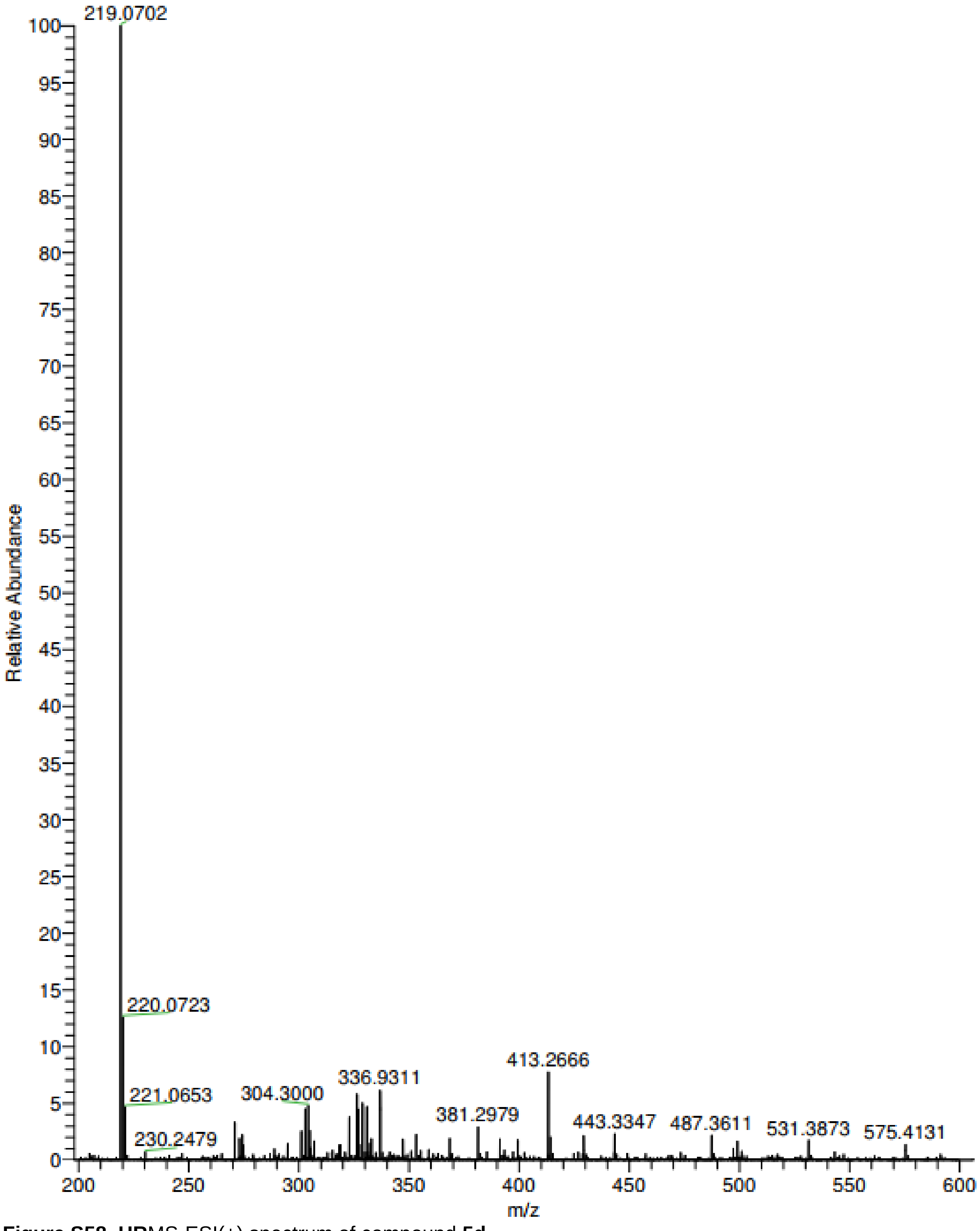

Figure S58. HRMS-ESI(+) spectrum of compound **5d**.

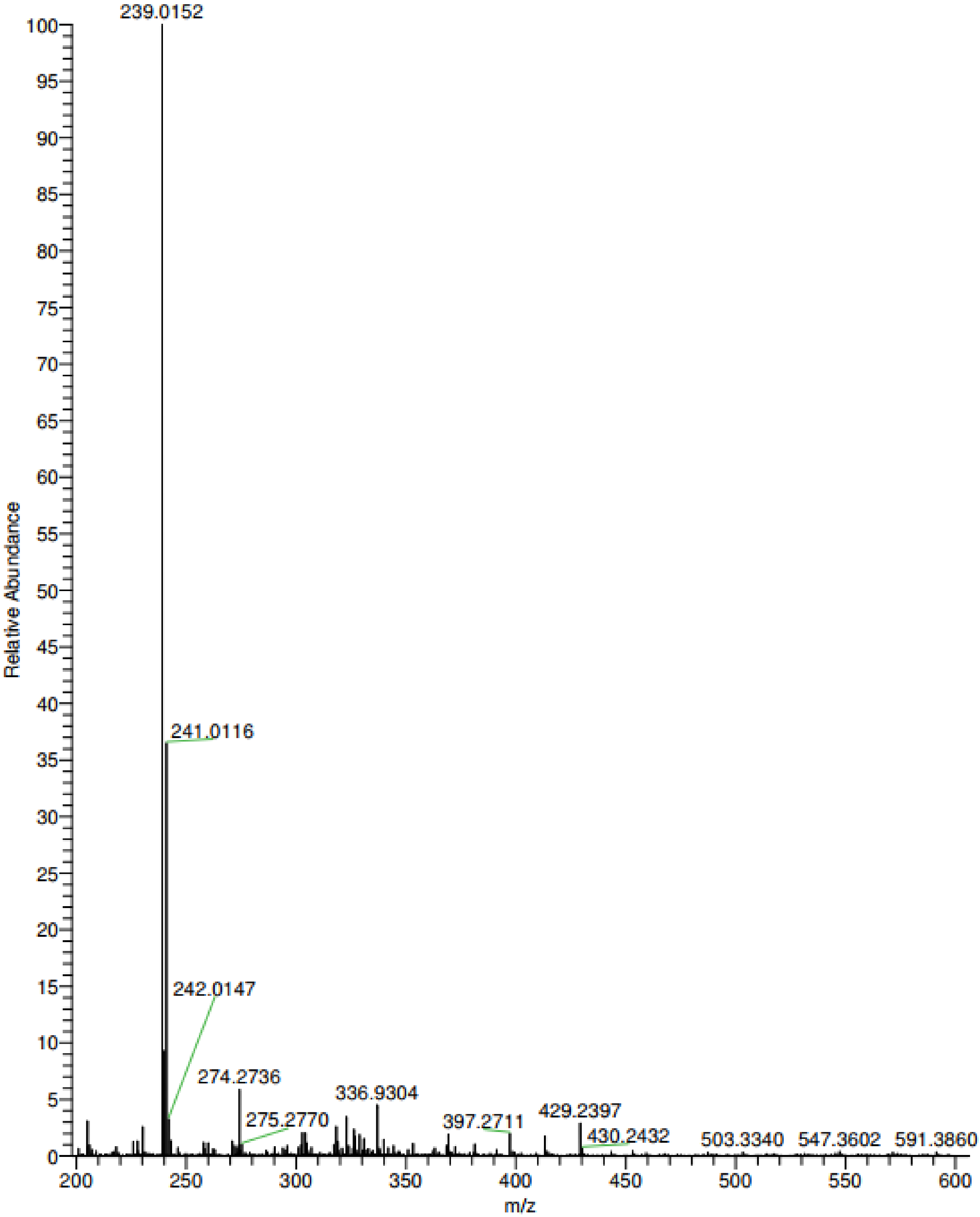

Figure S59. HRMS-ESI(+) spectrum of compound **5e**.

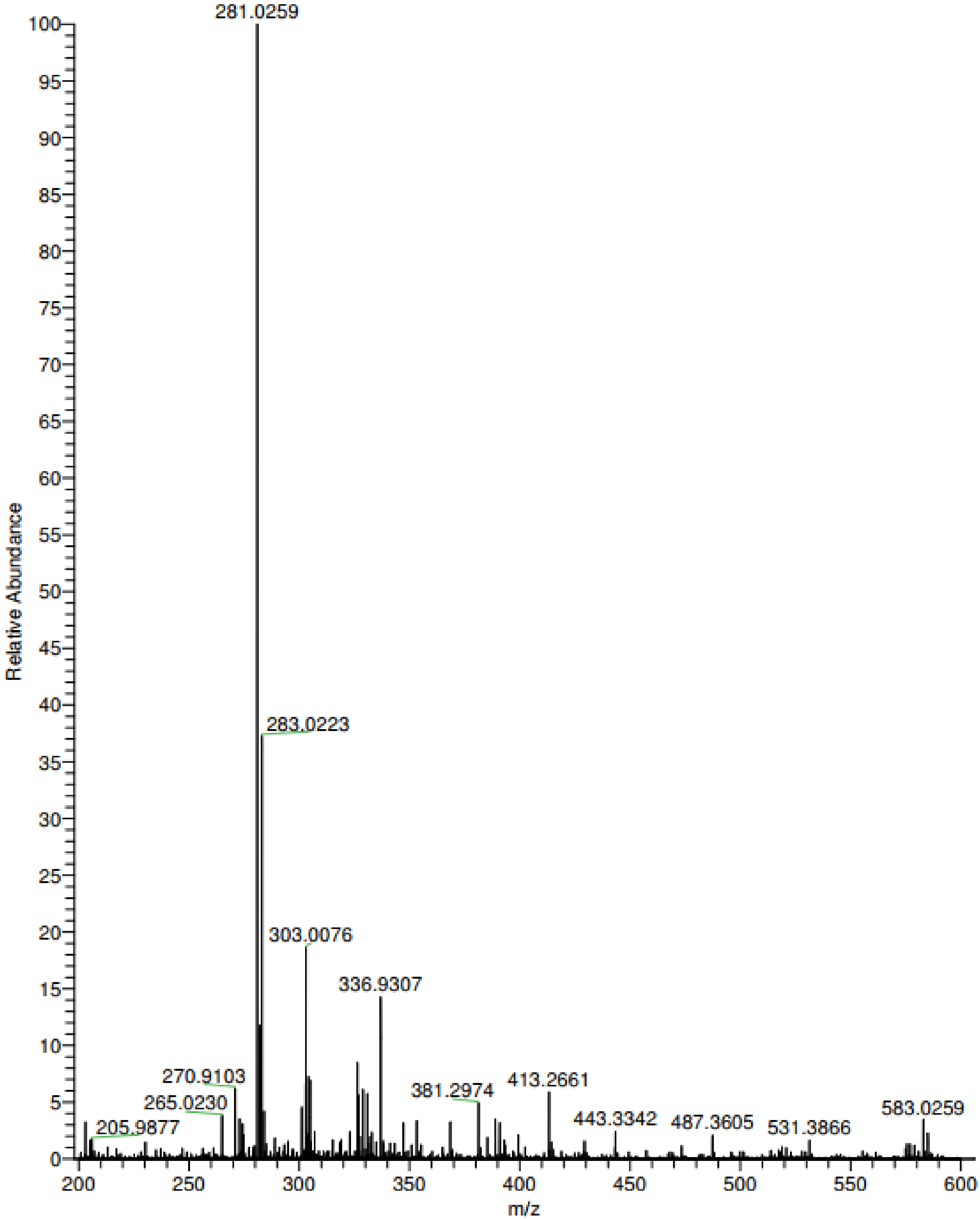

Figure S60. HRMS-ESI(+) spectrum of compound **6a**.

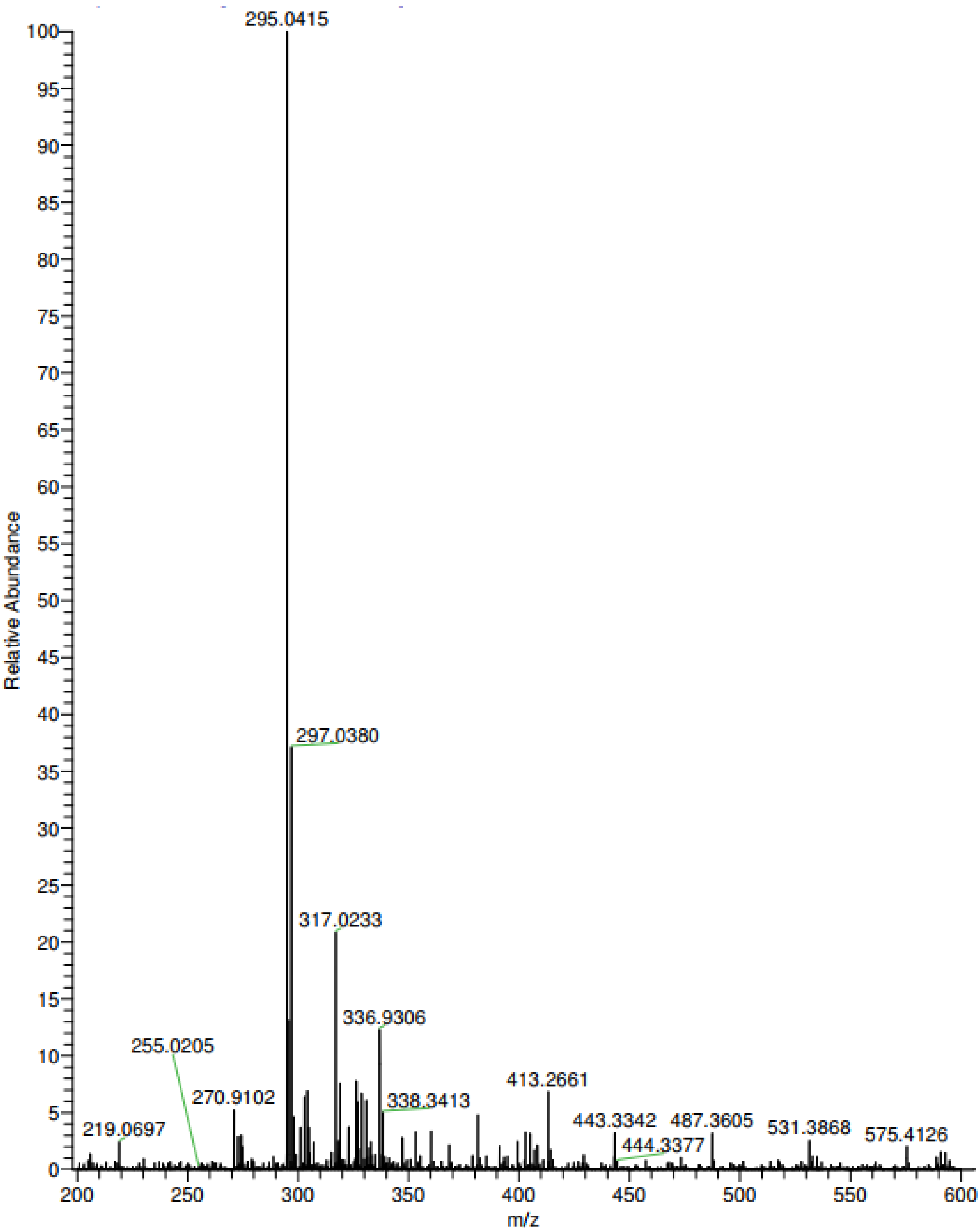

Figure S61. HRMS-ESI(+) spectrum of compound **6b**.

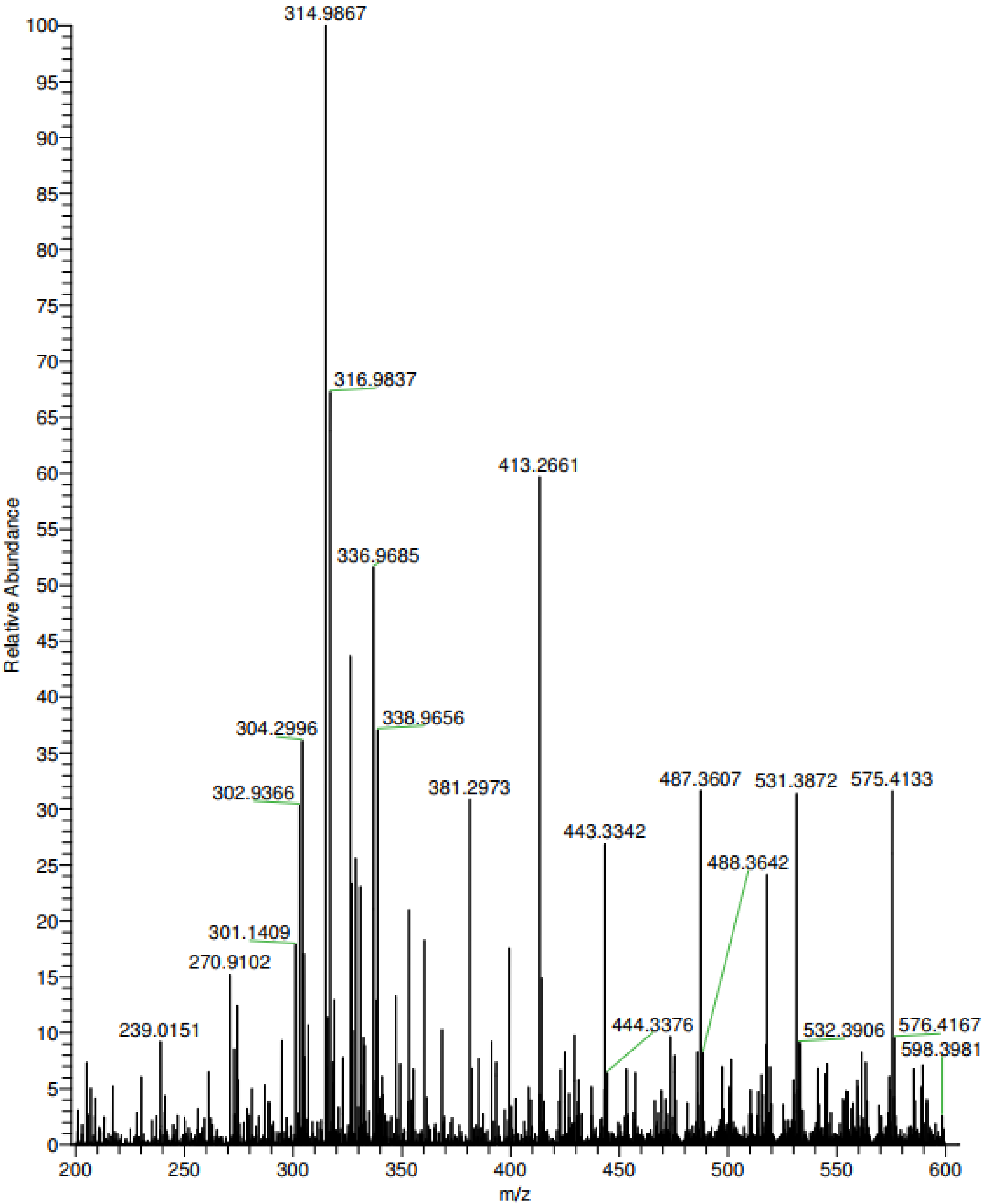

Figure S62. HRMS-ESI(+) spectrum of compound **6c**.

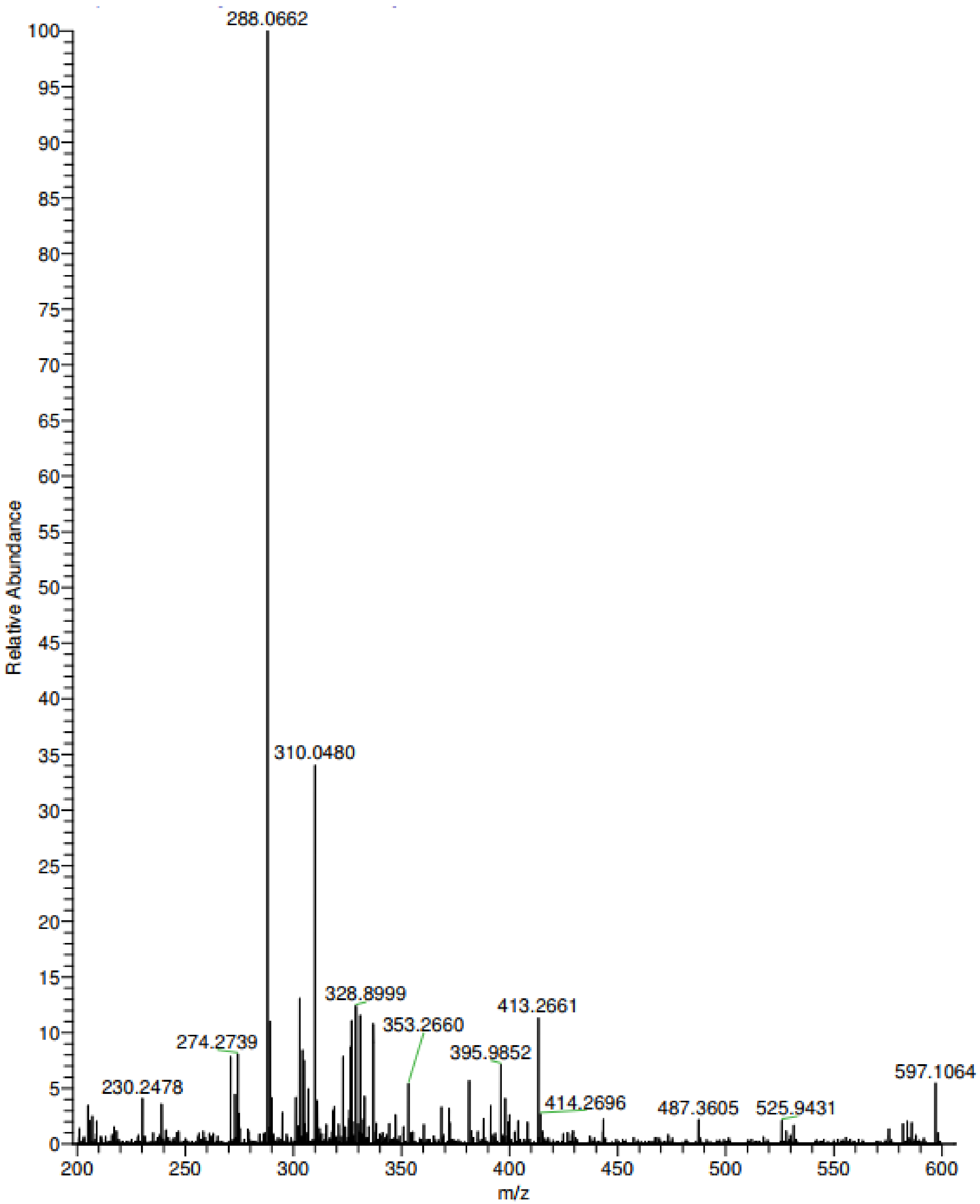

Figure S63. HRMS-ESI(+) of compound **7a**.

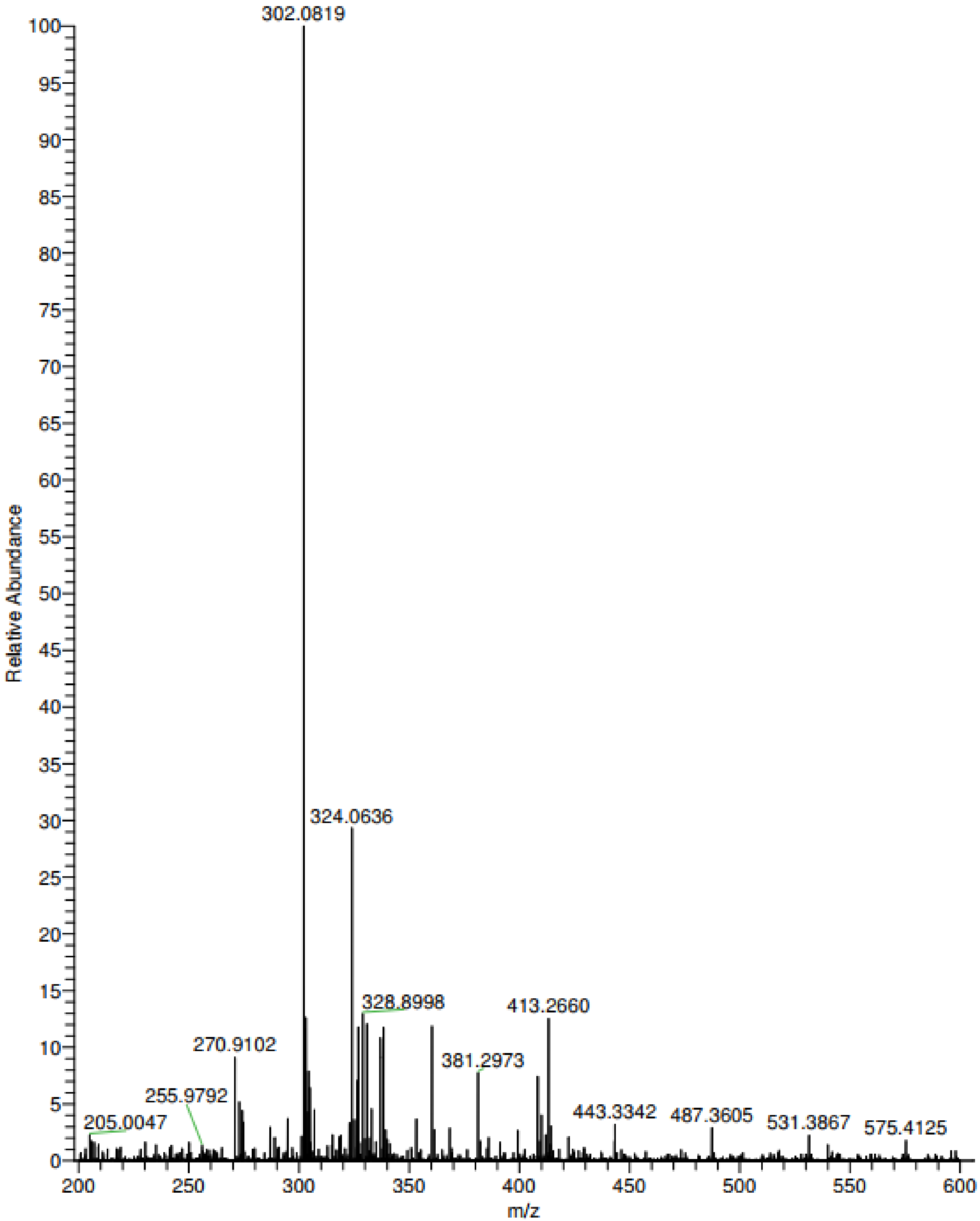

Figure S64. HRMS-ESI(+) of compound **7b**.

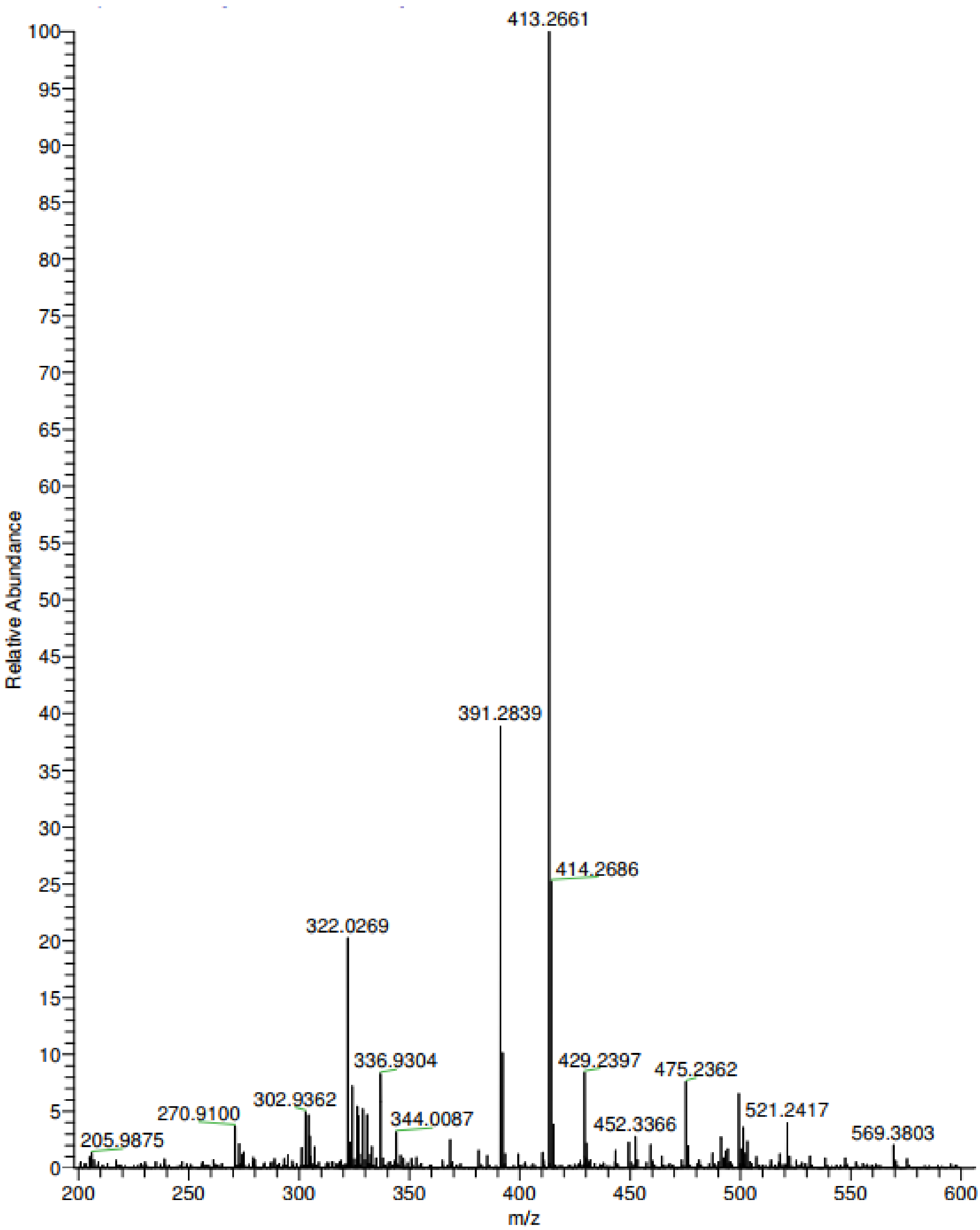

Figure S65. HRMS-ESI(+) of compound **7c**.

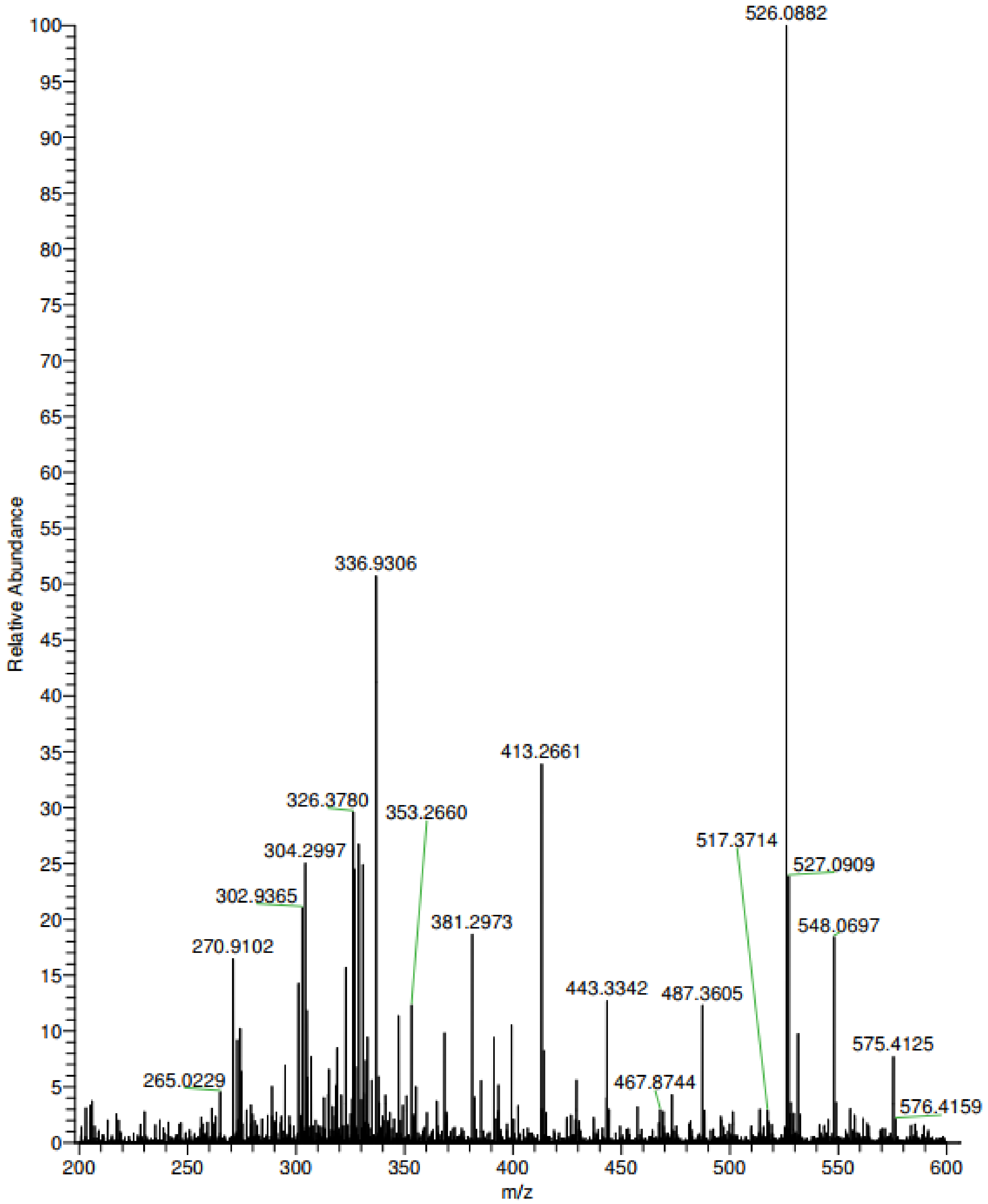

Figure S66. HRMS-ESI(+) of compound **Tl45a**.

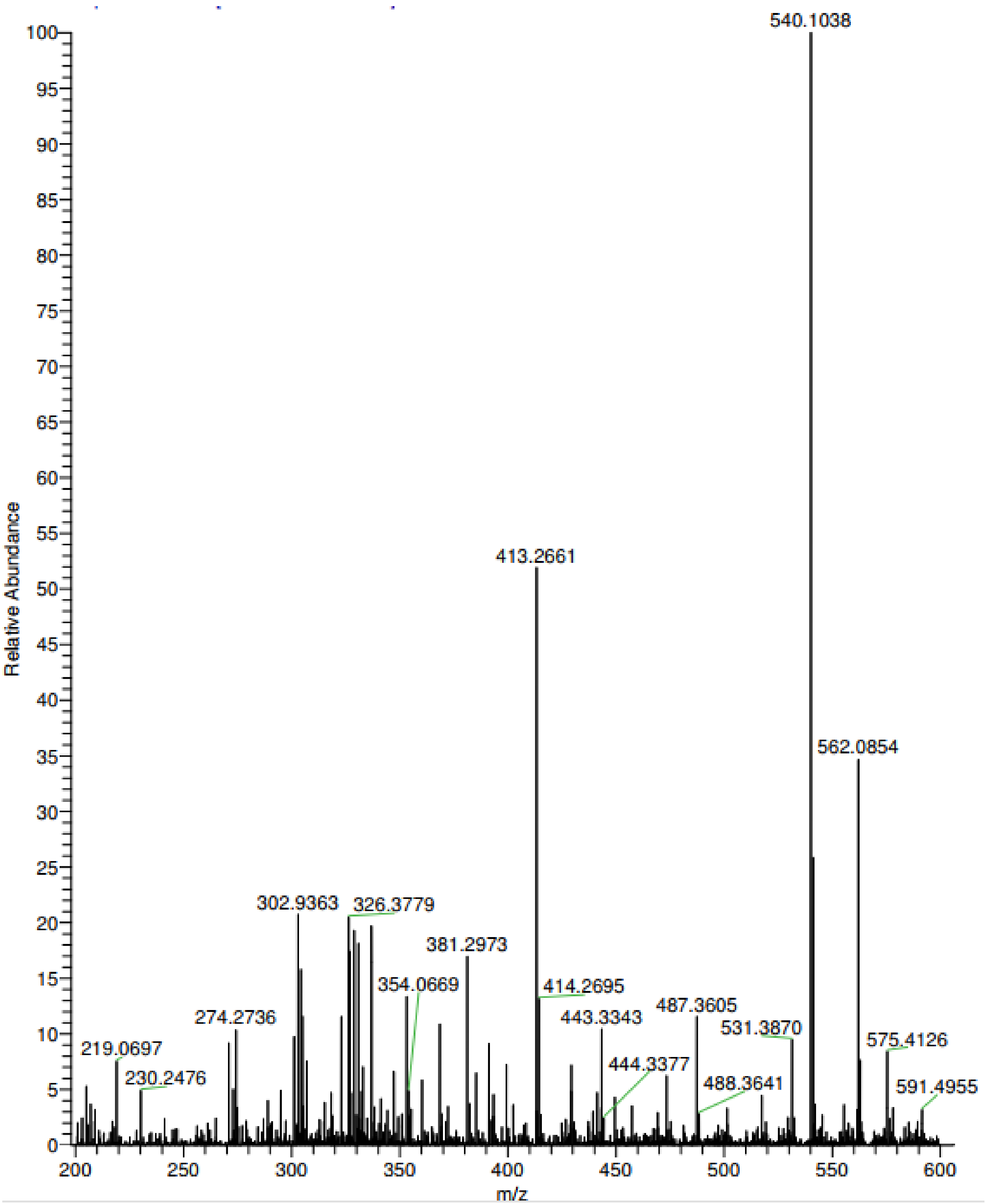

Figure S67. HRMS-ESI(+) of compound **Tl45b**.

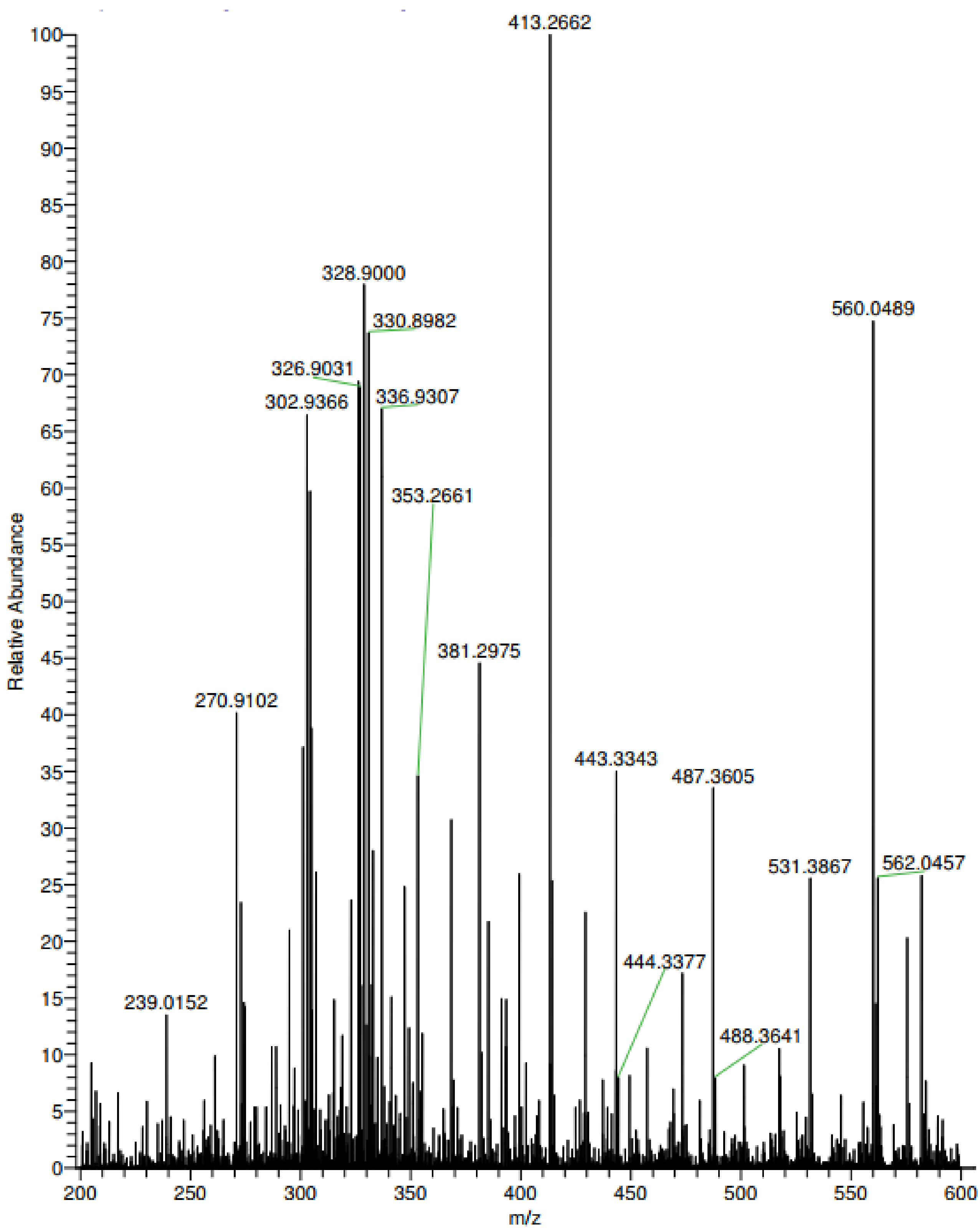

Figure S68. HRMS-ESI(+) of compound **TI45c**.

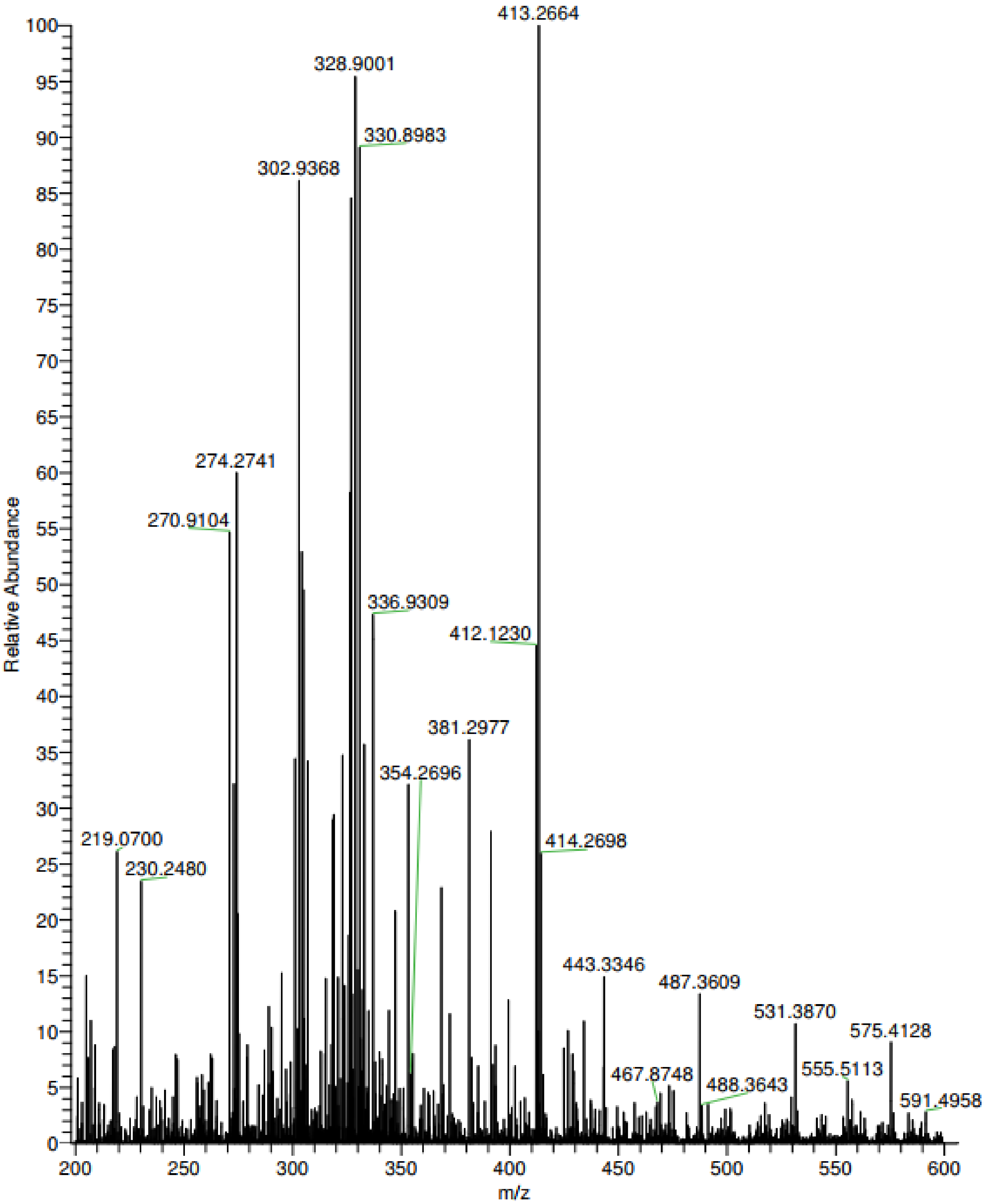

Figure S69. HRMS-ESI(+) of compound **Tl58a**.

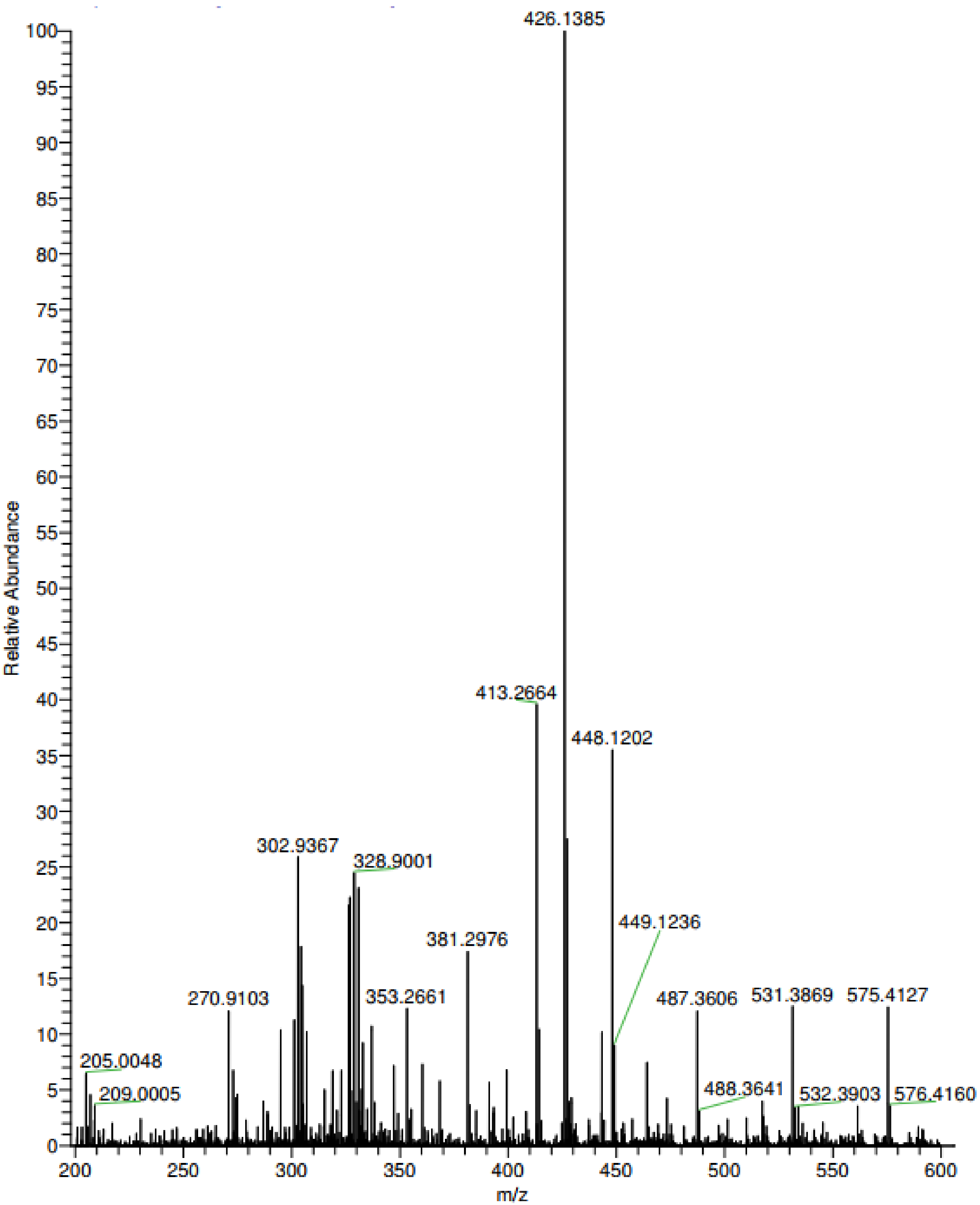

Figure S70. HRMS-ESI(+) of compound **Tl58b**.

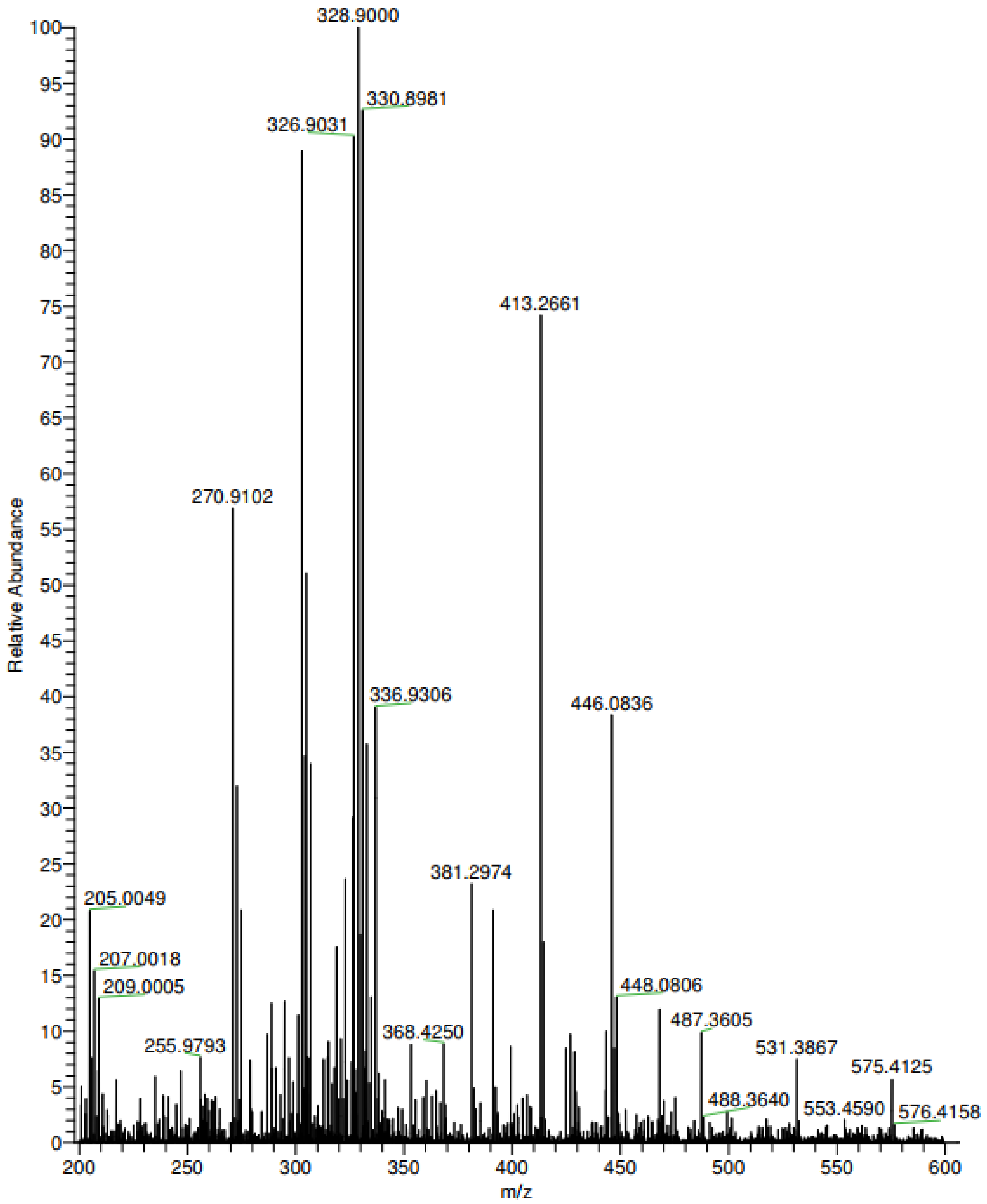

Figure S71. HRMS-ESI(+) of compound **Tl58c**.

4. Single-crystal X-ray diffraction

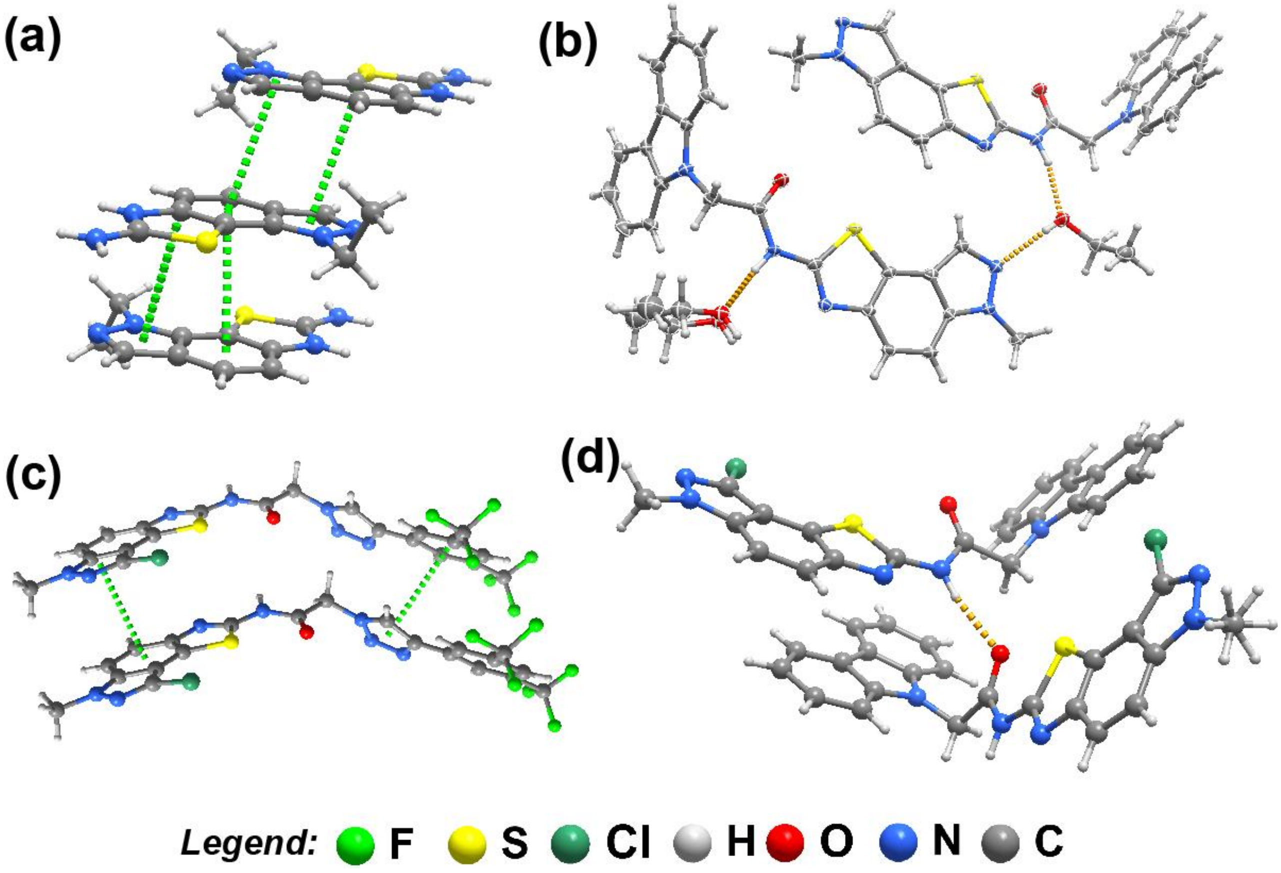

Figure 72. Schematic representation of: the **(a)** π-π interactions (dashed green lines) present in compound **5d** between the indazole rings; **(b)** hydrogen bonding interactions between the molecular units and the crystallization ethanol molecules (dashed orange lines) present in the crystal structures of compound **6b**; **(c)** π-π interaction (dashed green lines) between the indazole backbone and between the aromatic ring and the triazole residue of the neighboring molecular units in **TI45c; (d)** hydrogen bonding interactions present between two molecular units of **TI58c** (dashed orange lines).

5. Virtual library

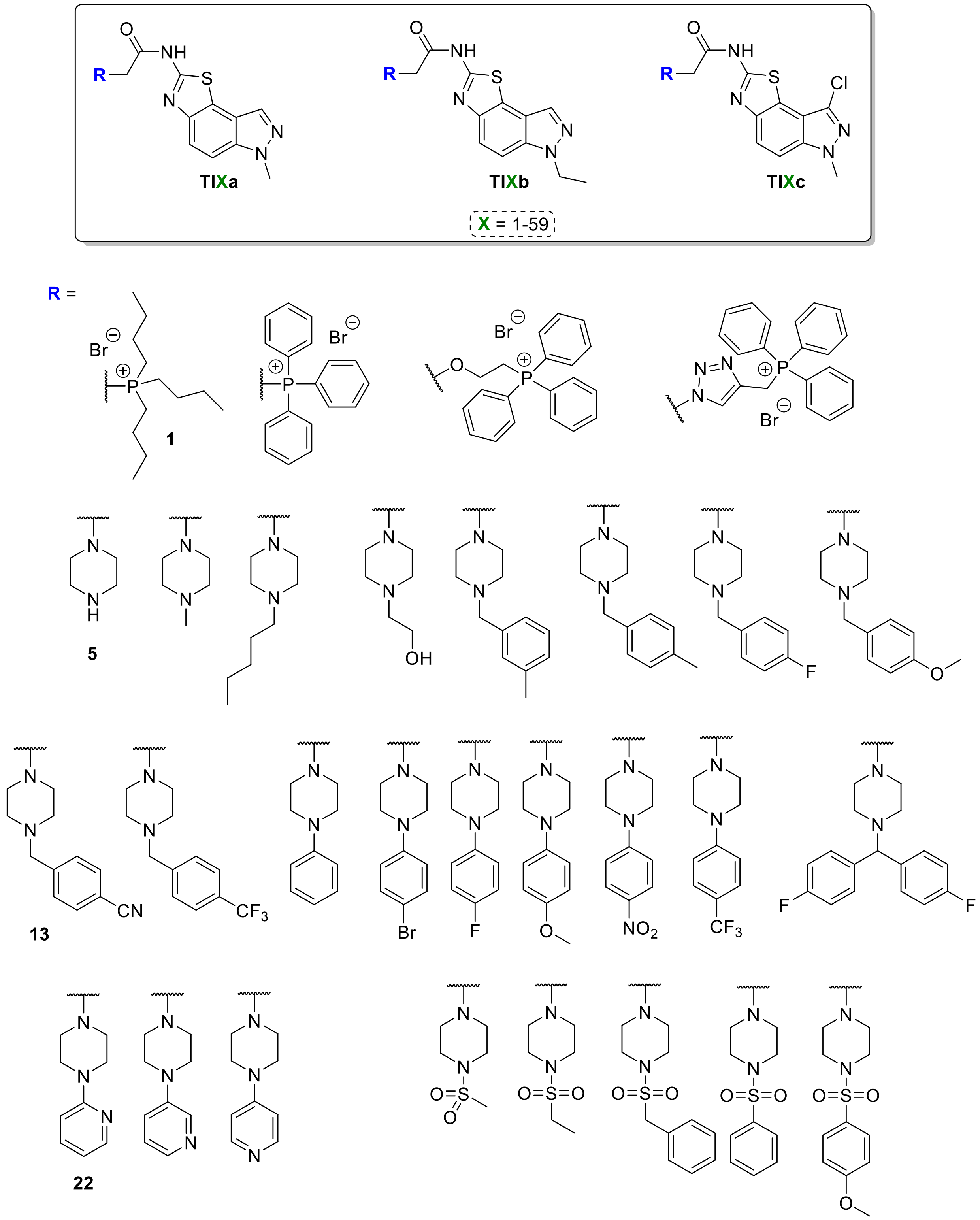

Figure S73. Virtual library of thiazoloindazole-based derivatives.

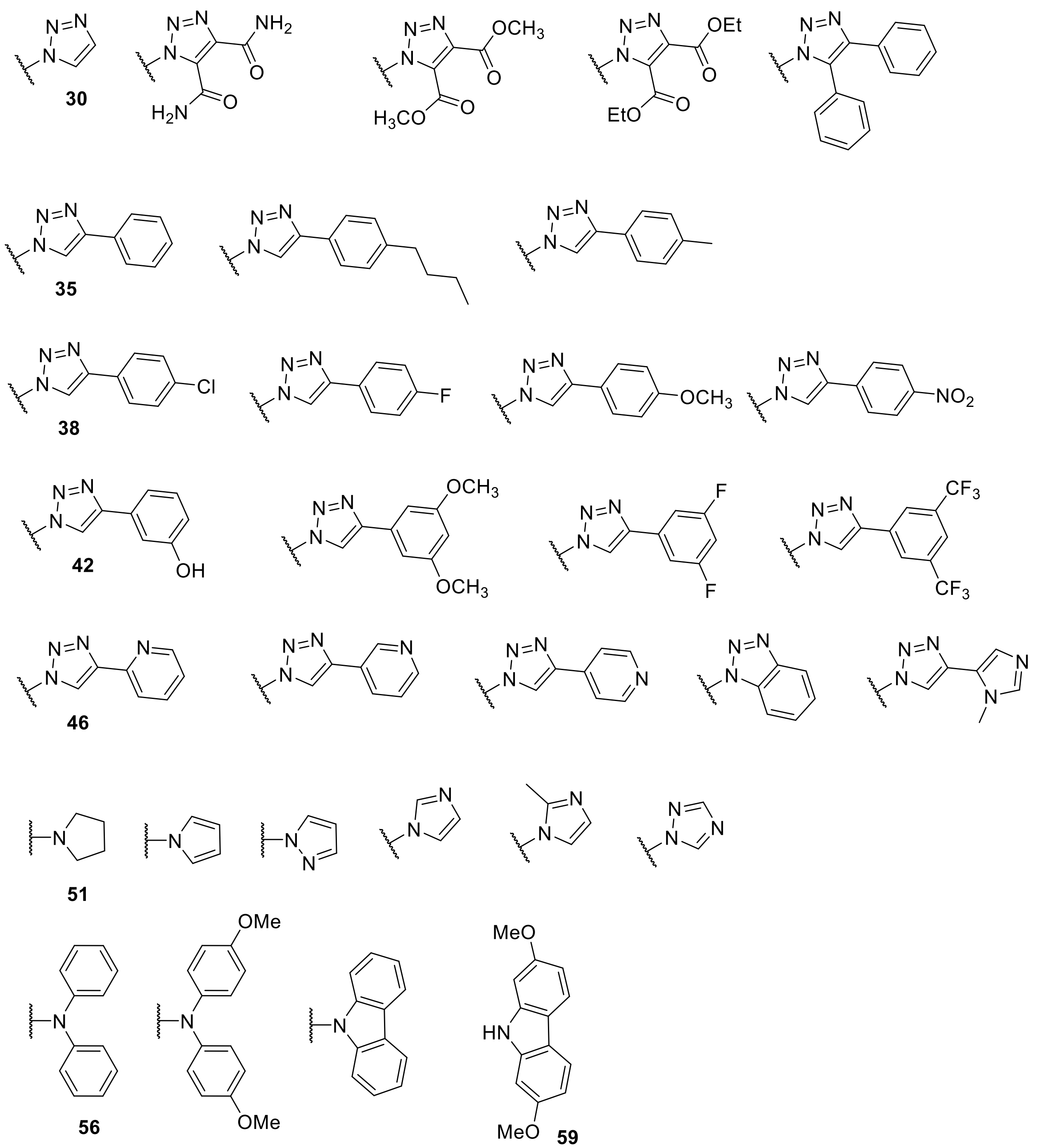

Figure S73. Virtual library of thiazoloindazole-based derivatives (cont.).

7. Molecular docking data against AChE enzyme

Table S1. Calculated free binding energies (ΔG$_B$, in kcal/mol) for the 188 thiazoloindazole-based derivatives and the positive control (donepezil) against AChE enzyme.

			Molecular docking against AChE enzyme
**Code**	**SMILES**	**Type**	**Calculated free binding energies (ΔGB, in kcal/mol)**
**5a**	CN1N=CC2=C3SC(N)=NC3=CC=C12	Synthesized	-7.5
**5b**	CCN1N=CC2=C3SC(N)=NC3=CC=C12	Synthesized	-7.9
**5c**	CN1N=C(Cl)C2=C3SC(N)=NC3=CC=C12	Synthesized	-7.9
**5d**	CN1N=CC2=CC=C3N=C(N)SC3=C12	Synthesized	-7.5
**5e**	CCN1N=CC2=CC=C3N=C(N)SC3=C12	Synthesized	-7.6
**6a**	CN1N=CC2=C3SC(NC(=O)CCl)=NC3=CC=C12	Synthesized	-7.4
**6b**	CCN1N=CC2=C3SC(NC(=O)CCl)=NC3=CC=C12	Synthesized	-7.7
**6c**	CN1N=C(Cl)C2=C3SC(NC(=O)CCl)=NC3=CC=C12	Synthesized	-8
**7a**	CN1C2=CC=C3C(SC(NC(CN=[N+]=[N-])=O)=N3)=C2C=N1	Synthesized	-8.1
**7b**	O=C(CN=[N+]=[N-])NC1=NC2=CC=C(N(CC)N=C3)C3=C2S1	Synthesized	-8.3
**7c**	CN1C2=CC=C3C(SC(NC(CN=[N+]=[N-])=O)=N3)=C2C(Cl)=N1	Synthesized	-8.7
**TI1a**	CCCC[P+](CCCC)(CCCC)CC(=O)NC1=NC2=CC=C3N(C)N=CC3=C2S1	Virtual_Library **TIXa**	-8.8
**TI2a**	CN1N=CC2=C3SC(NC(=O)C[P+](C4=CC=CC=C4)(C4=CC=CC=C4)C4=CC=CC=C4)=NC3=CC=C12	Virtual_Library **TIXa**	-8.9
**TI3a**	CN1N=CC2=C3SC(NC(=O)COCC[P+](C4=CC=CC=C4)(C4=CC=CC=C4)C4=CC=CC=C4)=NC3=CC=C12	Virtual_Library **TIXa**	-9.4
**TI4a**	CN1N=CC2=C3SC(NC(=O)CN4C=C(C[P+](C5=CC=CC=C5)(C5=CC=CC=C5)C5=CC=CC=C5)N=N4)=NC3=CC=C12	Virtual_Library **TIXa**	-8.9
**TI5a**	CN1N=CC2=C3SC(NC(=O)CN4CCNCC4)=NC3=CC=C12	Virtual_Library **TIXa**	-8.5
**TI6a**	CN1N=CC2=C3SC(NC(=O)CN4CCN(C)CC4)=NC3=CC=C12	Virtual_Library **TIXa**	-8.4
**TI7a**	CCCCCN1CCN(CC(=O)NC2=NC3=CC=C4N(C)N=CC4=C3S2)CC1	Virtual_Library **TIXa**	-7.5
**TI8a**	CN1N=CC2=C3SC(NC(=O)CN4CCN(CCO)CC4)=NC3=CC=C12	Virtual_Library **TIXa**	-9.1
**TI9a**	CN1N=CC2=C3SC(NC(=O)CN4CCN(CC5=CC=CC(C)=C5)CC4)=NC3=CC=C12	Virtual_Library **TIXa**	-11.1
**TI10a**	CN1N=CC2=C3SC(NC(=O)CN4CCN(CC5=CC=C(C)C=C5)CC4)=NC3=CC=C12	Virtual_Library **TIXa**	-10.8
**TI11a**	CN1N=CC2=C3SC(NC(=O)CN4CCN(CC5=CC=C(F)C=C5)CC4)=NC3=CC=C12	Virtual_Library **TIXa**	-10.6
**TI12a**	COC1=CC=C(CN2CCN(CC(=O)NC3=NC4=CC=C5N(C)N=CC5=C4S3)CC2)C=C1	Virtual_Library **TIXa**	-9.6
**TI13a**	CN1N=CC2=C3SC(NC(=O)CN4CCN(CC5=CC=C(C=C5)C#N)CC4)=NC3=CC=C12	Virtual_Library **TIXa**	-8.9
**TI14a**	CN1N=CC2=C3SC(NC(=O)CN4CCN(CC5=CC=C(C=C5)C(F)(F)F)CC4)=NC3=CC=C12	Virtual_Library **TIXa**	-10.6
**TI15a**	CN1N=CC2=C3SC(NC(=O)CN4CCN(CC4)C4=CC=CC=C4)=NC3=CC=C12	Virtual_Library **TIXa**	-9.7
**TI16a**	CN1N=CC2=C3SC(NC(=O)CN4CCN(CC4)C4=CC=C(Br)C=C4)=NC3=CC=C12	Virtual_Library **TIXa**	-10
**TI17a**	CN1N=CC2=C3SC(NC(=O)CN4CCN(CC4)C4=CC=C(F)C=C4)=NC3=CC=C12	Virtual_Library **TIXa**	-10.5
**TI18a**	COC1=CC=C(C=C1)N1CCN(CC(=O)NC2=NC3=CC=C4N(C)N=CC4=C3S2)CC1	Virtual_Library **TIXa**	-9.7
**TI19a**	CN1N=CC2=C3SC(NC(=O)CN4CCN(CC4)C4=CC=C(C=C4)[N+]([O-])=O)=NC3=CC=C12	Virtual_Library **TIXa**	-9.8
**TI20a**	CN1N=CC2=C3SC(NC(=O)CN4CCN(CC4)C4=CC=C(C=C4)C(F)(F)F)=NC3=CC=C12	Virtual_Library **TIXa**	-10.0

**TI21a**	CN1N=CC2=C3SC(NC(=O)CN4CCN(CC4)C(C4=CC=C(F)C=C4)C4=CC=C(F)C=C4)=NC3=CC=C12	Virtual_Library **TIXa**	-11.5
**TI22a**	CN1N=CC2=C3SC(NC(=O)CN4CCN(CC4)C4=NC=CC=C4)=NC3=CC=C12	Virtual_Library **TIXa**	-10.4
**TI23a**	CN1N=CC2=C3SC(NC(=O)CN4CCN(CC4)C4=CN=CC=C4)=NC3=CC=C12	Virtual_Library **TIXa**	-10.3
**TI24a**	CN1N=CC2=C3SC(NC(=O)CN4CCN(CC4)C4=CC=NC=C4)=NC3=CC=C12	Virtual_Library **TIXa**	-10.0
**TI25a**	CN1N=CC2=C3SC(NC(=O)CN4CCN(CC4)S(C)(=O)=O)=NC3=CC=C12	Virtual_Library **TIXa**	-8.8
**TI26a**	CCS(=O)(=O)N1CCN(CC(=O)NC2=NC3=CC=C4N(C)N=CC4=C3S2)CC1	Virtual_Library **TIXa**	-8.3
**TI27a**	CN1N=CC2=C3SC(NC(=O)CN4CCN(CC4)S(=O)(=O)CC4=CC=CC=C4)=NC3=CC=C12	Virtual_Library **TIXa**	-11.1
**TI28a**	CN1N=CC2=C3SC(NC(=O)CN4CCN(CC4)S(=O)(=O)C4=CC=CC=C4)=NC3=CC=C12	Virtual_Library **TIXa**	-11.3
**TI29a**	COC1=CC=C(C=C1)S(=O)(=O)N1CCN(CC(=O)NC2=NC3=CC=C4N(C)N=CC4=C3S2)CC1	Virtual_Library **TIXa**	-10.0
**TI30a**	CN1N=CC2=C3SC(NC(=O)CN4C=CN=N4)=NC3=CC=C12	Virtual_Library **TIXa**	-9.4
**TI31a**	CN1N=CC2=C3SC(NC(=O)CN4N=NC(C(N)=O)=C4C(N)=O)=NC3=CC=C12	Virtual_Library **TIXa**	-10.8
**TI32a**	COC(=O)C1=C(N(CC(=O)NC2=NC3=CC=C4N(C)N=CC4=C3S2)N=N1)C(=O)OC	Virtual_Library **TIXa**	-10.3
**TI33a**	CCOC(=O)C1=C(N(CC(=O)NC2=NC3=CC=C4N(C)N=CC4=C3S2)N=N1)C(=O)OCC	Virtual_Library **TIXa**	-7.7
**TI34a**	CN1N=CC2=C3SC(NC(=O)CN4N=NC(=C4C4=CC=CC=C4)C4=CC=CC=C4)=NC3=CC=C12	Virtual_Library **TIXa**	-11.8
**TI35a**	CN1N=CC2=C3SC(NC(=O)CN4C=C(N=N4)C4=CC=CC=C4)=NC3=CC=C12	Virtual_Library **TIXa**	-10.6
**TI36a**	CCCCC1=CC=C(C=C1)C1=CN(CC(=O)NC2=NC3=CC=C4N(C)N=CC4=C3S2)N=N1	Virtual_Library **TIXa**	-10.1
**TI37a**	CN1N=CC2=C3SC(NC(=O)CN4C=C(N=N4)C4=CC=C(C)C=C4)=NC3=CC=C12	Virtual_Library **TIXa**	-10.9
**TI38a**	CN1N=CC2=C3SC(NC(=O)CN4C=C(N=N4)C4=CC=C(Cl)C=C4)=NC3=CC=C12	Virtual_Library **TIXa**	-11.1
**TI39a**	CN1N=CC2=C3SC(NC(=O)CN4C=C(N=N4)C4=CC=C(F)C=C4)=NC3=CC=C12	Virtual_Library **TIXa**	-10.9
**TI40a**	COC1=CC=C(C=C1)C1=CN(CC(=O)NC2=NC3=CC=C4N(C)N=CC4=C3S2)N=N1	Virtual_Library **TIXa**	-10.3
**TI41a**	CN1N=CC2=C3SC(NC(=O)CN4C=C(N=N4)C4=CC=C(C=C4)[N+]([O-])=O)=NC3=CC=C12	Virtual_Library **TIXa**	-11.0
**TI42a**	CN1N=CC2=C3SC(NC(=O)CN4C=C(N=N4)C4=CC=CC(O)=C4)=NC3=CC=C12	Virtual_Library **TIXa**	-10.8
**TI43a**	COC1=CC(=CC(OC)=C1)C1=CN(CC(=O)NC2=NC3=CC=C4N(C)N=CC4=C3S2)N=N1	Virtual_Library **TIXa**	-10.9
**TI44a**	CN1N=CC2=C3SC(NC(=O)CN4C=C(N=N4)C4=CC(F)=CC(F)=C4)=NC3=CC=C12	Virtual_Library **TIXa**	-11.2
**TI45a**	CN1N=CC2=C3SC(NC(=O)CN4C=C(N=N4)C4=CC(=CC(=C4)C(F)(F)F)C(F)(F)F)=NC3=CC=C12	Virtual_Library **TIXa**	-12.7
**TI46a**	CN1N=CC2=C3SC(NC(=O)CN4C=C(N=N4)C4=NC=CC=C4)=NC3=CC=C12	Virtual_Library **TIXa**	-10.5
**TI47a**	CN1N=CC2=C3SC(NC(=O)CN4C=C(N=N4)C4=CN=CC=C4)=NC3=CC=C12	Virtual_Library **TIXa**	-10.5
**TI48a**	CN1N=CC2=C3SC(NC(=O)CN4C=C(N=N4)C4=CC=NC=C4)=NC3=CC=C12	Virtual_Library **TIXa**	-10.3
**TI49a**	CN1N=CC2=C3SC(NC(=O)CN4N=NC5=C4C=CC=C5)=NC3=CC=C12	Virtual_Library **TIXa**	-10.9
**TI50a**	CN1C=NC=C1C1=CN(CC(=O)NC2=NC3=CC=C4N(C)N=CC4=C3S2)N=N1	Virtual_Library **TIXa**	-10.2
**TI51a**	CN1N=CC2=C3SC(NC(=O)CN4CCCC4)=NC3=CC=C12	Virtual_Library **TIXa**	-8.6
**TI52a**	CN1N=CC2=C3SC(NC(=O)CN4C=CC=C4)=NC3=CC=C12	Virtual_Library **TIXa**	-9.2
**TI53a**	CN1N=CC2=C3SC(NC(=O)CN4C=CC=N4)=NC3=CC=C12	Virtual_Library **TIXa**	-8.6
**TI54a**	CN1N=CC2=C3SC(NC(=O)CN4C=CN=C4)=NC3=CC=C12	Virtual_Library **TIXa**	-7.6

**TI55a**	CN1N=CC2=C3SC(NC(=O)CN4C=NC=N4)=NC3=CC=C12	Virtual_Library **TIXa**	-7.7
**TI56a**	CN1N=CC2=C3SC(NC(=O)CN(C4=CC=CC=C4)C4=CC=CC=C4)=NC3=CC=C12	Virtual_Library **TIXa**	-10.4
**TI57a**	COC1=CC=C(C=C1)N(CC(=O)NC1=NC2=CC=C3N(C)N=CC3=C2S1)C1=CC=C(OC)C=C1	Virtual_Library **TIXa**	-9.7
**TI58a**	CN1N=CC2=C3SC(NC(=O)CN4C5=C(C=CC=C5)C5=C4C=CC=C5)=NC3=CC=C12	Virtual_Library **TIXa**	-11.7
**TI59a**	COC1=CC2=C(C=C1)C1=C(C=C(OC)C=C1)N2CC(=O)NC1=NC2=CC=C3N(C)N=CC3=C2S1	Virtual_Library **TIXa**	-10.5
**TI1b**	CCCC[P+](CCCC)(CCCC)CC(=O)NC1=NC2=CC=C3N(CC)N=CC3=C2S1	Virtual_Library **TIXb**	-8.8
**TI2b**	CCN1N=CC2=C3SC(NC(=O)C[P+](C4=CC=CC=C4)(C4=CC=CC=C4)C4=CC=CC=C4)=NC3=CC=C12	Virtual_Library **TIXb**	-8.7
**TI3b**	CCN1N=CC2=C3SC(NC(=O)COCC[P+](C4=CC=CC=C4)(C4=CC=CC=C4)C4=CC=CC=C4)=NC3=CC=C12	Virtual_Library **TIXb**	-9.7
**TI4b**	CCN1N=CC2=C3SC(NC(=O)CN4C=C(C[P+](C5=CC=CC=C5)(C5=CC=CC=C5)C5=CC=CC=C5)N=N4)=NC3=CC=C12	Virtual_Library **TIXb**	-8.7
**TI5b**	CCN1N=CC2=C3SC(NC(=O)CN4CCNCC4)=NC3=CC=C12	Virtual_Library **TIXb**	-8
**TI6b**	CCN1N=CC2=C3SC(NC(=O)CN4CCN(C)CC4)=NC3=CC=C12	Virtual_Library **TIXb**	-9.2
**TI7b**	CCCCCN1CCN(CC(=O)NC2=NC3=CC=C4N(CC)N=CC4=C3S2)CC1	Virtual_Library **TIXb**	-7.7
**TI8b**	CCN1N=CC2=C3SC(NC(=O)CN4CCN(CCO)CC4)=NC3=CC=C12	Virtual_Library **TIXb**	-8.9
**TI9b**	CCN1N=CC2=C3SC(NC(=O)CN4CCN(CC5=CC=CC(C)=C5)CC4)=NC3=CC=C12	Virtual_Library **TIXb**	-9.1
**TI10b**	CCN1N=CC2=C3SC(NC(=O)CN4CCN(CC5=CC=C(C)C=C5)CC4)=NC3=CC=C12	Virtual_Library **TIXb**	-10.5
**TI11b**	CCN1N=CC2=C3SC(NC(=O)CN4CCN(CC5=CC=C(F)C=C5)CC4)=NC3=CC=C12	Virtual_Library **TIXb**	-10.6
**TI12b**	CCN1N=CC2=C3SC(NC(=O)CN4CCN(CC5=CC=C(OC)C=C5)CC4)=NC3=CC=C12	Virtual_Library **TIXb**	-9.8
**TI13b**	CCN1N=CC2=C3SC(NC(=O)CN4CCN(CC5=CC=C(C=C5)C#N)CC4)=NC3=CC=C12	Virtual_Library **TIXb**	-10.2
**TI14b**	CCN1N=CC2=C3SC(NC(=O)CN4CCN(CC5=CC=C(C=C5)C(F)(F)F)CC4)=NC3=CC=C12	Virtual_Library **TIXb**	-9.2
**TI15b**	CCN1N=CC2=C3SC(NC(=O)CN4CCN(CC4)C4=CC=CC=C4)=NC3=CC=C12	Virtual_Library **TIXb**	-10.4
**TI16b**	CCN1N=CC2=C3SC(NC(=O)CN4CCN(CC4)C4=CC=C(Br)C=C4)=NC3=CC=C12	Virtual_Library **TIXb**	-9.8
**TI17b**	CCN1N=CC2=C3SC(NC(=O)CN4CCN(CC4)C4=CC=C(F)C=C4)=NC3=CC=C12	Virtual_Library **TIXb**	-10.5
**TI18b**	CCN1N=CC2=C3SC(NC(=O)CN4CCN(CC4)C4=CC=C(OC)C=C4)=NC3=CC=C12	Virtual_Library **TIXb**	-8.7
**TI19b**	CCN1N=CC2=C3SC(NC(=O)CN4CCN(CC4)C4=CC=C(C=C4)[N+]([O-])=O)=NC3=CC=C12	Virtual_Library **TIXb**	-9.0
**TI20b**	CCN1N=CC2=C3SC(NC(=O)CN4CCN(CC4)C4=CC=C(C=C4)C(F)(F)F)=NC3=CC=C12	Virtual_Library **TIXb**	-9.2
**TI21b**	CCN1N=CC2=C3SC(NC(=O)CN4CCN(CC4)C(C4=CC=C(F)C=C4)C4=CC=C(F)C=C4)=NC3=CC=C12	Virtual_Library **TIXb**	-9.4
**TI22b**	CCN1N=CC2=C3SC(NC(=O)CN4CCN(CC4)C4=NC=CC=C4)=NC3=CC=C12	Virtual_Library **TIXb**	-10.1
**TI23b**	CCN1N=CC2=C3SC(NC(=O)CN4CCN(CC4)C4=CN=CC=C4)=NC3=CC=C12	Virtual_Library **TIXb**	-8.5
**TI24b**	CCN1N=CC2=C3SC(NC(=O)CN4CCN(CC4)C4=CC=NC=C4)=NC3=CC=C12	Virtual_Library **TIXb**	-9.8
**TI25b**	CCN1N=CC2=C3SC(NC(=O)CN4CCN(CC4)S(C)(=O)=O)=NC3=CC=C12	Virtual_Library **TIXb**	-8.7
**TI26b**	CCN1N=CC2=C3SC(NC(=O)CN4CCN(CC4)S(=O)(=O)CC)=NC3=CC=C12	Virtual_Library **TIXb**	-9.5
**TI27b**	CCN1N=CC2=C3SC(NC(=O)CN4CCN(CC4)S(=O)(=O)CC4=CC=CC=C4)=NC3=CC=C12	Virtual_Library **TIXb**	-10.8
**TI28b**	CCN1N=CC2=C3SC(NC(=O)CN4CCN(CC4)S(=O)(=O)C4=CC=CC=C4)=NC3=CC=C12	Virtual_Library **TIXb**	-11.3
**TI29b**	CCN1N=CC2=C3SC(NC(=O)CN4CCN(CC4)S(=O)(=O)C4=CC=C(OC)C=C4)=NC3=CC=C12	Virtual_Library **TIXb**	-8.6

**TI30b**	CCN1N=CC2=C3SC(NC(=O)CN4C=CN=N4)=NC3=CC=C12	Virtual_Library **TIXb**	-8.9
**TI31b**	CCN1N=CC2=C3SC(NC(=O)CN4N=NC(C(N)=O)=C4C(N)=O)=NC3=CC=C12	Virtual_Library **TIXb**	-10.6
**TI32b**	CCN1N=CC2=C3SC(NC(=O)CN4N=NC(C(=O)OC)=C4C(=O)OC)=NC3=CC=C12	Virtual_Library **TIXb**	-10.2
**TI33b**	CCOC(=O)C1=C(N(CC(=O)NC2=NC3=CC=C4N(CC)N=CC4=C3S2)N=N1)C(=O)OCC	Virtual_Library **TIXb**	-9.6
**TI34b**	CCN1N=CC2=C3SC(NC(=O)CN4N=NC(=C4C4=CC=CC=C4)C4=CC=CC=C4)=NC3=CC=C12	Virtual_Library **TIXb**	-10.0
**TI35b**	CCN1N=CC2=C3SC(NC(=O)CN4C=C(N=N4)C4=CC=CC=C4)=NC3=CC=C12	Virtual_Library **TIXb**	-10.4
**TI36b**	CCCCC1=CC=C(C=C1)C1=CN(CC(=O)NC2=NC3=CC=C4N(CC)N=CC4=C3S2)N=N1	Virtual_Library **TIXb**	-9.9
**TI37b**	CCN1N=CC2=C3SC(NC(=O)CN4C=C(N=N4)C4=CC=C(C)C=C4)=NC3=CC=C12	Virtual_Library **TIXb**	-10.5
**TI38b**	CCN1N=CC2=C3SC(NC(=O)CN4C=C(N=N4)C4=CC=C(Cl)C=C4)=NC3=CC=C12	Virtual_Library **TIXb**	-10.6
**TI39b**	CCN1N=CC2=C3SC(NC(=O)CN4C=C(N=N4)C4=CC=C(F)C=C4)=NC3=CC=C12	Virtual_Library **TIXb**	-10.6
**TI40b**	CCN1N=CC2=C3SC(NC(=O)CN4C=C(N=N4)C4=CC=C(OC)C=C4)=NC3=CC=C12	Virtual_Library **TIXb**	-9.8
**TI41b**	CCN1N=CC2=C3SC(NC(=O)CN4C=C(N=N4)C4=CC=C(C=C4)[N+]([O-])=O)=NC3=CC=C12	Virtual_Library **TIXb**	-10.3
**TI42b**	CCN1N=CC2=C3SC(NC(=O)CN4C=C(N=N4)C4=CC=CC(O)=C4)=NC3=CC=C12	Virtual_Library **TIXb**	-10.8
**TI43b**	CCN1N=CC2=C3SC(NC(=O)CN4C=C(N=N4)C4=CC(OC)=CC(OC)=C4)=NC3=CC=C12	Virtual_Library **TIXb**	-10.6
**TI44b**	CCN1N=CC2=C3SC(NC(=O)CN4C=C(N=N4)C4=CC(F)=CC(F)=C4)=NC3=CC=C12	Virtual_Library **TIXb**	-10.6
**TI45b**	CCN1N=CC2=C3SC(NC(=O)CN4C=C(N=N4)C4=CC(=CC(=C4)C(F)(F)F)C(F)(F)F)=NC3=CC=C12	Virtual_Library **TIXb**	-12.3
**TI46b**	CCN1N=CC2=C3SC(NC(=O)CN4C=C(N=N4)C4=NC=CC=C4)=NC3=CC=C12	Virtual_Library **TIXb**	-10.0
**TI47b**	CCN1N=CC2=C3SC(NC(=O)CN4C=C(N=N4)C4=CN=CC=C4)=NC3=CC=C12	Virtual_Library **TIXb**	-10.0
**TI48b**	CCN1N=CC2=C3SC(NC(=O)CN4C=C(N=N4)C4=CC=NC=C4)=NC3=CC=C12	Virtual_Library **TIXb**	-10.5
**TI49b**	CCN1N=CC2=C3SC(NC(=O)CN4N=NC5=C4C=CC=C5)=NC3=CC=C12	Virtual_Library **TIXb**	-9.9
**TI50b**	CCN1N=CC2=C3SC(NC(=O)CN4C=C(N=N4)C4=CN=CN4C)=NC3=CC=C12	Virtual_Library **TIXb**	-10.3
**TI51b**	CCN1N=CC2=C3SC(NC(=O)CN4CCCC4)=NC3=CC=C12	Virtual_Library **TIXb**	-8.7
**TI52b**	CCN1N=CC2=C3SC(NC(=O)CN4C=CC=C4)=NC3=CC=C12	Virtual_Library **TIXb**	-9.1
**TI53b**	CCN1N=CC2=C3SC(NC(=O)CN4C=CC=N4)=NC3=CC=C12	Virtual_Library **TIXb**	-8.5
**TI54b**	CCN1N=CC2=C3SC(NC(=O)CN4C=CN=C4)=NC3=CC=C12	Virtual_Library **TIXb**	-8.9
**TI55b**	CCN1N=CC2=C3SC(NC(=O)CN4C=NC=N4)=NC3=CC=C12	Virtual_Library **TIXb**	-8.6
**TI56b**	CCN1N=CC2=C3SC(NC(=O)CN(C4=CC=CC=C4)C4=CC=CC=C4)=NC3=CC=C12	Virtual_Library **TIXb**	-10.7
**TI57b**	CCN1N=CC2=C3SC(NC(=O)CN(C4=CC=C(OC)C=C4)C4=CC=C(OC)C=C4)=NC3=CC=C12	Virtual_Library **TIXb**	-9.4
**TI58b**	CCN1N=CC2=C3SC(NC(=O)CN4C5=C(C=CC=C5)C5=C4C=CC=C5)=NC3=CC=C12	Virtual_Library **TIXb**	-11.6
**TI59b**	CCN1N=CC2=C3SC(NC(=O)CN4C5=C(C=CC(OC)=C5)C5=C4C=C(OC)C=C5)=NC3=CC=C12	Virtual_Library **TIXb**	-9.5
**TI1c**	CCCC[P+](CCCC)(CCCC)CC(=O)NC1=NC2=CC=C3N(C)N=C(Cl)C3=C2S1	Virtual_Library **TIXc**	-8.7
**TI2c**	CN1N=C(Cl)C2=C3SC(NC(=O)C[P+](C4=CC=CC=C4)(C4=CC=CC=C4)C4=CC=CC=C4)=NC3=CC=C12	Virtual_Library **TIXc**	-9.5
**TI3c**	CN1N=C(Cl)C2=C3SC(NC(=O)COCC[P+](C4=CC=CC=C4)(C4=CC=CC=C4)C4=CC=CC=C4)=NC3=CC=C12	Virtual_Library **TIXc**	-10.3
**TI4c**	CN1N=C(Cl)C2=C3SC(NC(=O)CN4C=C(C[P+](C5=CC=CC=C5)(C5=CC=CC=C5)C5=CC=CC=C5)N=N4)=NC3=CC=C12	Virtual_Library **TIXc**	-10.0

**TI5c**	CN1N=C(Cl)C2=C3SC(NC(=O)CN4CCNCC4)=NC3=CC=C12	Virtual_Library **TIXc**	-8.9
**TI6c**	CN1N=C(Cl)C2=C3SC(NC(=O)CN4CCN(C)CC4)=NC3=CC=C12	Virtual_Library **TIXc**	-7.1
**TI7c**	CCCCCN1CCN(CC(=O)NC2=NC3=CC=C4N(C)N=C(Cl)C4=C3S2)CC1	Virtual_Library **TIXc**	-7.5
**TI8c**	CN1N=C(Cl)C2=C3SC(NC(=O)CN4CCN(CCO)CC4)=NC3=CC=C12	Virtual_Library **TIXc**	-9.4
**TI9c**	CN1N=C(Cl)C2=C3SC(NC(=O)CN4CCN(CC5=CC=CC(C)=C5)CC4)=NC3=CC=C12	Virtual_Library **TIXc**	-10.2
**TI10c**	CN1N=C(Cl)C2=C3SC(NC(=O)CN4CCN(CC5=CC=C(C)C=C5)CC4)=NC3=CC=C12	Virtual_Library **TIXc**	-10.1
**TI11c**	CN1N=C(Cl)C2=C3SC(NC(=O)CN4CCN(CC5=CC=C(F)C=C5)CC4)=NC3=CC=C12	Virtual_Library **TIXc**	-11.1
**TI12c**	COC1=CC=C(CN2CCN(CC(=O)NC3=NC4=CC=C5N(C)N=C(Cl)C5=C4S3)CC2)C=C1	Virtual_Library **TIXc**	-9.5
**TI13c**	CN1N=C(Cl)C2=C3SC(NC(=O)CN4CCN(CC5=CC=C(C=C5)C#N)CC4)=NC3=CC=C12	Virtual_Library **TIXc**	-9.1
**TI14c**	CN1N=C(Cl)C2=C3SC(NC(=O)CN4CCN(CC5=CC=C(C=C5)C(F)(F)F)CC4)=NC3=CC=C12	Virtual_Library **TIXc**	-10.9
**TI15c**	CN1N=C(Cl)C2=C3SC(NC(=O)CN4CCN(CC4)C4=CC=CC=C4)=NC3=CC=C12	Virtual_Library **TIXc**	-11.1
**TI16c**	CN1N=C(Cl)C2=C3SC(NC(=O)CN4CCN(CC4)C4=CC=C(Br)C=C4)=NC3=CC=C12	Virtual_Library **TIXc**	-10.1
**TI17c**	CN1N=C(Cl)C2=C3SC(NC(=O)CN4CCN(CC4)C4=CC=C(F)C=C4)=NC3=CC=C12	Virtual_Library **TIXc**	-9.3
**TI18c**	COC1=CC=C(C=C1)N1CCN(CC(=O)NC2=NC3=CC=C4N(C)N=C(Cl)C4=C3S2)CC1	Virtual_Library **TIXc**	-9.3
**TI19c**	CN1N=C(Cl)C2=C3SC(NC(=O)CN4CCN(CC4)C4=CC=C(C=C4)[N+]([O-])=O)=NC3=CC=C12	Virtual_Library **TIXc**	-9.4
**TI20c**	CN1N=C(Cl)C2=C3SC(NC(=O)CN4CCN(CC4)C4=CC=C(C=C4)C(F)(F)F)=NC3=CC=C12	Virtual_Library **TIXc**	-10.0
**TI21c**	CN1N=C(Cl)C2=C3SC(NC(=O)CN4CCN(CC4)C(C4=CC=C(F)C=C4)C4=CC=C(F)C=C4)=NC3=CC=C12	Virtual_Library **TIXc**	-10.1
**TI22c**	CN1N=C(Cl)C2=C3SC(NC(=O)CN4CCN(CC4)C4=NC=CC=C4)=NC3=CC=C12	Virtual_Library **TIXc**	-10.4
**TI23c**	CN1N=C(Cl)C2=C3SC(NC(=O)CN4CCN(CC4)C4=CN=CC=C4)=NC3=CC=C12	Virtual_Library **TIXc**	-9.2
**TI24c**	CN1N=C(Cl)C2=C3SC(NC(=O)CN4CCN(CC4)C4=CC=NC=C4)=NC3=CC=C12	Virtual_Library **TIXc**	-10.1
**TI25c**	CN1N=C(Cl)C2=C3SC(NC(=O)CN4CCN(CC4)S(C)(=O)=O)=NC3=CC=C12	Virtual_Library **TIXc**	-9.0
**TI26c**	CCS(=O)(=O)N1CCN(CC(=O)NC2=NC3=CC=C4N(C)N=C(Cl)C4=C3S2)CC1	Virtual_Library **TIXc**	-8.6
**TI27c**	CN1N=C(Cl)C2=C3SC(NC(=O)CN4CCN(CC4)S(=O)(=O)CC4=CC=CC=C4)=NC3=CC=C12	Virtual_Library **TIXc**	-10.4
**TI28c**	CN1N=C(Cl)C2=C3SC(NC(=O)CN4CCN(CC4)S(=O)(=O)C4=CC=CC=C4)=NC3=CC=C12	Virtual_Library **TIXc**	-11.1
**TI29c**	COC1=CC=C(C=C1)S(=O)(=O)N1CCN(CC(=O)NC2=NC3=CC=C4N(C)N=C(Cl)C4=C3S2)CC1	Virtual_Library **TIXc**	-10.9
**TI30c**	CN1N=C(Cl)C2=C3SC(NC(=O)CN4C=CN=N4)=NC3=CC=C12	Virtual_Library **TIXc**	-9.1
**TI31c**	CN1N=C(Cl)C2=C3SC(NC(=O)CN4N=NC(C(N)=O)=C4C(N)=O)=NC3=CC=C12	Virtual_Library **TIXc**	-10.9
**TI32c**	COC(=O)C1=C(N(CC(=O)NC2=NC3=CC=C4N(C)N=C(Cl)C4=C3S2)N=N1)C(=O)OC	Virtual_Library **TIXc**	-10.3
**TI33c**	CCOC(=O)C1=C(N(CC(=O)NC2=NC3=CC=C4N(C)N=C(Cl)C4=C3S2)N=N1)C(=O)OCC	Virtual_Library **TIXc**	-9.5
**TI34c**	CN1N=C(Cl)C2=C3SC(NC(=O)CN4N=NC(=C4C4=CC=CC=C4)C4=CC=CC=C4)=NC3=CC=C12	Virtual_Library **TIXc**	-12.0
**TI35c**	CN1N=C(Cl)C2=C3SC(NC(=O)CN4C=C(N=N4)C4=CC=CC=C4)=NC3=CC=C12	Virtual_Library **TIXc**	-10.9
**TI36c**	CCCCC1=CC=C(C=C1)C1=CN(CC(=O)NC2=NC3=CC=C4N(C)N=C(Cl)C4=C3S2)N=N1	Virtual_Library **TIXc**	-10.5
**TI37c**	CN1N=C(Cl)C2=C3SC(NC(=O)CN4C=C(N=N4)C4=CC=C(C)C=C4)=NC3=CC=C12	Virtual_Library **TIXc**	-11.1
**TI38c**	CN1N=C(Cl)C2=C3SC(NC(=O)CN4C=C(N=N4)C4=CC=C(Cl)C=C4)=NC3=CC=C12	Virtual_Library **TIXc**	-9.2

**TI39c**	CN1N=C(Cl)C2=C3SC(NC(=O)CN4C=C(N=N4)C4=CC=C(F)C=C4)=NC3=CC=C12	Virtual_Library **TIXc**	-11.0
**TI40c**	COC1=CC=C(C=C1)C1=CN(CC(=O)NC2=NC3=CC=C4N(C)N=C(Cl)C4=C3S2)N=N1	Virtual_Library **TIXc**	-10.3
**TI41c**	CN1N=C(Cl)C2=C3SC(NC(=O)CN4C=C(N=N4)C4=CC=C(C=C4)[N+]([O-])=O)=NC3=CC=C12	Virtual_Library **TIXc**	-11.0
**TI42c**	CN1N=C(Cl)C2=C3SC(NC(=O)CN4C=C(N=N4)C4=CC=CC(O)=C4)=NC3=CC=C12	Virtual_Library **TIXc**	-10.4
**TI43c**	COC1=CC(=CC(OC)=C1)C1=CN(CC(=O)NC2=NC3=CC=C4N(C)N=C(Cl)C4=C3S2)N=N1	Virtual_Library **TIXc**	-11.0
**TI44c**	CN1N=C(Cl)C2=C3SC(NC(=O)CN4C=C(N=N4)C4=CC(F)=CC(F)=C4)=NC3=CC=C12	Virtual_Library **TIXc**	-11.5
**TI45c**	CN1N=C(Cl)C2=C3SC(NC(=O)CN4C=C(N=N4)C4=CC(=CC(=C4)C(F)(F)F)C(F)(F)F)=NC3=CC=C12	Virtual_Library **TIXc**	-12.8
**TI46c**	CN1N=C(Cl)C2=C3SC(NC(=O)CN4C=C(N=N4)C4=NC=CC=C4)=NC3=CC=C12	Virtual_Library **TIXc**	-9.4
**TI47c**	CN1N=C(Cl)C2=C3SC(NC(=O)CN4C=C(N=N4)C4=CN=CC=C4)=NC3=CC=C12	Virtual_Library **TIXc**	-10.8
**TI48c**	CN1N=C(Cl)C2=C3SC(NC(=O)CN4C=C(N=N4)C4=CC=NC=C4)=NC3=CC=C12	Virtual_Library **TIXc**	-10.7
**TI49c**	CN1N=C(Cl)C2=C3SC(NC(=O)CN4N=NC5=C4C=CC=C5)=NC3=CC=C12	Virtual_Library **TIXc**	-11.1
**TI50c**	CN1C=NC=C1C1=CN(CC(=O)NC2=NC3=CC=C4N(C)N=C(Cl)C4=C3S2)N=N1	Virtual_Library **TIXc**	-10.5
**TI51c**	CN1N=C(Cl)C2=C3SC(NC(=O)CN4CCCC4)=NC3=CC=C12	Virtual_Library **TIXc**	-8.2
**TI52c**	CN1N=C(Cl)C2=C3SC(NC(=O)CN4C=CC=C4)=NC3=CC=C12	Virtual_Library **TIXc**	-8.2
**TI53c**	CN1N=C(Cl)C2=C3SC(NC(=O)CN4C=CC=N4)=NC3=CC=C12	Virtual_Library **TIXc**	-8.6
**TI54c**	CN1N=C(Cl)C2=C3SC(NC(=O)CN4C=CN=C4)=NC3=CC=C12	Virtual_Library **TIXc**	-7.3
**TI55c**	CN1N=C(Cl)C2=C3SC(NC(=O)CN4C=NC=N4)=NC3=CC=C12	Virtual_Library **TIXc**	-9.4
**TI56c**	CN1N=C(Cl)C2=C3SC(NC(=O)CN(C4=CC=CC=C4)C4=CC=CC=C4)=NC3=CC=C12	Virtual_Library **TIXc**	-9.2
**TI57c**	COC1=CC=C(C=C1)N(CC(=O)NC1=NC2=CC=C3N(C)N=C(Cl)C3=C2S1)C1=CC=C(OC)C=C1	Virtual_Library **TIXc**	-9.9
**TI58c**	CN1N=C(Cl)C2=C3SC(NC(=O)CN4C5=C(C=CC=C5)C5=C4C=CC=C5)=NC3=CC=C12	Virtual_Library **TIXc**	-12.1
**TI59c**	COC1=CC2=C(C=C1)C1=C(C=C(OC)C=C1)N2CC(=O)NC1=NC2=CC=C3N(C)N=C(Cl)C3=C2S1	Virtual_Library **TIXc**	-9.6
**Template**	N1N=CC2=C3SC=NC3=CC=C12	Template	-7
**Donepezil**	COC1=CC2=C(C=C1OC)C(=O)C(CC1CCN(CC3=CC=CC=C3)CC1)C2	Positive control	-8.7
